# Competing mechanisms toughen bcc binary and medium-entropy alloys at 20 K

**DOI:** 10.1038/s41586-026-11065-7

**Published:** 2026-10-14

**Authors:** David H. Cook, Xiaoyu Chen, Michael O’Leary, Satish Rao, Wenqing Wang, Calvin H. Belcher, Madelyn Payne, Pedro Borges, Tung Lik Lee, Arun Devaraj, Mark Asta, Enrique J. Lavernia, Diran Apelian, Andrew M. Minor, Dong Liu, Punit Kumar, Robert O. Ritchie

**Affiliations:** 1 Department of Materials Science and Engineering, University of California, Berkeley, Berkeley, CA USA; 2 Materials Sciences Division, Lawrence Berkeley National Laboratory, Berkeley, CA USA; 3 Department of Physics, University of Bristol, Bristol, UK; 4 Department of Materials Science and Engineering, University of California, Irvine, Irvine, CA USA; 5 ISIS Neutron and Muon Source, Rutherford Appleton Laboratory, Didcot, UK; 6 Physical and Computational Sciences Directorate, Pacific Northwest National Laboratory, Richland, WA USA; 7 Department of Materials Science and Engineering, Texas A&M University, College Station, TX USA; 8 National Center for Electron Microscopy, Molecular Foundry, Lawrence Berkeley National Laboratory, Berkeley, CA USA; 9 Department of Engineering Science, University of Oxford, Oxford, UK; 10 School of Mechanical and Aerospace Engineering, Nanyang Technological University, Singapore, Singapore

**Keywords:** Structural materials, Mechanical engineering

## Abstract

Plastic deformation in body-centred cubic (bcc) metals and alloys is conventionally mediated by screw dislocations. Their temperature-sensitive motion is attributed to a steep rise in strength with decreasing temperature that constricts plastic zones at crack tips, leading to a ductile-to-brittle transition^1^. Here we evaluated the mechanical properties of group IV–V bcc refractory alloys with similar electronic structures at 20 K and found that these materials instead exhibit a plateau in tensile yield strength and fracture toughness as a function of temperature, with no apparent ductile-to-brittle transition. This behaviour arises from the global activation of twinning and an ω-phase transformation, which nucleate dislocations at interfaces before global dislocation motion. The initial mechanism is temperature insensitive and controls yielding, thereby preventing a crack-tip plastic zone from constricting with decreasing temperature.

## Abstract (web summary)

The mechanical properties of medium-entropy alloys at 20 K show a plateau in tensile yield strength and fracture toughness as a function of temperature, with no apparent ductile-to-brittle transition.

## Main

Alloys with the body-centred cubic (bcc) crystal structure have a ductile-to-brittle transition temperature (DBTT) below which plastic deformation is restricted, leading to embrittlement^1,2^. This problem has plagued bcc materials ranging from steels at or slightly below room temperature to refractory alloys up to 1,200 °C (refs. ^3,4^). The expansive set of current and potential applications for bcc alloys, such as refractory multi-principal alloys (commonly known as medium- or high-entropy alloys), for building and transportation infrastructure, energy technologies and hypersonic applications, is often limited by this transition^5,6^.

This limitation arises from their non-closed-packed crystal structure, which forces dislocations, specifically screws, to rely on thermally activated kink-pair nucleation to move^2,7^. Consequently, screw dislocation glide becomes sluggish at low temperatures, causing strength to increase precipitously and plasticity to be constricted at crack tips until fully brittle failure ensues^8^. Traditional strategies to suppress the DBTT primarily rely on facilitating screw dislocation glide through careful substitutional and interstitial alloying^9–12^: although interstitial elements increase DBTTs by introducing additional pinning stresses, substitutional alloying can, in some cases, ductilize bcc materials^11^. This has been observed in refractory binary and multi-principal element alloys, which are often extremely brittle^4,11,13^. Here concentrated additions of group IV (Ti, Zr, Hf) into group V (V, Nb, Ta) elements enhance screw dislocation glide, a phenomenon correlated with lowered unstable stacking-fault energies and increased kink-pair equilibrium concentrations^11,14^.

Beyond promoting dislocation glide, additional strategies for improving toughness involve activating multiple deformation mechanisms^15^. Twinning-induced plasticity (TWIP) and transformation-induced plasticity (TRIP) can elevate toughness by increasing strain hardening and uniform ductility^15,16^, while some softening mechanisms, such as kink banding, can also elevate toughness by promoting plasticity^17,18^. Although TWIP and TRIP mechanisms are highly effective at increasing cryogenic damage tolerance in face-centred cubic (fcc) alloys, their efficacy is limited in bcc alloys at low temperatures owing to fundamental differences in the mechanisms controlling nucleation, propagation and migration. This limitation arises from a competition between temperature-dependent dislocation glide stresses and more temperature-insensitive twinning stresses, which can converge at a slip-twinning transition^19–21^. Around this transition, it is hypothesized that the stress to transform ^1^/_2_<111> screw dislocation cores into three ^1^/_6_<111> fractional dislocations that bind a propagating twin embryo is lower than for the same dislocation to retain its compact core and glide^22,23^. This process can be accompanied by a phase transformation to the *ω*-phase, which can further decrease twin migration stresses and promote twin thickening^24^. Above this transition, twinning enhances ductility through the TWIP effect; however, near or below it, twins instead promote brittle failure by impinging on interfaces^25–27^. Even though twinning prevents further increases in yield stress (and therefore the constriction of the crack-tip plastic zone)^25,27^, the inherently high barrier to dislocation nucleation in alloys impedes the dissipation of damage at interfaces. For this reason, this transition causes brittle failure in tension (for example, in Fe–Si) and therefore is nearly always measured in compression, a stress state that closes cracks^25,28^.

Nevertheless, there is evidence that the slip-twinning transition can occur in alloys that do not exhibit a clear DBTT, such as Nb_45_Ta_25_Ti_15_Hf_15_ and an electronically similar, but chemically simpler, Nb_70_Ti_30_ alloy^17,29^. Previous work has demonstrated that Nb_45_Ta_25_Ti_15_Hf_15_ has exceptional strength and toughness across a wide temperature range owing to a combination of dislocation glide, twinning and kink-banding^17^. Together, these mechanisms sustain plasticity and suppress brittle failure, resulting in a fracture toughness of up to 90 MPa√m at 77 K. This combination of factors leaves the role of this transition in fracture behaviour in alloys that can retain tensile ductility at this mechanistic transition unclear. To understand the mechanical behaviour of these bcc alloys, we investigated their properties at even lower temperatures of approximately 20 K by performing uniaxial tensile tests and nonlinear elastic *J*-based fracture toughness tests, where the energy release rate, *J*, is measured as a function of crack extension, Δ*a*. In addition to measuring bulk mechanical properties, we performed extensive post-fracture electron backscatter diffraction (EBSD), fractography and transmission electron microscopy (TEM) to investigate the plastic deformation mechanisms and their spatio-temporal evolution with increasing strain and stress. We found that these bcc alloys nucleate dislocations instead of cracks at twin- and ω-boundary intersection sites, preventing premature brittle fracture and enabling the plastic zone to form via temperature-insensitive mechanisms.

## Mechanical behaviour at 20 K

**Extended Data Fig. 1:**
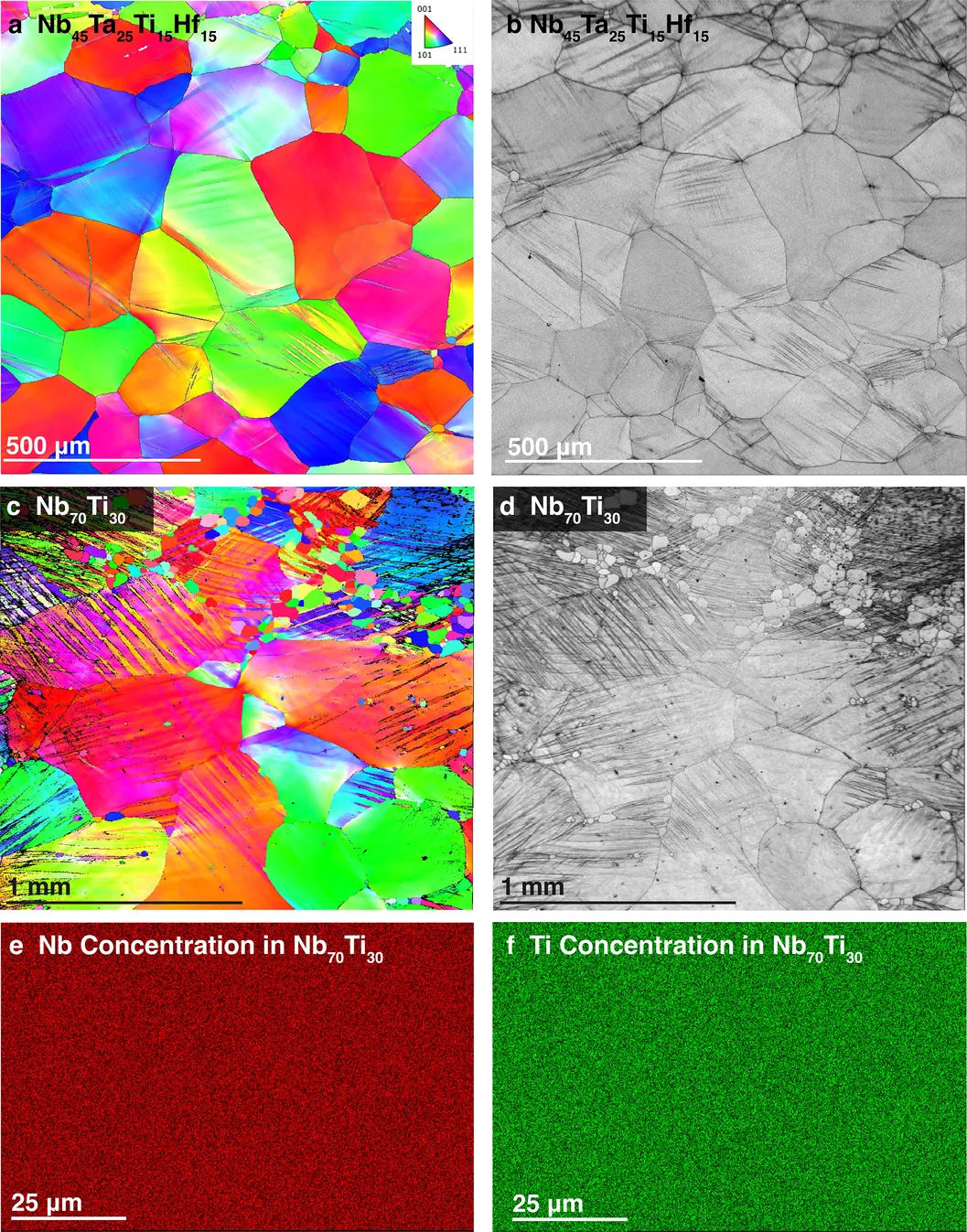
Microstructure of Nb_45_Ta_25_Ti_15_Hf_15_ and Nb_70_Ti_30_. (**a**) IPF maps and (**b**) band contrast (BC) maps of Nb_45_Ta_25_Ti_15_Hf_15_ showing a microstructure consisting of equiaxed grains ~259 μm in diameter. Some grains are not fully recrystallized because they contain sub-grain boundaries of partially recrystallized kink bands formed during rolling. (**c**) IPF maps and (**d**) BC maps of Nb_70_Ti_30_ showing the heterogeneous microstructure consisting of smaller, fully recrystallized grains ~51 μm in diameter and larger grains containing partially recrystallized kink bands and rotational twins. (**E**) and (**F**) show EDX maps indicating homogeneous composition.

As described in more detail in Supplementary Information, the Nb_45_Ta_25_Ti_15_Hf_15_ and Nb_70_Ti_30_ alloys were arc-melted, forged and cross-rolled to a 33% reduction in thickness, and heat-treated at approximately 56% of their solidus temperature for 1 hour to produce a single-phase, near-fully recrystallized, equiaxed grain structure with an average grain size of approximately 259 μm for Nb_45_Ta_25_Ti_15_Hf_15_ and a heterogeneous, bimodal distribution of approximately 51–412 μm for Nb_70_Ti_30_ (Extended Data Fig. 1). Both alloys contain low interstitial concentrations: Nb_45_Ta_25_Ti_15_Hf_15_ has 0.059 at% (60 ppmw) C, 0.0495 at% (58 ppmw) N, and 0.146 at% (197 ppmw) O; Nb_70_Ti_30_ has 0.278 at% (561 ppmw) O and 0.0625 at% (111 ppmw) N (ref. ^17^).

**Fig. 1:**
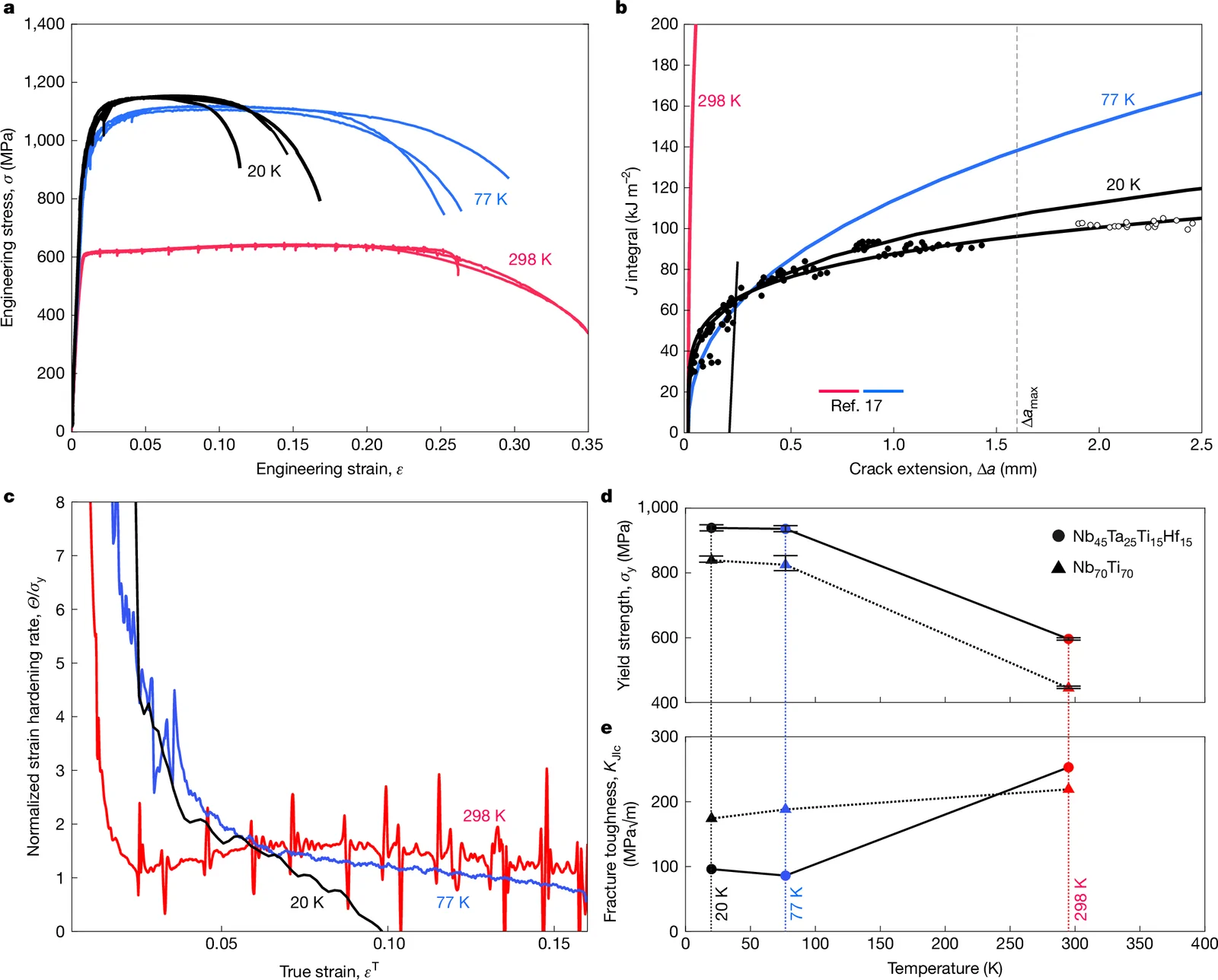
Mechanical properties of NbTi-based alloys. **a**, Tensile engineering stress–strain curves of Nb_45_Ta_25_Ti_15_Hf_15_ with yield strength plotted as a function of temperature (*n* = 3) in **d** and instantaneous normalized strain-hardening rate, (*θ*/*σ*_y_), vs. true strain, *ε*^T^, curves extracted in **c**. **b**, *J*–*R* curves (*n* = 2) show the resistance to fracture of the Nb_45_Ta_25_Ti_15_Hf_15_ alloy as a function of crack extension. Corresponding *K*-based fracture toughness values (crack-initiation toughness, *K*_JIc__,_ and steady-state crack-growth toughness, *K*_ss_) back-calculated from **b** are shown in **d** as a function of temperature. The error bars in **d** are given as the standard deviation across the three tensile tests, and the values in **e** are reported as the mean across the two fracture toughness tests. Both values met the criteria for *J* dominance and plane-strain conditions. It is noted that the slightly lower crack-initiation toughness (*K*_JIc_) at 77 K compared with 20 K in Nb_45_Ta_25_Ti_15_Hf_15_ is attributed to the statistical variation related to processing defects and microstructure in the tested samples. Source data

**Extended Data Fig. 2:**
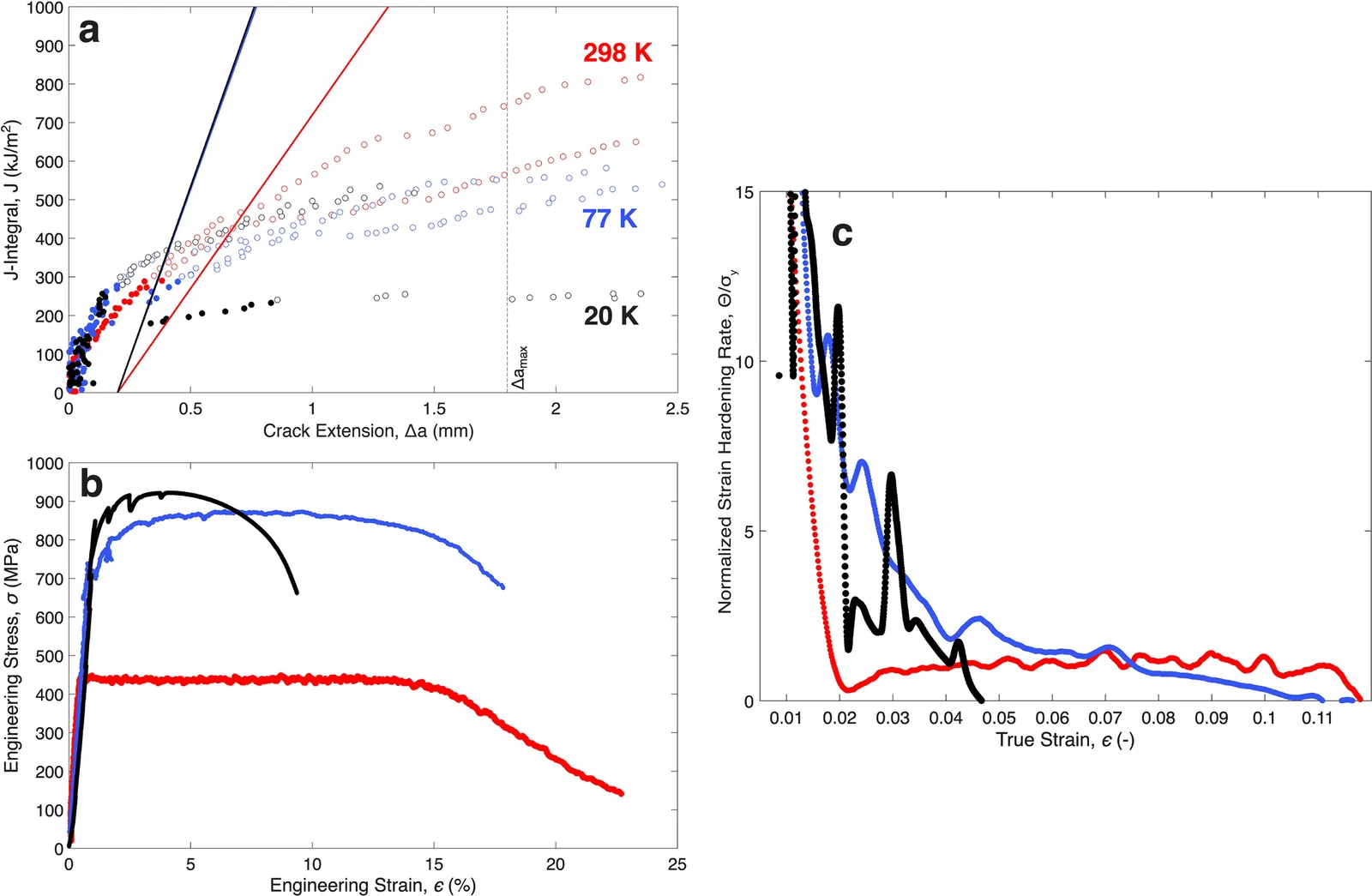
Mechanical Properties of Nb_70_Ti_30_. (**a**) R-curves showing that the fracture toughness from 298 K to 20 K gradually decreases. (**b**) Representative stress-strain curves across the same set of temperatures show a gradual reduction in ductility as well as twinning-induced stress drops at temperatures at and below 77 K. (**c**) Instantaneous strain-hardening curves demonstrate similar strain-hardening behavior as Nb_45_Ta_25_Ti_15_Hf_15_: hardening becomes less sustained at progressively decreasing temperatures as a result of twin activation at yielding. Source data

Representative tensile stress–strain curves and crack-resistance curves (*R* curves) measured in terms of the *J* integral (kJ mm^−2^) as a function of crack extension, Δ*a* (mm), are shown in Fig. 1a,b for Nb_45_Ta_25_Ti_15_Hf_15_ and in Extended Data Fig. 2 for Nb_70_Ti_30_. The corresponding tensile stress–strain curves and *R* curves at 77 K and 298 K are also shown for comparison^17^. In Nb_45_Ta_25_Ti_15_Hf_15_, the yield strength at 20 K (*σ*_y_ ≈ 938.9 ± 22.6 MPa) is comparable to that measured at 77 K (*σ*_y_ ≈ 936.6 ± 22.4 MPa) (Fig. 1d). The ultimate tensile stress (*σ*_UTS_ ≈ 1,149.5 ± 3.4 MPa) is approximately 35 MPa higher than that measured at 77 K, whereas the uniform ductility and the elongation to fracture decrease by approximately 33% and approximately 50%, respectively^17^. A similar effect occurs in Nb_70_Ti_30_: the yield strength at 77 K (*σ*_y_ ≈ 825.8 ± 49.4 MPa) is only 14 MPa lower than at 20 K (*σ*_y_ ≈ 839.9 ± 18.5 MPa), within the statistical variation. In both alloys, yielding at cryogenic temperatures is accompanied by stress drops and acoustic emission consistent with twinning. An evaluation of the instantaneous strain hardening in Nb_45_Ta_25_Ti_15_Hf_15_ (Fig. 1c) illustrates that at 298 K the hardening remains high at all strains, but with decreasing temperature, it is continuously less sustained with increasing strain. In the presence of a crack, the material exhibits increasing *R*-curve behaviour, that is, its resistance to fracture increases with increasing crack extension (Fig. 1b). The size-independent crack-initiation toughness, *K*_JIc,_ and crack-growth toughness, *K*_ss_ (defined as the toughness at maximum allowable crack extension), are calculated from the *R* curves^30^ (Methods). In Nb_45_Ta_25_Ti_15_Hf_15_, the crack-initiation toughness, *K*_JIc_, is approximately 96.5 MPa√m, and the steady-state crack-growth toughness, *K*_ss_, is approximately 118 MPa√m (Fig. 1e) at 20 K. Similarly, Nb_70_Ti_30_ has a crack-initiation toughness of 170 MPa√m at 20 K, showing little change from 188 MPa√m and 219 MPa√m at 77 K and 298 K, respectively. These properties are summarized in Extended Data Table 1.

## Deformation mechanisms at 20 K

**Fig. 2:**
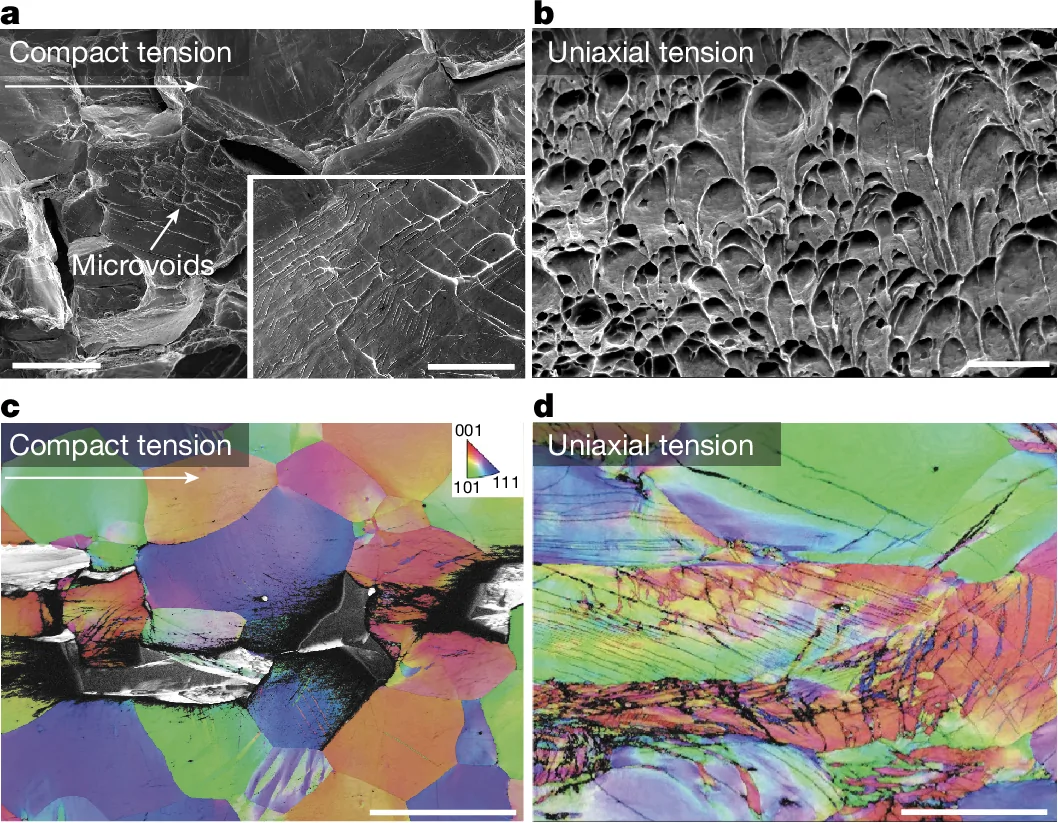
Fracture morphology and EBSD maps of Nb_45_Ta_25_Ti_15_Hf_15_. **a**–**d**, The effect of stress state is illustrated by differences in fracture morphology observed between compact-tension and uniaxial tensile samples. In compact-tension specimens (**a**), fracture occurs by crystallographic microvoids on grain boundaries, whereas in tensile specimens (**b**), it occurs by microvoid coalescence. This is further exemplified by EBSD IPF and band-contrast maps that show profuse {112}⟨111⟩ twinning in compact-tension (**c**) and tensile (**d**) specimens but significantly more deformation by dislocation glide in tension in **d**. Scale bars, 100 μm (**a**), 40 μm (**a**(inset)), 5 μm (**b**), 500 μm (**c**), 200 μm (**d**).

**Extended Data Fig. 3:**
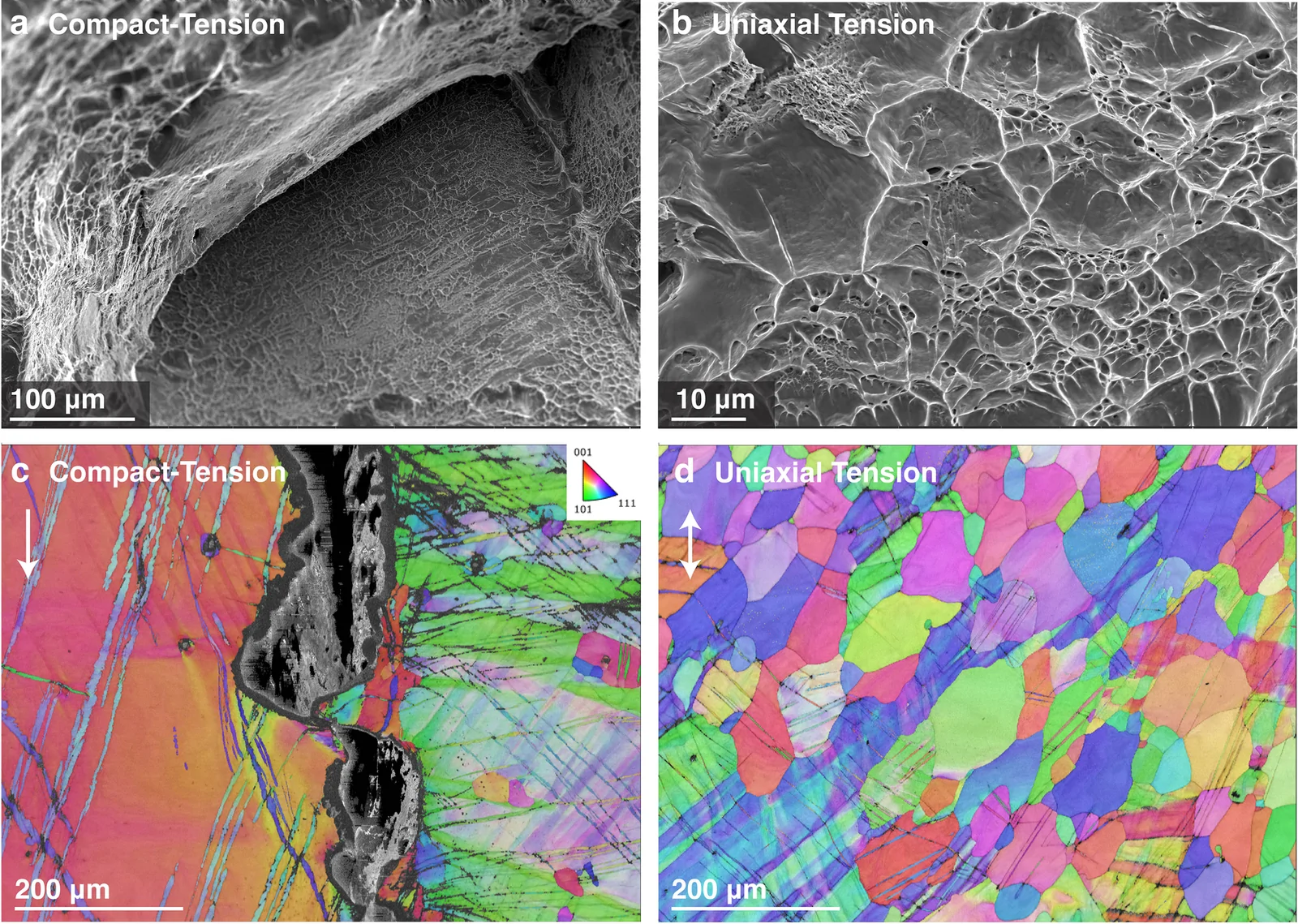
Fracture morphology and EBSD maps of Nb_70_Ti_30_. The effect of stress-state is illustrated by the differences in fracture morphology observed between C(T) and uniaxial tensile samples. Whereas in C(T) specimens in (**a**) fracture occurs by crystallographic microvoids on grain boundaries, in tensile specimens in (**b**) it occurs by microvoid coalescence. This is further exemplified by IPF and BC EBSD maps that show profuse {112} < 111> twinning in (**c**) compact-tension and (**d**) tensile specimens, but with significantly more deformation by dislocation glide in tension in (**d**). The intergranular fracture can also be noted in (**c**), where the crack travels along the high-angle grain boundary between two large grains.

Despite the high toughness and ductility at 20 K, the fracture surfaces in both alloys show differences between tensile and compact-tension specimens used for the fracture tests (Fig. 2a,b, Supplementary Figs. 1 and 2, and Extended Data Fig. 3). Here it is important to note that in the vicinity of a crack, there are three principal stresses that are each approximately three to five times higher than the one principal stress in tensile specimens. The resultant triaxial stress state generates a hydrostatic stress, suppresses shear stresses and is additionally graded^31^. Whereas in uniaxial tension, fracture is fully ductile and occurs by microvoid coalescence, under crack-tip stress triaxiality, there are some signs of brittle features. Specifically, fracture is partly intergranular and partly ductile, with crystallographically oriented bands and microvoids along grain boundaries, a transition that departs from fully ductile fracture at higher temperatures. The differences generated between the two stress states are further illustrated by EBSD band-contrast and inverse-pole-figure (IPF) maps of tensile samples and at the mid-thickness of compact-tension samples, where plane-strain conditions and hydrostatic stresses are highest (Fig. 2c,d). Although both samples exhibit profuse {112}⟨111⟩ nano-twinning, tensile samples have higher levels of dislocation glide than compact-tension samples. In the latter, there are some kink bands (significantly fewer than at higher temperatures), indicating edge dislocation glide.

**Fig. 3:**
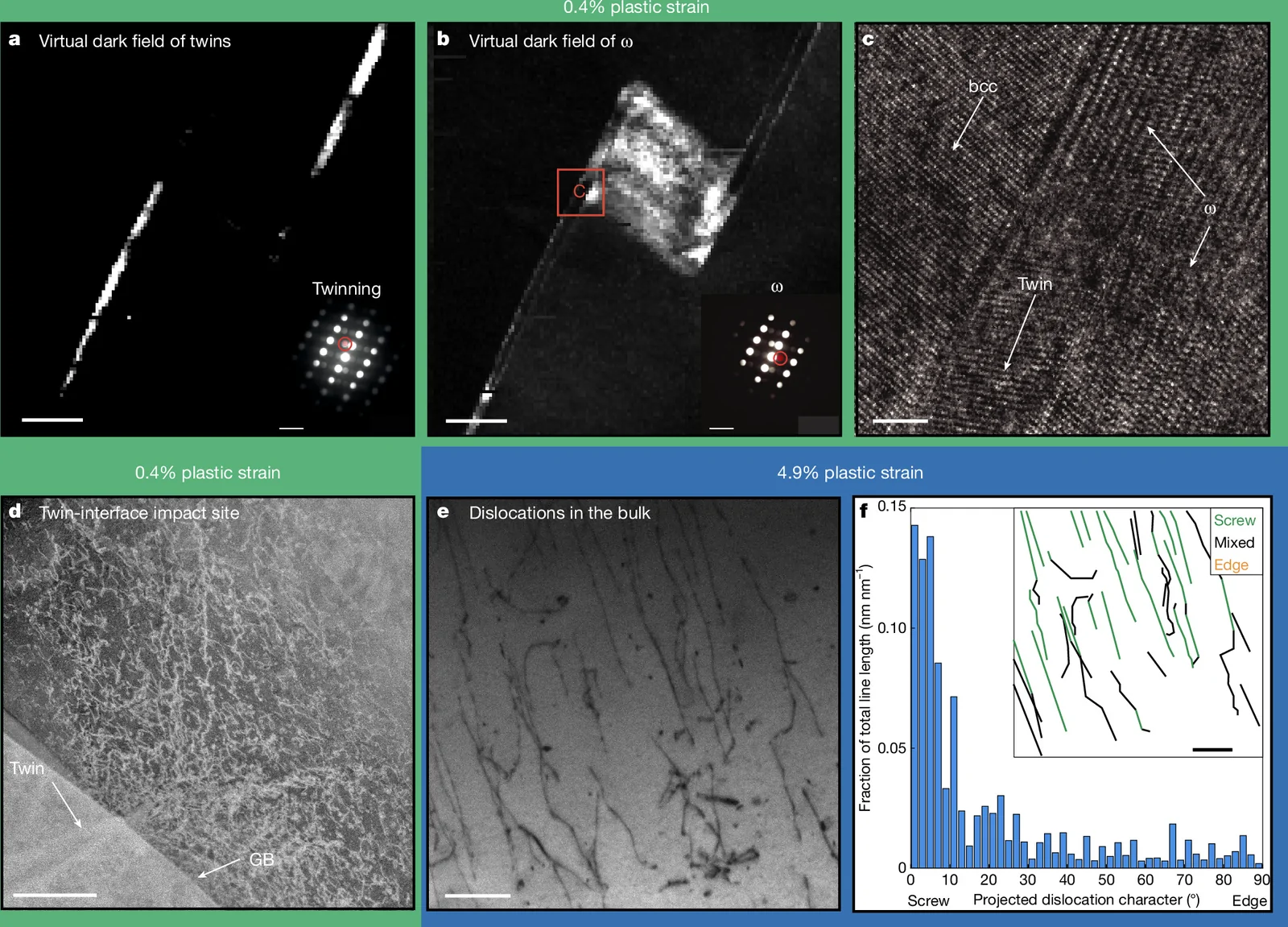
Sequence of uniaxial tensile deformation mechanisms. **a–c**, Virtual dark-field images reconstructed from 4D-STEM scans of two twins with ω-phase collapse occurring between them. The red circles in the inset diffraction patterns indicate the positions of virtual apertures which reveal the location of twins in **a** and the ω-phase in **b**. Panel **c** shows an HRTEM image of the red box in **b**. The ω-phase is additionally observed in **b** as a transitional twin boundary structure. **d**,**e**, Once the twins propagate, they impact interfaces such as grain boundaries (GBs; **d**), generating a local stress concentration that nucleates mixed dislocations. At higher plastic strains, the dislocations can glide into the grain interiors (**e**). **f**, A **g** ∙ **b** analysis demonstrates that they adopt screw character, illustrated in the inset. It is noted that the character of screw and mixed dislocations is determined under the assumption that the sample has negligible thickness. Scale bars, 20 nm (**a**,**b**), 5 nm^−1^ (**a**(inset), **b**(inset)) 2 nm (**c**), 0.5 μm (**d**), 100 nm (**e**,**f**(inset)). Source data

**Extended Data Fig. 4:**
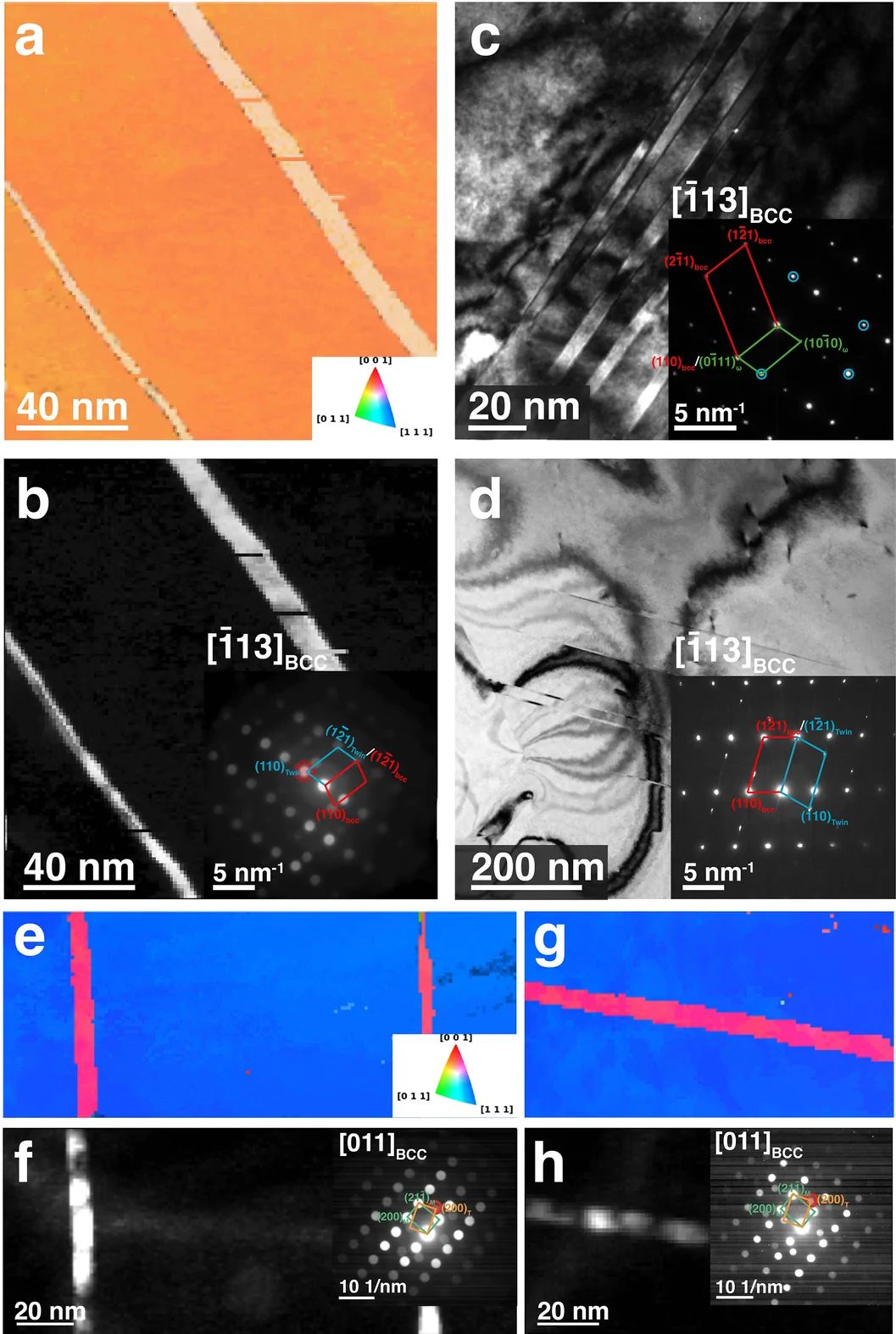
Tensile yielding twins with interfacial ω at 20 and 77 K in Nb_45_Ta_25_Ti_15_Hf_15_. (**a**) Orientation maps of twins at 20 K taken from a 4D-STEM scan. (**b**) Corresponding virtual dark-field reconstructions of the same twins in (**a**) with the virtual dark-field detector highlighted in red. (**c**) TEM micrograph of twins encased by ω. The corresponding diffraction pattern of the $[\bar{1}13]$ *bcc* zone axis (ZA) shows significantly higher intensities from peaks shared by twins and ω (circled in blue) than those solely from ω. (**d**) Another instance of twinning at 20 K. Dislocations can be seen impinging on the twin boundaries. At 77 K, orientation maps reconstructed from 4D-STEM micrographs show two instances of twinning in (**e**) and (**g**) with corresponding virtual dark-field images reconstructed in (**f**) and (**h**).

**Extended Data Fig. 5:**
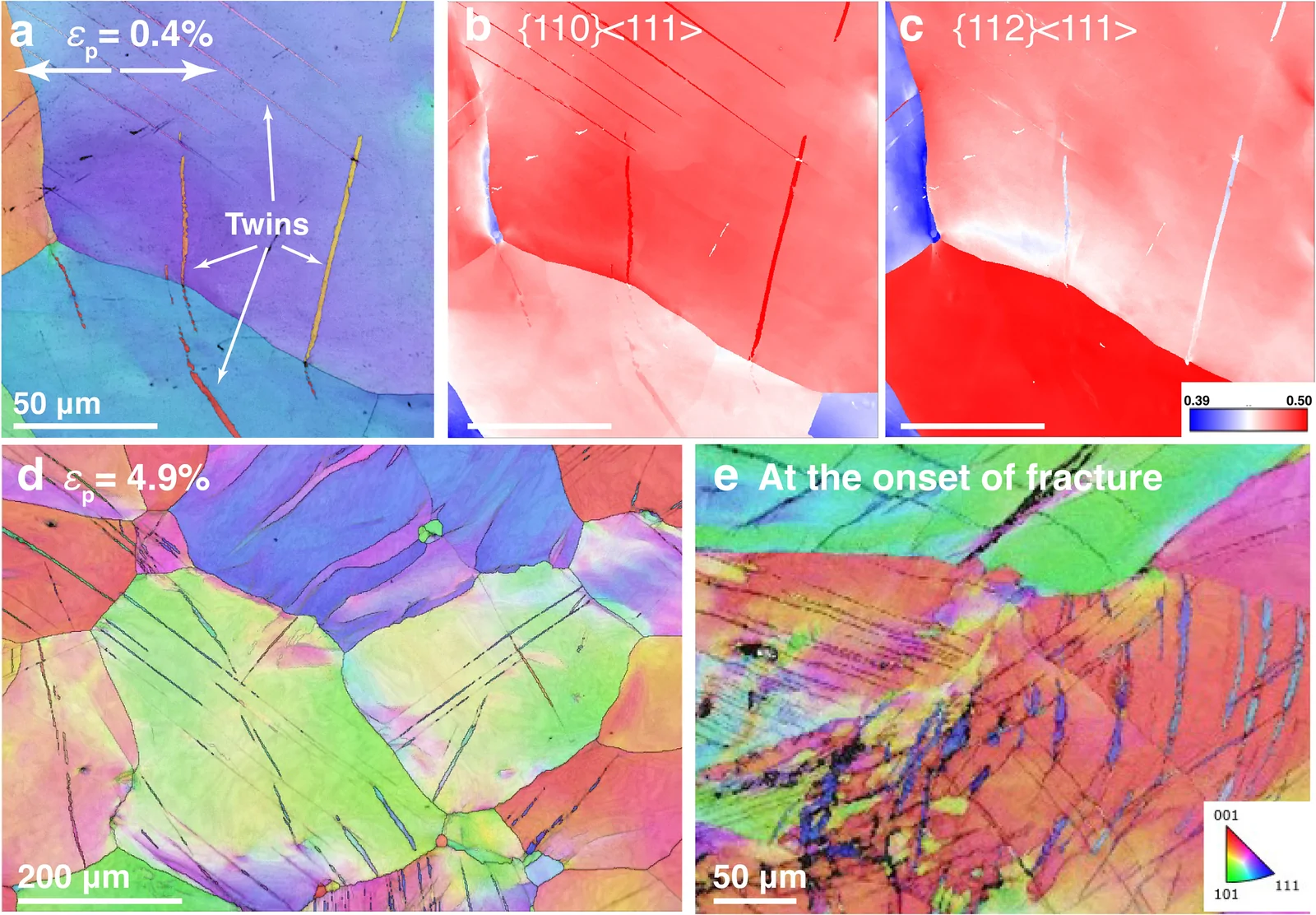
Sequence of tensile deformation mechanisms in Nb_45_Ta_25_Ti_15_Hf_15_. (**a**) Shows twinning at the onset of yielding, which progressively gives way to deformation by dislocation glide in (**d**). (**b**)-(**c**) A simple Schmidt factor analysis of the microstructure shows that the twins can orient each lamella into directions with varying critical resolved shear stress for both a_0_/2{110} < 111> and a_0_/2{112} < 111> slip systems. While in a typical TWIP material, this would generate strain hardening, here dislocation glide is difficult and plastic deformation has already begun, so only dislocations with the lowest barriers to glide (those with the highest critical resolved shear stresses) will move, causing strain to localize in the material. Severe deformation in the region that necked and directly adjacent to the fracture surface can be seen in (**e**). Note that the kink band-like features in (**d**) are part of the original microstructure.

To investigate the microscopic deformation mechanisms underlying this atypical mechanical behaviour, we first analysed the sequence of tensile deformation mechanisms. Tensile specimens of Nb_45_Ta_25_Ti_15_Hf_15_—one interrupted shortly after yielding $({\varepsilon }_{{\rm{p}}}^{{\rm{T}}}=0.004,{\sigma }^{{\rm{T}}}\approx \mathrm{1,013}\,\mathrm{MPa})$ and a second at a higher uniform strain $({{\rm{\varepsilon }}}_{{\rm{p}}}^{{\rm{T}}}=0.049,{{\rm{\sigma }}}^{{\rm{T}}}\approx 1,219\,{\rm{M}}{\rm{P}}{\rm{a}})$, ${\rm{w}}{\rm{h}}{\rm{e}}{\rm{r}}{\rm{e}}\,{{\rm{\varepsilon }}}_{{\rm{p}}}^{{\rm{T}}}\,{\rm{i}}{\rm{s}}\,{\rm{t}}{\rm{h}}{\rm{e}}\,{\rm{t}}{\rm{r}}{\rm{u}}{\rm{e}}\,{\rm{p}}{\rm{l}}{\rm{a}}{\rm{s}}{\rm{t}}{\rm{i}}{\rm{c}}\,{\rm{s}}{\rm{t}}{\rm{r}}{\rm{a}}{\rm{i}}{\rm{n}}$—were jet polished and examined in a transmission electron microscope. These experiments reveal that twinning coupled with a phase transformation to the ω-phase initiates yielding. Virtual dark-field reconstructions of four-dimensional scanning transmission electron microscopy (4D-STEM) scans correlated with high-resolution TEM (HRTEM) images of tensile specimens interrupted at 0.4% plastic strain (Fig. 3a–c and Extended Data Fig. 4a–d), show conventional {112}⟨111⟩ nanotwins, with a nanometre-thick interfacial layer of ω-phase that expands in the space between the twins. Indexed diffraction patterns are shown in Supplementary Fig. 3. We also observe twinning controlling yielding at 77 K (Extended Data Fig. 4g,h). We did not observe any other twinning systems. Although it is unclear whether the crystal collapsed to the ω-phase first to aid twin nucleation second, or if the local shear stress from the twin embryo growth and propagation triggered the ω-phase transformation (Fig. 3b,c), in either case, this phase can assist twin nucleation and growth^24,32–34^. We also tracked the twins to their termination sites at interfaces. Here twins produce stress concentrations high enough to nucleate dislocations (Fig. 3d). As deformation progresses to the uniform strain, these dislocations (invariably combined with pre-existing ones in the undeformed material) select more sluggish screw orientations to mediate plastic flow at higher strains (Fig. 3e,f and Supplementary Fig. 4). This sequence is summarized in EBSD maps in Extended Data Fig. 5.

**Fig. 4:**
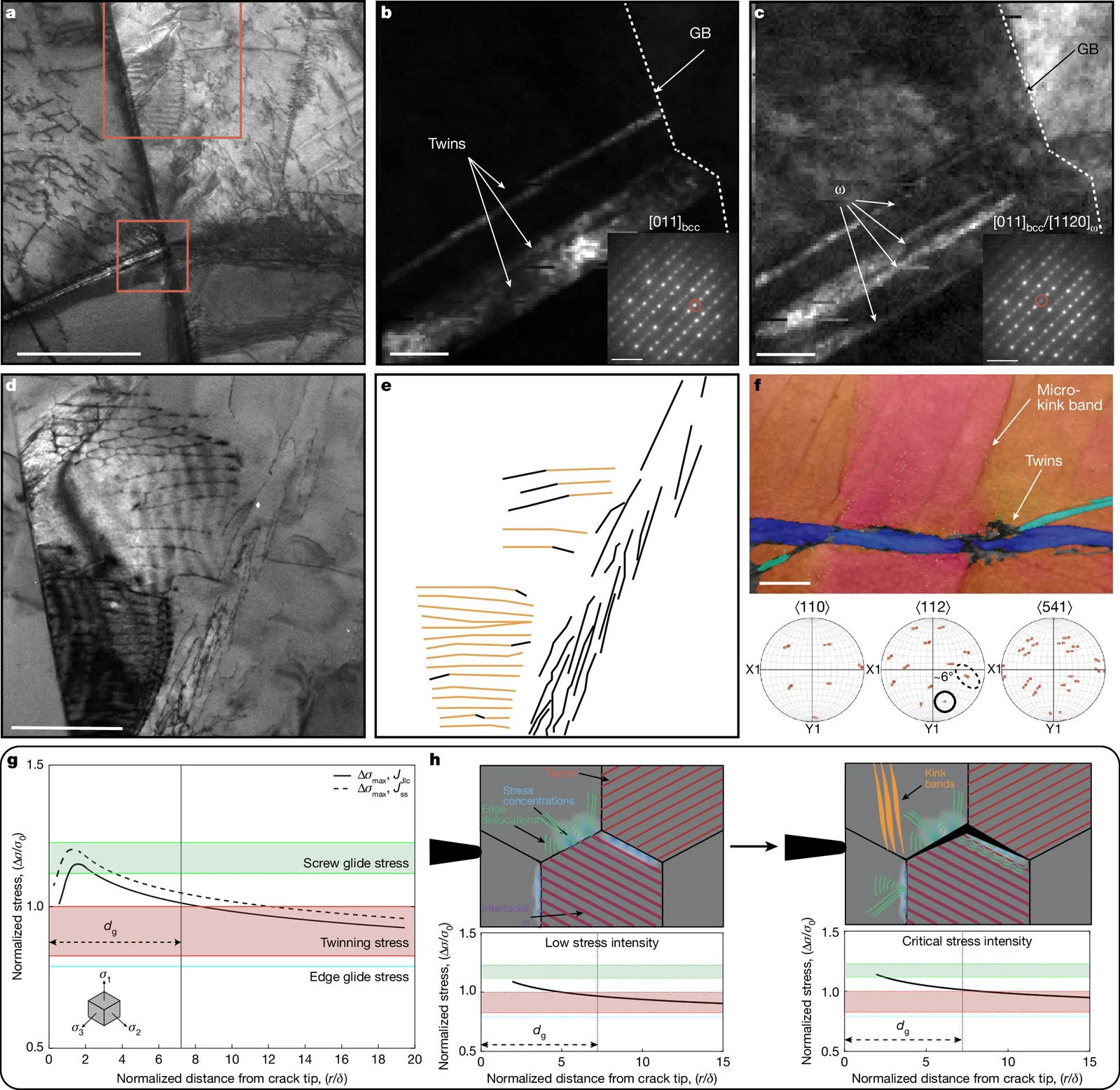
Crack-tip twinning, ω-phase and dislocations in Nb_45_Ta_25_Ti_15_Hf_15_. **a**, TEM overview micrograph of a fracturing grain boundary approximately 30 μm ahead of the cracked ligament. The area indicated by the top red box is shown in **d** and **e**; the area indicated by the bottom red box is shown in **b** and **c**. **b**, Virtual dark-field 4D-STEM scans reconstructed from the circled diffraction disk indicated in the inset show that the deformation at the grain boundary is produced by twinning. **c**, Phase map reconstructed from a virtual detector (highlighted in red) placed over an ω-phase diffraction disk that shows the hexagonal phase on twin boundaries. **d**, STEM image showing that the stress concentration produced by twins and the ω-phase nucleates dislocations. **e**,**f**, A **g** ∙ **b** analysis (**e**) reveals some dislocations in **d** are edges and can travel to form micro-kink bands (**f**). **g**, These mechanisms are illustrated in a schematic that shows which part of the HRR field they create. These plots demonstrate that, unlike in tensile samples, the constraint imposed by stress triaxiality does not allow the stresses to become high enough to substantially activate dislocation glide. **h**, As a result, fracture occurs in a brittle-like process, where the stresses exceed the twinning stresses across a critical microstructural length scale, *d*_g_, the grain size (which is normalized by *δ*, the crack-tip opening displacement (CTOD)), at the critical stress intensity. It is noted that the character of mixed dislocations is determined under the assumption that the sample has negligible thickness. Scale bars, 1 μm (**a**), 50 μm (**b**,**c**), 10 nm^−1^ (**b**(inset), **c**(inset)), 500 nm (**d**), 1 μm (**f**). Source data

**Extended Data Fig. 6:**
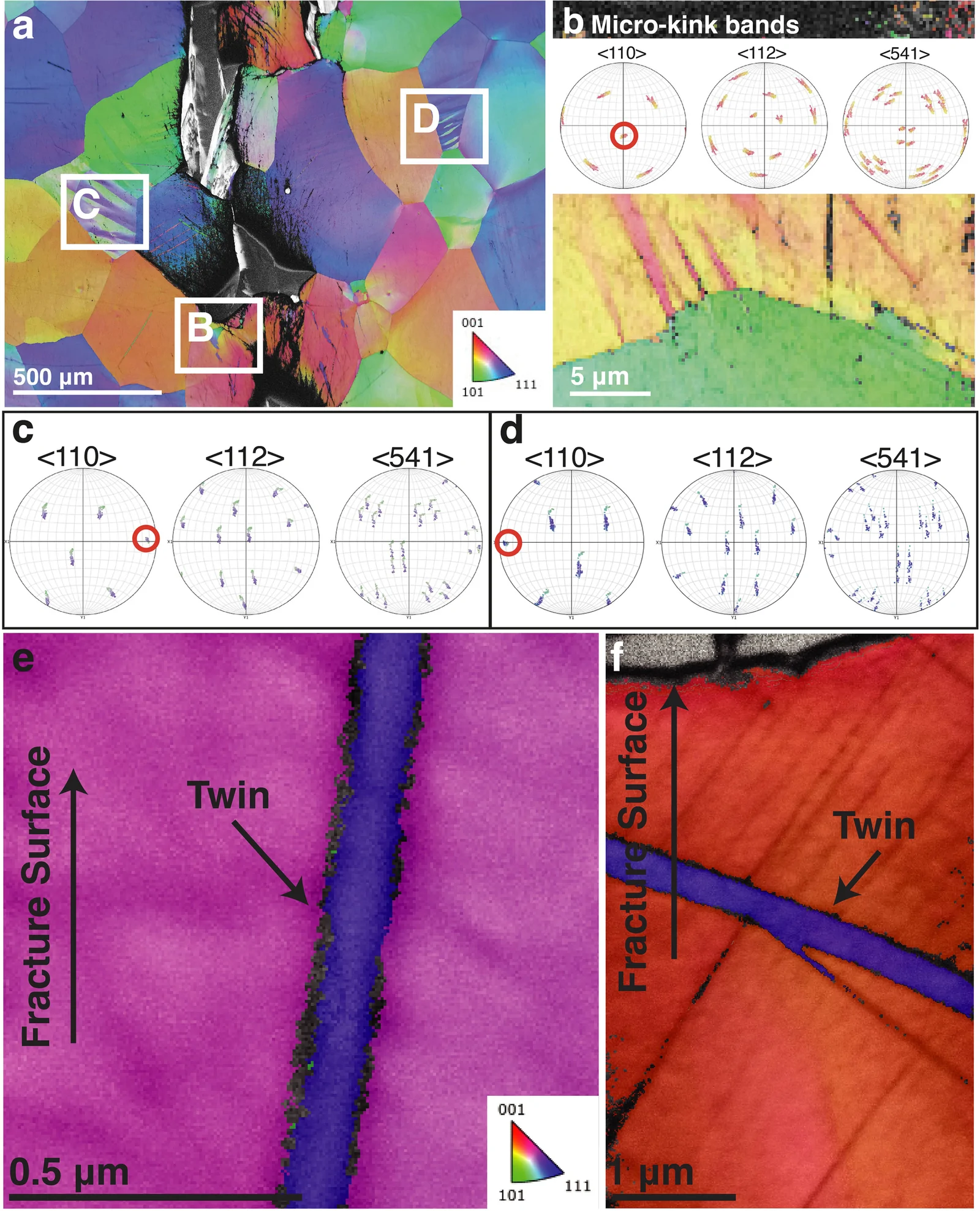
Crack-tip kink deformation mechanisms in Nb_45_Ta_25_Ti_15_Hf_15_. (**a**) Overview EBSD map of crack-tip deformation structure with kink bands boxed in (**b**), (**c**), and (**d**). Pole figure maps of the kink bands in the selected areas in (**a**) (that contain kink bands) show that all have <110> poles, indicating that they are formed by a_0_/2 < 111 > {112} edge dislocations. The bands have variable rotation: those in (**b**) 8–15°, those in (**c**) 5–17°, and those in (**d**) 10–22°. These kink bands can also have gradient boundaries, indicating imperfect edge dislocation alignment, and can be micrometer-scale in size, as shown in (**b**). (**e**)**-**(**f**) show TKD micrographs of twins impinging on fractured grain boundaries at the fracture surface. The twin in (**e**) has an unusual morphology characterized by branching.

To address how this global sequence of tensile deformation mechanisms (we use the word ‘global’ to exclude cases of microplasticity in which local regions may favour dislocation glide before twinning) affects the fracture properties, we investigated compact-tension samples at the mid-thickness (where the stress triaxiality would be highest owing to plane-strain conditions) by transmission Kikuchi diffraction (TKD) and 4D-STEM. Although the EBSD maps in Fig. 2 provide an overview of the crack-tip deformation mechanisms, it remains unclear why the fracture is nominally intergranular. To identify the cause, we performed TKD and 4D-STEM on regions near grain boundaries to capture the deformation mechanisms occurring in the regions of highest stress before crack extension. Focused-ion-beam lift-outs were taken from a grain boundary several micrometres ahead of a crack tip and from an intergranular fracture facet with the distinctive crystallographic deformation markings in Fig. 2a. Precise calculation of stress and strain using the Hutchinson–Rice–Rosengren (HRR) crack-tip plastic strain field is detailed in Methods. Reconstructed virtual dark-field images of 4D-STEM maps in Fig. 4a–c with indexed diffraction patterns in Supplementary Fig. 5 show similar deformation structures to those seen at yielding in tensile samples: fine nanotwins accompanied by an ω-phase impinging on a grain boundary. Additional instances of twins forming the crystallographic microvoids in Fig. 1e on fractured grain boundaries are documented in Extended Data Fig. 6. However, any intergranular fracture that it could cause is delayed by dislocations nucleated in the parent and neighbouring grain, some of which were identified as edge and mixed dislocations by a **g** ∙ **b** analysis, where **g** is the diffraction vector and **b** is the Burgers vector (Fig. 4d,e and Supplementary Fig. 6). The shear stresses are high enough for edge dislocations to glide and form microscale kink bands, as we observed with TKD in regions close to the fracture surface^35^ (Fig. 4f and Extended Data Fig. 6). Other dislocations form channels along which deformation is localized (Fig. 4a).

## Discussion

Our results indicate that nonlinear interactions among four deformation mechanisms—twinning, phase transformations, dislocation glide and dislocation nucleation—can lead to competitive behaviour among them as a function of temperature. This manifests below 77 K as a global sequence of tensile deformation mechanisms that nominally begins with twinning or a phase transformation and ends with dislocation glide. However, a pervasive question remains as to why the slip-twinning transition inhibits the DBTT in these NbTi alloys but causes low-temperature embrittlement in all other bcc materials observed so far^27^.

**Extended Data Fig. 7:**
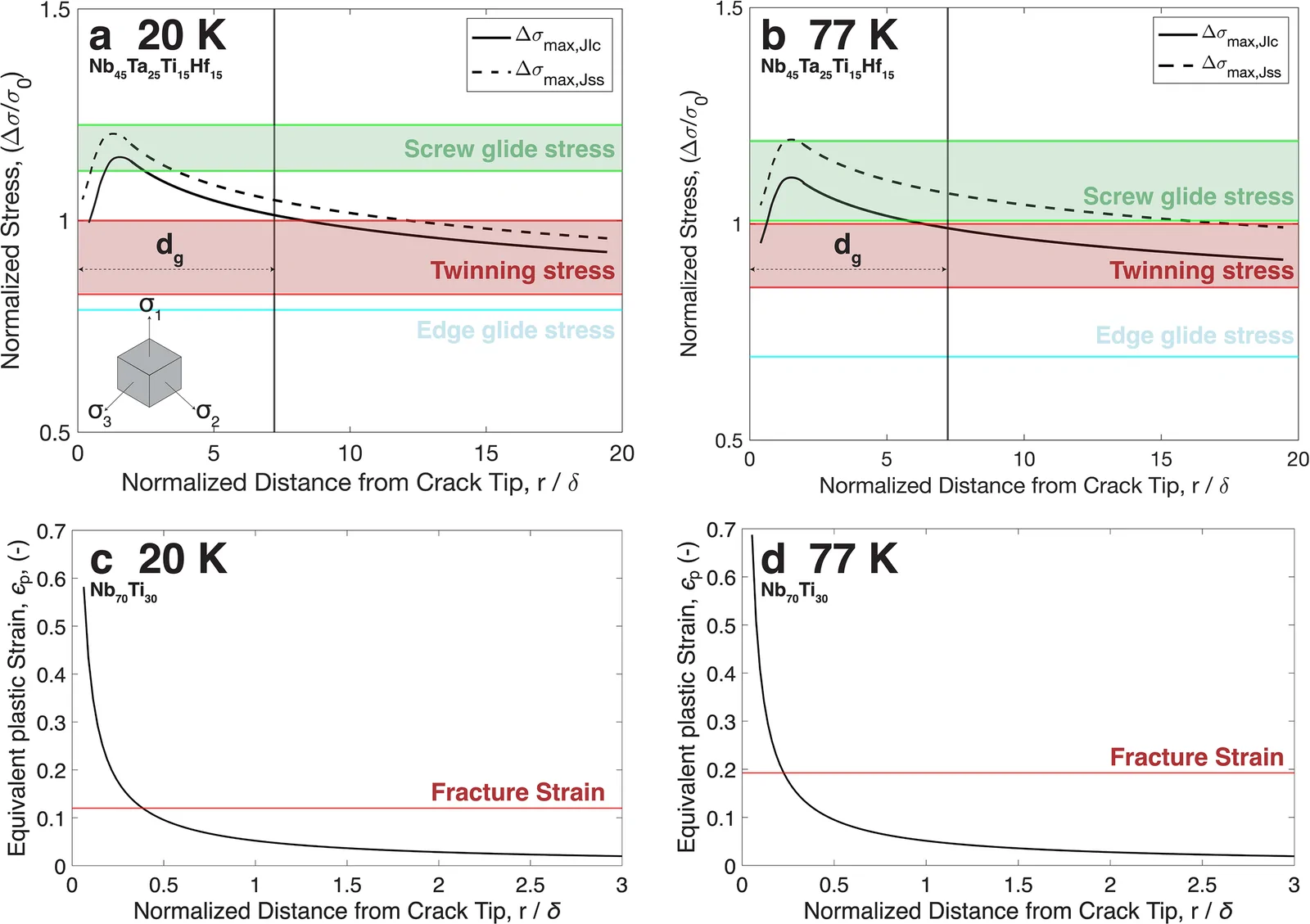
HRR Stress fields ahead of a crack in Nb_45_Ta_25_Ti_15_Hf_15_ and Nb_70_Ti_30_. (**a**) A plot of the HRR field at 20 K as a function of distance from the crack tip normalized by the crack-tip opening displacement, *δ*. These values are compared with twinning growth stresses and screw and edge glide stresses. As can be seen, the applied stress at the critical stress intensity exceeds the twinning stress across a critical microstructural length-scale - a single grain size. The same occurs at 77 K in (**b**), demonstrating that the crack-initiation toughness is insensitive to temperature. However, at 77 K, screw glide stresses are lower, which causes this mechanism to activate during crack growth when the stress intensity increases. (**c**)-(**d**) show the local equivalent plastic strain as a function of distance from the crack tip. In Nb_70_Ti_30_, fracture is strain-controlled because both twinning stresses and dislocation glide stresses are lower than the fracture stress across a critical microstructural length-scale. Source data

**Extended Data Fig. 8:**
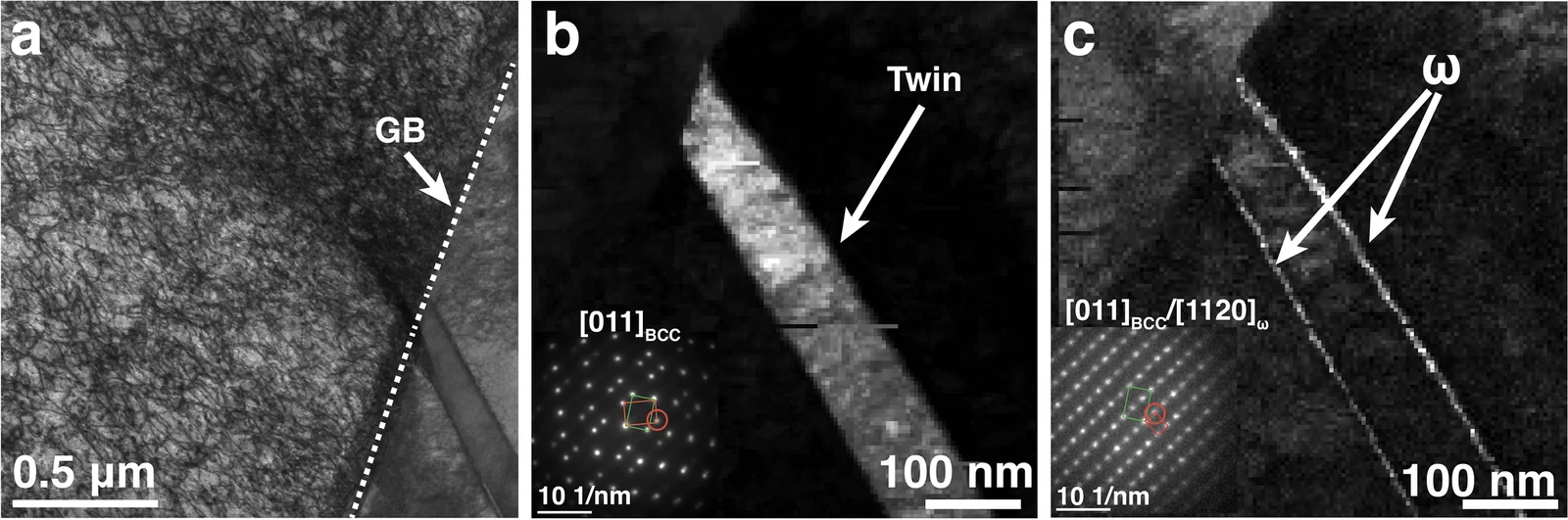
Crack-tip twinning, omega, and dislocations in Nb_70_Ti_30_. (**a**) HAADF-STEM overview micrograph of a twin-grain boundary intersection site ahead of a crack tip. (**b**) Virtual dark-field 4D-STEM scans reconstructed from the circled diffraction disk indicated in (**b**) inset show that the deformation at the grain boundary is produced by twinning; a phase map in (**c**) reconstructed from a virtual detector (highlighted in red) placed over an ω diffraction disk that shows the hexagonal phase on twin boundaries. Unlike in Nb_45_Ta_25_Ti_15_Hf_15_, the dislocation density is significantly higher, indicating that chemical complexity can substantially increase barriers to dislocation glide and restrict crack-tip plasticity.

To discern this variable, we used experimental and computationally measured values to quantify each mechanism. First, we obtained twinning and dislocation glide stresses for both alloys. Twinning stresses were obtained from tensile curves, whereas screw (with the original compact core) and edge dislocation tensile glide stresses were calculated using, respectively, the Rao–Suzuki model and the Maresca–Curtin model at 20 K with an experimental strain rate of 1 × 10^−3^ s^−1^ (refs. ^36–38^). These values are listed in Table 1. Athough the tensile glide stresses are only about 30% higher in Nb_45_Ta_25_Ti_15_Hf_15_ compared with Nb_70_Ti_30_, these apparently small differences in uniaxial tension can be exacerbated at a crack tip by the high hydrostatic stresses^3^. This is easily visualized by mapping these stresses onto the HRR fields in Fig. 4g,h and Extended Data Fig. 7. As can be seen in Fig. 4g, the local shear stresses at the crack tip are high enough to activate twinning in Nb_45_Ta_25_Ti_15_Hf_15_ and Nb_70_Ti_30_, but they are not high enough in Nb_45_Ta_25_Ti_15_Hf_15_ to move pre-existing dislocations, except for within a small region close to the crack tip. Lower glide barriers in Nb_70_Ti_30_ generate a strain-controlled fracture, as evidenced by the high and homogeneous dislocation distributions (despite local damage produced at twin/ω-phase interface sites) (Extended Data Fig. 8) and increased ductile fracture surface features in compact-tension samples (Extended Data Fig. 3).

**Table 1:** A summary of mechanisms observed in each stress state

Stress state	Alloy	Screw dislocations	Edge dislocations	{112}⟨111⟩ twinning	ω-phase transformation	Kink bands
Uniaxial tension (activation stress)	Nb_45_Ta_25_Ti_15_Hf_15_	Yes (2) 1,048–1,384 MPa (20–10b)	No 740 MPa	Yes (1) 775–938 MPa	Yes (1)	No
Crack-tip stress triaxiality	Nb_45_Ta_25_Ti_15_Hf_15_	No	Yes (2)	Yes (1)	Yes (1)	Yes (3)
Uniaxial tension (activation stress)	Nb_70_Ti_30_	Yes (2) 1,080 MPa (10b)	— 105 MPa	Yes (1) 473–840 MPa	—	—
Crack-tip stress triaxiality	Nb_70_Ti_30_	— (2)	—	Yes (1)	Yes (1)	—

Their sequential order with increasing stress is indicated in parentheses. Mechanisms that are not directly observed are indicated with em dashes.

**Extended Data Fig. 9:**
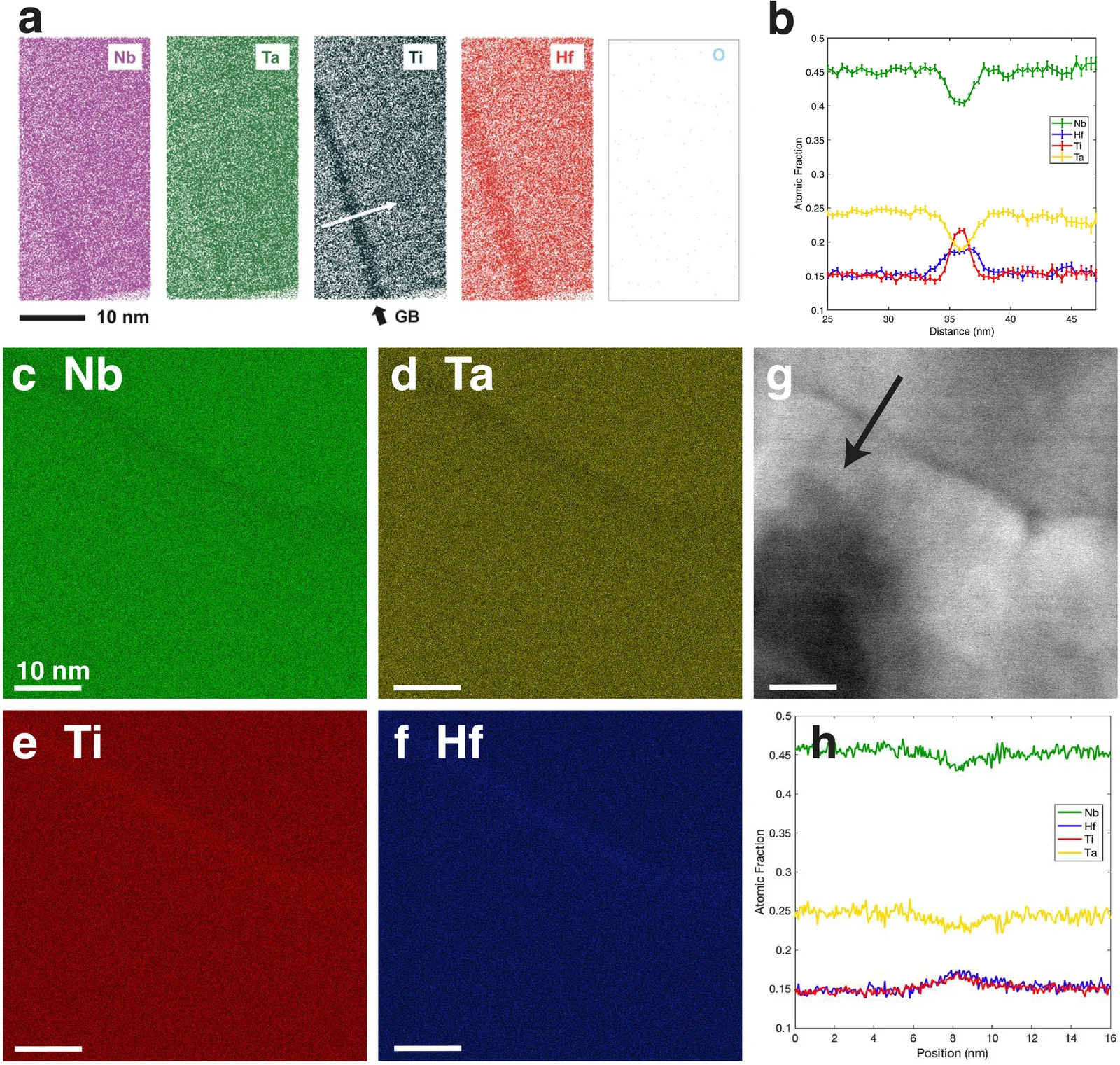
Grain-boundary chemistry of Nb_45_Ta_25_Ti_15_Hf_15_. (**a**) Atom probe tomography (APT) of a grain boundary in this alloy shows an enrichment of group IV elements in a ~ 4 nm range around the grain boundary. An alloy composition plot in (**b**) shows that the Ti and Hf concentrations are ~42 at.%, compared with their background of 30 at.%. Additional EDS maps of a region where twins and omega impact a grain boundary (from Fig. 3) are shown in element maps in (**c**)**-** (**f**) and in an HAADF-STEM overview image in (**g**). A line profile composition shown in (**g**) is plotted in (**h**) and shows a ~ 4 nm range with slight enrichment of group IV elements and a depletion of group V elements. Source data

These observations illustrate that lowering glide stresses enables global activation of dislocation glide at a crack tip, but it is not necessarily the critical mechanism preventing brittle fracture because the toughness of Nb_45_Ta_25_Ti_15_Hf_15_ is still nearly 100 MPa√m. These experiments indicate that chemical complexity raises the glide stresses, which elevates high-temperature strength, albeit with a modest cost to toughness, but that it is not a variable involved in determining the slip-twinning transition or DBTT^17^. We deduce that it is the combination of low twinning migration stresses and, most critically, low barriers to heterogeneous dislocation nucleation that is primarily a function of the electronic structure. Our observation in two tensile stress states is that twin grain-boundary sites nucleate dislocations instead of cracks, which (1) enables the generation of a plastic zone by twinning, and (2) suggests that dislocation nucleation (not glide) is a primary driver of the DBTT. Although dislocation nucleation stresses are generally very high and often difficult to compute, density functional theory studies show that adding group IV elements reduces the unstable stacking-fault energy of group V metals and alloys, a proxy for dislocation nucleation barriers^11^. These elements concomitantly promote twinning and the ω-phase transformation^23,39^. However, by promoting the transformation to the ω-phase, a phase predicted by the Rice parameter to be brittle, the group IV elements become a ‘double-edged sword^40^’. This transformation is especially problematic for samples containing cracks because high hydrostatic stress promotes stress-controlled transformations and exploits the new phase’s brittleness to accelerate fracture^41,42^. Density functional theory calculations at 0 K reveal that in NbTaTiHf alloys, increasing group IV concentrations promote lattice instabilities from bcc to ω-phase (ref. ^39^). At 30 at% Ti and Hf, the bcc phase in Nb_45_Ta_25_Ti_15_Hf_15_ is still relatively stable^39^. But the composition is heterogeneous. Atom probe tomography analysis of grain boundaries in Extended Data Fig. 9a,b shows that Ti and Hf enrich the boundaries in an approximately 3 nm range, raising the group IV concentration to 42 at% in the measured region. A TEM energy-dispersive X-ray analysis (Extended Data Fig. 9c–h) of the grain boundary from Fig. 4 at the twin/ω-phase impact site indicates that this boundary is also enriched in Ti and Hf. This produces a local environment that promotes dislocation nucleation, but it also produces an additional chemical driving force for stress-induced ω-phase formation^43^.

## Conclusion

Our findings demonstrate that NbTi-based alloys can retain exceptional damage tolerance, with no measurable DBTT observed down to 20 K. These properties are derived from a global mechanistic sequence that is unusual for bcc alloys in tension, which are often susceptible to high-temperature DBTTs^4^. Whereas most metallic alloys (particularly those with the fcc structure) generate high toughness from a global sequence that begins with dislocation glide and progresses to twinning and a phase transformation that delocalizes dislocation-induced damage^15,17^, the global sequence in the current alloys begins with twinning (mediated by screw dislocation core transformation) coupled to a phase transformation that nucleates dislocations to dissipate stress concentrations produced by the former two mechanisms. Dislocation glide subsequently follows. This achieves an unconventional pathway to inhibit a DBTT: crack-tip blunting is initiated by twinning, a near-temperature-insensitive mechanism that prevents a crack-tip plastic zone from shrinking and serves as an efficient dislocation source. Our results illustrate another pathway, independent of chemical complexity, for inhibiting the DBTT in refractory bcc alloys.

## Methods

### Alloy design

The Nb_45_Ta_25_Ti_15_Hf_15_ alloy was initially designed to balance room-temperature ductility and high-temperature strength, according to ref. ^17^. In summary, the original rationale for the chosen composition was to introduce a high concentration of group IV elements to promote ductility, but not too much as to promote the formation of brittle hexagonal close-packed and ω-phases. We did not include Zr because of its tendency to act as a secondary phase former^44^. The Nb/Ta ratio was chosen to promote lattice distortion while simultaneously minimizing the concentration of Ta because of its high density.

Although these studies were useful for the high-temperature applications of these alloys, they gave little indication of the low-temperature behaviour. Density functional theory and molecular dynamics simulations using a machine-learning atomic potential for this alloy indicated that the screw dislocation glide stresses had well exceeded the measured yield strength at 77 K (ref. ^38^). Moreover, our experimental observations at 77 K suggested that the slip-twinning transition had already occurred and that the yield strength was controlled by twinning rather than dislocation glide^17^. The combination of experimental observations and simulations motivated the evaluation of the mechanical behaviour of this alloy at even lower temperatures to assess the role of this mechanistic transition on the DBTT.

The composition of the Nb_70_Ti_30_ alloy was chosen to maintain the valence-electron count of Nb_45_Ta_25_Ti_15_Hf_15_ while reducing the chemical complexity to determine whether this variable is important for suppressing the DBTT. Tensile yield strength values in ref. ^31^ suggested that this transition could possibly occur in this alloy as well. To further motivate investigating this material, we calculated screw dislocation glide stresses considering a kink width of ten Burgers vectors and the same methodology and interaction energies given in ref. ^40^. We found that the screw glide stresses were slightly lower than those for the multi-principal element alloy: 930 MPa and 1,080 MPa at 77 K and 20 K, respectively. The edge dislocation glide stresses calculated using the Maresca–Curtin model at 77 K and 20 K are 75 MPa and 105 MPa, respectively. This provided an opportunity to assess the role of chemical complexity on the DBTT and indirectly evaluate whether the operation of Frank–Reed sources controls the DBTT^45^. Our results suggest that electronic structure is the primary variable controlling this mechanistic transition and the DBTT, and that the relative velocity of screw and edge dislocations does not inhibit dislocation multiplication and glide. This latter point can also be rationalized by the rarity of Frank–Reed sources in single-phase materials.

### Strain hardening

The absence of notable strain hardening in tension is also of particular interest, as this property is often associated with high toughness in TWIP and TRIP alloys, exemplified by CrCoNi (ref. ^15^). In these fcc systems, twinning and a phase transformation follow dislocation glide to elevate strain hardening and, in turn, ductility and toughness^46,47^. Conversely, in the bcc systems, activation of twinning before dislocation glide arises from an intrinsic competition between dislocation motion and dissociation into a twin embryo^22^. This process instantly transforms grains into ‘multi-lane highways’ of varying Schmidt factor (Extended Data Fig. 5b,c). As the applied stress increases, glide is progressively favoured over twinning, with approximately 50% of the grains in the anti-twinning orientation expected to deform by screw dislocation glide with the original compact core. Dislocations with the lowest slip barriers are selected—specifically those that can cross-slip along twins—channelling them along these boundaries. The ω-phase, as a twin transitional structure, can further promote channelling by preventing dislocation transmission across twin boundaries. In regions where the ω-phase spans the distance between adjacent twins (Fig. 3b), dislocations can accumulate, similar to materials with pre-existing ω-phase particles^48^. Although this hampers uniform ductility, it assists sustained necking, leaving the total fracture strain high and nominally elevating toughness^17^.

### Role of microstructure on the DBTT

Here we address three important microstructural variables that can impact the DBTT, and the resulting limitations this imposes on this study. First, the DBTT is strongly dependent on the concentration of interstitials in all bcc alloys. Studies on group IV–V multi-principal element alloys suggest that oxygen can have a particularly pronounced impact on strength and ductility^9^. Although multiple experiments illustrate that the addition of this interstitial increases room-temperature ductility by promoting strain hardening, it can simultaneously raise the DBTT to above 77 K (refs. ^9,49^). It is also known to raise twinning and phase transformation stresses, thereby suppressing these mechanisms^21,40^. However, this interplay is highly nonlinear, that is, O interstitials suppress the ω-phase transformation more relative to twinning^21^. Further work to understand the role in suppressing the ω-phase on twin embryo growth and propagation is needed. The relatively low interstitial concentration in Nb_45_Ta_25_Ti_15_Hf_15_ and Nb_70_Ti_30_ suppresses additional pinning stresses that would otherwise inhibit dislocation glide and twin migration. We postulate that additions of O to the material, probably above 1 at%, would raise the DBTT to above 20 K (refs. ^9,50^).

Grain size can also affect the DBTT of bcc alloys by raising both dislocation and twinning stresses through the Hall–Petch effect. Coarse grain sizes can be beneficial for increasing ductility and toughness by promoting dislocation glide. However, this only works up to a certain point. In fact, toughness and ductility begin to decrease with grain coarsening past a certain size owing to the promotion of strain localization^51^. Moreover, experimental work on multiple alloys indicates that materials undergo the slip-twinning transition across a large temperature range, and that increases in grain size can promote twin propagation, but potentially suppress twin boundary migration^28,52^. In Fe–Si, increasing grain size promotes embrittlement through this mechanism^28^. It is found that, like dislocations, twins obey a Hall–Petch relationship^21^. Further experimental work is needed to assess the Hall–Petch constants.

Finally, recrystallization has been shown to influence the DBTT. This effect is most pronounced in intrinsically brittle materials such as chromium and tungsten, in which extensive hot rolling can introduce high dislocation densities. The presence of these dislocations prevents the need to nucleate them at low temperatures, and owing to the inherent presence of kink pairs, these dislocations can more easily glide at low temperatures. As a result, materials such as tungsten and chromium can have their DBTTs suppressed by several hundred kelvin simply by introducing defects that are not entirely removed during recrystallization^53,54^. This effect also increases the cryogenic ductility of Nb and its alloys, but in refractory multi-principal element alloys it can substantially raise the yield stress owing to forest hardening, as is the case in Nb_40_Ta_25_Ti_15_Hf_15_Zr_5_ (refs. ^55,56^). When this alloy is not fully recrystallized, the room-temperature yield strength increases by nearly 100 MPa, reflecting limited dislocation glide that would compound at lower temperatures. This effect has been documented in another similar alloy^57^. As a result, partial recrystallization is beneficial by providing the material with already mobile dislocations, but it also introduces additional pinning sites for dislocations, thereby raising the strength and constricting plastic deformation.

### Materials processing and microstructural characterization

The Nb_45_Ta_25_Ti_15_Hf_15_ was produced from high-purity elements (>99.9%), which were arc-melted and then flipped and remelted several times to ensure chemical homogeneity. The resulting 12-mm-thick ingots were forged and then rolled to approximately 8 mm (corresponding to a 33% reduction in thickness), and then heat-treated at 1,100 °C in air in sealed stainless-steel bags for 1 hour, followed by water quenching. This process was identical to that in ref. ^17^. The microstructure produced consisted of near-fully recrystallized grains approximately 259 μm in size.

The Nb_70_Ti_30_ was produced from high-purity elements (>99.9%) that were arc-melted, then flipped and remelted several times to ensure chemical homogeneity. The resulting 10-mm-thick ingots were then cross-rolled to approximately 6.7 mm (corresponding to a 33% reduction in thickness) and heat-treated at 1,077 °C in air in sealed stainless-steel bags for 1 hour, followed by water quenching. This heat-treatment temperature was chosen to be the same fraction of the solidus temperature (approximately 0.56) as Nb_45_Ta_25_Ti_15_Hf_15_. The microstructure consisted of a bimodal grain distribution. Some grains were fully recrystallized and averaged approximately 51 μm, whereas others were partially recrystallized with an average diameter of approximately 412 μm. These grains were further subdivided by low-angle boundaries consisting of recrystallized rotational twins and kink bands.

To analyse the microstructure of the material, the samples were cut perpendicular to the rolling direction, embedded in resin and mechanically polished to a surface finish of 0.04 μm. The sample was then analysed by EBSD in a Thermo Fisher Scios 2 Dual Beam scanning electron microscope (SEM) at an accelerating voltage of 20 kV. Bulk O and N interstitial concentrations were analysed by inert gas fusion and carbon by combustion.

### Mechanical characterization

To evaluate the tensile properties, three rectangular dog-bone specimens with a gauge length of 12 mm were electron-discharge machined from the ingots. All sides of the specimen were polished to 1000 grit SiC paper to a final cross-sectional area of 3.13 mm^2^. Tensile tests were performed under quasi-static conditions with a strain rate of 10^−3^ s^−1^. The strain was measured with a 10-mm 3442 clip-on extensometer (Epsilon Technology) rated to perform at temperatures as low as approximately 1 K.

The yield strength, *σ*_y_, ultimate tensile strength, *σ*_u_, uniform ductility, *ε*, and elongation to fracture, *ε*_f_, were determined from the uniaxial engineering stress–strain curves. Corresponding true stress–strain (*σ*^T^/*ε*^T^) curves were calculated and then compared with instantaneous strain-hardening curves (d*σ*⁄d*ϵ*) as a function of strain to evaluate the hardening behaviour. A strain-hardening exponent was additionally evaluated using the constitutive power-hardening law *σ*^T^ = /*k*(*ε*^T^)^*n*^. Here *n* is the strain-hardening exponent and *k* is a scaling constant. However, the material did not exhibit ideal hardening behaviour, and consequently, *n* represents an average hardening value.

To evaluate the fracture properties, two compact-tension specimens were cut from the ingots by electron-discharge machining with nominal width *W* = 16 mm and thickness *B* = 3.5 mm. To prepare the samples for fracture by overload, an atomically sharp crack was inserted into the sample by fatigue pre-cracking. First, both faces were mechanically polished to a final finish of 0.04 μm to allow for crack-length measurement with a long-distance optical microscope. The machined notch, which was approximately 3.5 mm in length, was then micro-notched with a razor blade coated in a 6-μm diamond paste to a final notch root radius *ρ* ≈ 10 μm. Finally, samples were cyclically loaded in a 4.5-kN servo-hydraulic 810 MTS load frame operated using an Instron 8800 digital controller using load control under a constant-amplitude sinusoidal wave of load ratio *R* = 0.1 (where *R* is the ratio of the minimum to maximum load) at a frequency of 15 Hz. To ensure that the plastic zone around the pre-crack tip did not affect the fracture tests, the stress intensity was gradually reduced such that the stress intensity range, Δ*K*, was 10–12 MPa√m at a final pre-crack length in the range of *a*/*W* ≈ 0.6. Specimens were then side-grooved to a final thickness *B*_N_ ≈ 3 mm to ensure the that crack followed the mode I loading path.

During the fracture test, the load-line displacement was measured using a 3-mm gauge length Epsilon 3541 crack opening displacement gauge gauge rated to perform at temperatures as low as approximately 1 K. To measure the crack length, the sample was systematically unloaded by approximately 15% of the peak load to measure the elastic unloading compliance. Crack lengths, *a*_i_, were calculated from equations provided by ASTM Standard E1820 for compact-tension samples where the compliance is measured on the load-line^30^:

1 $${a}_{{\rm{i}}}/W=1.000196-4.06319u+11.242{u}^{2}-106.043{u}^{3}+464.335{u}^{4}-650.677{u}^{5}$$

where

2 $$u=\frac{1}{\sqrt{{B}_{{\rm{e}}}E{C}_{{\rm{C}}({\rm{i}})}}+1},$$

*B*_e_ is the effective thickness (${B}_{{\rm{e}}}=B-{(B-{B}_{{\rm{N}}})}^{2}/B$), *E* is the elastic modulus, and *C*_C(i)_ is the rotation corrected compliance. Next, for each crack length, *a*_i_, the *J* integral was calculated as a sum of the elastic, *J*_el_, and plastic, *J*_pl_, strain energy contributions:

3 $${J}_{({\rm{i}})}={J}_{\mathrm{el}({\rm{i}})}+{J}_{\mathrm{pl}({\rm{i}})},$$

with ${J}_{\mathrm{el}({\rm{i}})}=\frac{{K}_{{\rm{i}}}^{2}}{{E}^{{\prime} }}$ where ${E}^{{\prime} }=\frac{E}{1-{\upsilon }^{2}}$ is the elastic modulus in plane-strain conditions (*ν* is Poisson’s ratio) and *K*_i_ is the linear-elastic stress intensity calculated at each corresponding load–displacement point for compact-tension geometry. Finally, the plastic component of *J* integral, *J*_pl(i)_ is derived from the Merkle–Corten analysis, which yields the following expression^58^:

4 $${J}_{\mathrm{pl}({\rm{i}})}=\left[{J}_{\mathrm{pl}({\rm{i}}-1)}+\left(\frac{{\eta }_{\mathrm{pl}({\rm{i}}-1)}}{{b}_{({\rm{i}}-1)}}\right)\left(\frac{{A}_{\mathrm{pl}({\rm{i}})}-{A}_{\mathrm{pl}({\rm{i}}-1)}}{{B}_{{\rm{N}}}}\right)\right]\left[1-{\gamma }_{(i-1)}\left(\frac{{a}_{({\rm{i}})}-{a}_{({\rm{i}}-1)}}{{b}_{({\rm{i}}-1)}}\right)\right]$$

where ${\eta }_{\mathrm{pl}({\rm{i}}-1)}$ and ${\gamma }_{({\rm{i}}-1)}$ are functions of the uncracked ligament, ${b}_{({\rm{i}}-1)}$, and ${A}_{\mathrm{pl}({\rm{i}})}-{A}_{\mathrm{pl}({\rm{i}}-1)}$ is the area under the plastic contribution of the load–displacement curve:

5 $${\gamma }_{(i-1)}=1+0.76{b}_{(i-1)}/W$$

6 $${\eta }_{{pl}(i-1)}=2+0.522{b}_{(i-1)}/W$$

With this formulation, the value of the *J* integral at each instantaneous loading–unloading sequence, *J*_i_, can be calculated and related to the corresponding crack length. The resulting *J*–Δ*a* curve can be used to calculate (1) a provisional crack-initiation toughness, *J*_Q_, by finding its intersection with the 0.2-mm blunting line whose slope is two times the flow stress, 2*σ*_f_; and (2) a crack-growth toughness that is evaluated at the maximum valid crack extension of Δ*a* = 0.25*b*_0_, where *b*_0_ is the initial uncracked ligament. To determine a valid, size-independent fracture toughness, specimens must satisfy plane-strain and *J*-dominance conditions for validity given by ${b}_{0},B > \frac{10{J}_{{\rm{Q}}}}{{\sigma }_{{\rm{f}}}}$. All specimens met these criteria in Nb_45_Ta_25_Ti_15_Hf_15_ but not all in Nb_70_Ti_30_. However, in all the latter cases, the specimens were just narrowly above validity, and we did not observe ‘concave-up’ *R*-curve behaviour associated with the transition to non-plane-strain conditions. We therefore believe that the values are valid as well.

To equate these values to the stress intensity, the elastic properties (Young’s modulus and Poisson’s ratio) were calculated from measurements of the shear and longitudinal sound velocities using a 45MG ultrasonic thickness gauge (Evident Scientific). The following equations were then used to extrapolate Poisson’s ratio (*ν*) and Young’s modulus (*E*):

7 $$\upsilon =\frac{1-2{\left(\frac{{V}_{{\rm{T}}}}{{V}_{{\rm{L}}}}\right)}^{2}}{2-2{\left(\frac{{V}_{{\rm{T}}}}{{V}_{{\rm{L}}}}\right)}^{2}},$$

where *V*_L_ and *V*_T_ are the longitudinal and transverse velocities, respectively. From these three values, the Young’s modulus can then be calculated with:

8 $$E=\frac{{V}_{{\rm{L}}}^{2}{\rho }_{{\rm{d}}}(1+\upsilon )(1-2\upsilon )}{1-v},$$

where *ρ*_d_ is the density measured by the Archimedes method and determined to be 10.8 g cm^−3^ for Nb_45_Ta_25_Ti_15_Hf_15_ and 7.4 g cm^−3^ for Nb_70_Ti_30_. The values for the modulus at 77 K of Nb_45_Ta_25_Ti_15_Hf_15_ were obtained from a previous study, *E* = 121.8 GPa and *v* = 0.37, using values obtained from tensile tests, normalized to the ultrasonic measurements. For Nb_70_Ti_30_, the elastic properties were obtained at room temperature to be *E *= 100.8 GPa and *v* = 0.386. We used these values across the entire temperature regime, assuming negligible thermal softening.

### Mechanical testing set-up

For the specific purposes of this study, uniaxial tensile and fracture toughness data were obtained with samples cooled with gaseous helium to a temperature of 20 K. Corresponding results for Nb_45_Ta_25_Ti_15_Hf_15_ at temperatures between 77 K and 298 K, based on similar testing procedures described below, are available in ref. ^17^.

For the present study, the tensile and fracture toughness tests were performed at the ENGIN-X beamline at the ISIS Neutron and Muon Source at the Rutherford Appleton Laboratory at Harwell Campus, Oxfordshire, in the UK. The details of this set-up are described in ref. ^15^. In brief, it consists of an Instron 100-kN rated hydraulic load frame with an Instron 8800 Controller attached to a vacuum-sealed chamber. Two cryo-coolers are connected to the grips with a copper clamp and a series of laminated copper sheets to ensure optimal thermal conductivity. A set of grips and pins made from a high-strength and thermally conductive CuBe alloy were installed into the cold fingers. To further ensure that the temperature reached 20 K, thermally conductive copper grease was generally placed in all connective joints. The temperature was measured with two rhodium–-iron thermocouples attached to the grips directly adjacent to the samples. Owing to the small sample size, it was not possible to attach a thermocouple to the samples without direct interference with the strain field around the crack. To verify that samples reached 20 K, an additional, significantly larger stainless-steel compact-tension sample with a larger thermal mass (*W* = 18 mm, *B* = 9 mm) was placed in the grips with one of the thermocouples embedded in a copper foil attached to the backside of the sample. Temperatures in the grips reached 8–15 K, whereas those at the edge of sample reached 28–35 K. We approximate the crack-tip temperature as an average between the two: 18–25 K, which for simplicity we state as 20 K.

### Post situ characterization of deformation mechanisms

After testing, the broken samples were immersed in acetone to prevent oxidation. The remaining uncracked ligament in one compact-tension sample was fractured by fatigue; the other compact-tension sample was cut by electron-discharge machining at the mid-thickness (*B*/2). Fractographic analysis was performed on the fractured sample using a Thermo Fisher Scios 2 Dual Beam SEM at an accelerating voltage of 10 kV using secondary electrons. The sample sectioned at mid-thickness was polished to a final finish of 0.04 μm and imaged in the SEM at an accelerating voltage of 20 kV by EBSD with a step size range of 0.2–1.25 μm. Analysis of EBSD data was performed using Aztec Crystal, a commercially available software.

To understand the grain-boundary chemistry, two sharp needle-like specimens of (1) a fracturing grain boundary and (2) a grain boundary of a grain that did not undergo intergranular fracture were prepared by the focused-ion-beam lift-out and annular milling techniques using a FEI Helios 5 Hydra UX. Three-dimensional atom probe analysis was performed using a local electrode atom probe (CAMECA LEAP 6000 XR) in laser pulse mode with a pulse energy of 60 pJ at a temperature of 70 K.

STEM was carried out to characterize the defect structures in the vicinity of the crack. Site-specific TEM foil samples were cut from the fractured samples and thinned to electron transparency using focused-ion-beam milling (FEI, Helios G4 UX and FEI, Scios 2). Two TEM lamellas were trenched and lifted out from the fracture surface and across grain boundaries near the crack tip from compact-tension test specimens. TEM lamella preparation finished with a 5 kV followed by a 2 kV Ga^+^ polish. STEM, TEM, HRTEM and 4D-STEM imaging of samples were conducted on FEI TitanX and FEI ThemIS microscopes (operated at 300 kV) at the National Center for Electron Microscopy (NCEM, LBNL). Convergence angles of 0.48 mrad to 1.8 mrad were used with an electron probe size of approximately 1.0 nm to 2.3 nm and a scanning step size of 1.085 nm to 8.61 nm. 4D-STEM data were analysed using the py4DSTEM software package^59^. Samples were then examined in the SEM at an accelerating voltage of 30 kV by TKD with a step size of 10 nm. Samples used to characterize tensile deformation analysis were prepared from tensile samples that were interrupted post-yielding and at uniform ductility by a conventional twin-jet polishing technique, using a solution of 10% H_2_SO_4_ and 90% CH_4_O solution at −45 °C with an applied voltage of 40 V.

### Crack-tip stress–strain state analysis

To quantify the stress–strain state at the crack tip, the nonlinear elastic HRR plastic strain field was calculated as a function of the *J* integral, radial distance, *r*, and angle, *θ*. To perform this calculation, the material was taken to have a power-hardening law. All stresses and strains are true stresses and strains (for simplicity we have removed the ‘T’ superscript):

9 $${\bar{\varepsilon }}_{{\rm{p}}}/{\varepsilon }_{{\rm{y}}}={\alpha (\bar{\sigma }/{\sigma }_{{\rm{y}}})}^{N},$$

where *ε*_y_ and *σ*_y_ are the yield strain and stress, *α* is a unitless constant (of order unity) and *N* is the strain-hardening exponent (where 1 ≤ *N* ≤ ∞). For this alloy at 20 K, *N* ≈ 10. It is noted that this equation is the inverse of the preceding power-hardening law. Given these material constants, the HRR field can be calculated from the following expressions:

10 $${\varepsilon }_{{ij}}={\alpha \varepsilon }_{{\rm{y}}}{\left[\frac{J}{{\alpha \varepsilon }_{{\rm{y}}}{\sigma }_{{\rm{y}}}{I}_{{\rm{n}}}r}\right]}^{\frac{N}{N+1}}\widetilde{{\varepsilon }_{{ij}}}(\theta ,N),$$

where *I*_n_ is an integration constant that is a function of the strain-hardening exponent *N*, and $\widetilde{{\varepsilon }_{{ij}}}(\theta ,N)$ is a dimensionless function of strain describing the variation of each strain component as a function of *θ* and *N*. The stress can be similarly calculated:

11 $${\sigma }_{{ij}}={\sigma }_{y}{\left[\frac{J}{{\alpha \varepsilon }_{{\rm{y}}}{\sigma }_{{\rm{y}}}{I}_{{\rm{n}}}r}\right]}^{\frac{1}{N+1}}\widetilde{{\sigma }_{{ij}}}(\theta ,N),$$

where similarly, $\widetilde{{\sigma }_{{ij}}}(\theta ,N)$, is a dimensionless function of stress describing the variations of each stress component as a function of *θ* and *N*.

Given that the sample in Fig. 3 was taken 30 μm ahead of the crack-tip (*θ* = 0°) under plane-strain conditions, the strain tensor, converted to Cartesian coordinates is given as:

$${\varepsilon }_{{ij}}=\left[\begin{array}{ccc}-0.741 & 0 & 0\\ 0 & 0.741 & 0\\ 0 & 0 & 0\end{array}\right]\times {10}^{-2},$$

with the corresponding stress tensor (in MPa) as:

$${\sigma }_{{ij}}=\left[\begin{array}{ccc}2,677.0 & 0 & 0\\ 0 & 3,876.4 & 0\\ 0 & 0 & 3,276.7\end{array}\right].$$

where ${\sigma }_{33}=v({\sigma }_{11}+{\sigma }_{22})$ and *v* ≈ 1/2 during plastic deformation. In this region, crack-tip stresses are approximately three-times higher, and the strains are smaller than those reached in uniaxial tensile samples.

### Micromechanical models of stress-controlled fracture for an inverted mechanistic sequence

We constructed a micromechanical model to predict the fracture toughness, *K*_JIc_, of bcc alloys that undergo a sequence of mechanisms that begins with twinning. Our model assumes that dislocation glide is only activated by twinning and that the screw glide stresses are significantly higher than the twinning stresses, such that plasticity is solely controlled by twinning and subsequent edge glide. We postulate that semi-brittle intergranular cleavage occurs when the local stress calculated by the HRR field exceeds the upper-bound twinning stress. We first consider the largest difference between the principal stress calculated from the HRR field directly in front of the crack tip, that is, when *θ* = 0°, to consider effects from constraint.

$$\Delta {\sigma }_{\max }={\sigma }_{\theta \theta }{-\sigma }_{{rr}}={\sigma }_{{yy}}{-\sigma }_{{xx}}(\theta =0),$$

$${\Delta {\sigma }_{\max }=\sigma }_{{yy}}{-\sigma }_{{xx}}={\sigma }_{y}{\left[\frac{J}{{\alpha \varepsilon }_{y}{\sigma }_{y}{I}_{n}r}\right]}^{\frac{1}{N+1}}\widetilde{{\sigma }_{\theta \theta }}(0,N)-{\sigma }_{y}{\left[\frac{J}{{\alpha \varepsilon }_{y}{\sigma }_{y}{I}_{n}r}\right]}^{\frac{1}{N+1}}\widetilde{{\sigma }_{{rr}}}(0,N),$$

$${\Delta {\sigma }_{\max }=\sigma }_{{\rm{y}}}{\left[\frac{J}{{\alpha \varepsilon }_{{\rm{y}}}{\sigma }_{{\rm{y}}}{I}_{{\rm{n}}}r}\right]}^{\frac{1}{N+1}}[\widetilde{{\sigma }_{\theta \theta }}(0,N)-\widetilde{{\sigma }_{{rr}}}(0,N)].$$

On the basis of our postulate, fracture occurs when Δ*σ*_max_ exceeds the twinning stress at a critical microstructural length scale. Given that the upper-bound twinning stress is the yield strength, fracture occurs when Δ*σ*_max_ =* σ*_y_ at *r* = *md*_g_, where *m* is an integer multiple of the grain-boundary diameter, *d*_g_.

$${{\sigma }_{{\rm{y}}}=\sigma }_{{\rm{y}}}{\left[\frac{{J}_{\mathrm{Ic}}}{{\alpha \varepsilon }_{{\rm{y}}}{\sigma }_{{\rm{y}}}{I}_{{\rm{n}}}m{d}_{{\rm{g}}}}\right]}^{\frac{1}{N+1}}[\widetilde{{\sigma }_{\theta \theta }}(0,N)-\widetilde{{\sigma }_{{rr}}}(0,N)].$$

Re-arranging gives:

$${J}_{\mathrm{Ic}}={\alpha \varepsilon }_{{\rm{y}}}{\sigma }_{{\rm{y}}}{I}_{{\rm{n}}}m{d}_{{\rm{g}}}{[\widetilde{{\sigma }_{\theta \theta }}(0,N)-\widetilde{{\sigma }_{{rr}}}(0,N)]}^{-(N+1)}.$$

Simplifying by substituting *ε*_y_ = *σ*_y_/*E*, and converting to the critical stress intensity, that is, ${K}_{\mathrm{JIc}}=\sqrt{{J}_{\mathrm{Ic}}E/1-{v}^{2}}$ gives the following expression:

$${K}_{\mathrm{JIc}}={\sigma }_{{\rm{y}}}\sqrt{{{md}}_{{\rm{g}}}\frac{\alpha {I}_{{\rm{n}}}}{1-{v}^{2}}{[\widetilde{{\sigma }_{\theta \theta }}(0,N)-\widetilde{{\sigma }_{{rr}}}(0,N)]}^{-(N+1)}},$$

to which we assign the factor:

$$\delta (N)=\sqrt{\frac{\alpha {I}_{{\rm{n}}}}{1-{v}^{2}}{[\widetilde{{\sigma }_{\theta \theta }}(0,N)-\widetilde{{\sigma }_{{rr}}}(0,N)]}^{-(N+1)}}.$$

Which leads to the final expression:

$${K}_{\mathrm{JIc}}={\delta (N)\sigma }_{{\rm{y}}}\sqrt{{{md}}_{{\rm{g}}}}.$$

All these values can easily be obtained from a tensile test. Approximations can be made; however, we caution that the stress intensity is sensitive to small changes in *J*_Ic_ values when they are low.

### Measurements of the DBTT

The DBTT can be assessed with multiple measurements. Typically, the mechanical properties of a material at the DBTT are characterized by an increase in strength, cessation in tensile ductility and/or a decrease in fracture toughness. It can also be measured by an examination of the fracture mode with the change from ductile fracture (for example, microvoid coalescence) to the onset of transgranular cleavage and/or intergranular fracture, thus marking the transition. Although the NbTi alloys show a reduction in tensile ductility and toughness from room temperature, and a transition to a nominally intergranular fracture, the materials still have toughness of between approximately 100 MPa√m and 170 MPa√m, and tensile fracture strains of approximately 14%. The presence of significant plasticity ahead of the crack tip additionally indicates that the material does not exhibit brittle fracture behaviour and thus shows no DBTT.

## Online content

Any methods, additional references, Nature Portfolio reporting summaries, source data, extended data, supplementary information, acknowledgements, peer review information; details of author contributions and competing interests; and statements of data and code availability are available at https://doi.org/10.1038/s41586-026-11065-7.

## Supplementary information

Supplementary Figures Supplementary Figs. 1–6.

Peer Review File

## Source data

Source Data Fig. 1

Source Data Fig. 3

Source Data Fig. 4

Source Data Extended Data Fig. 2

Source Data Extended Data Fig. 7

Source Data Extended Data Fig. 9

## Figures and Tables

**Extended Data Table 1:**
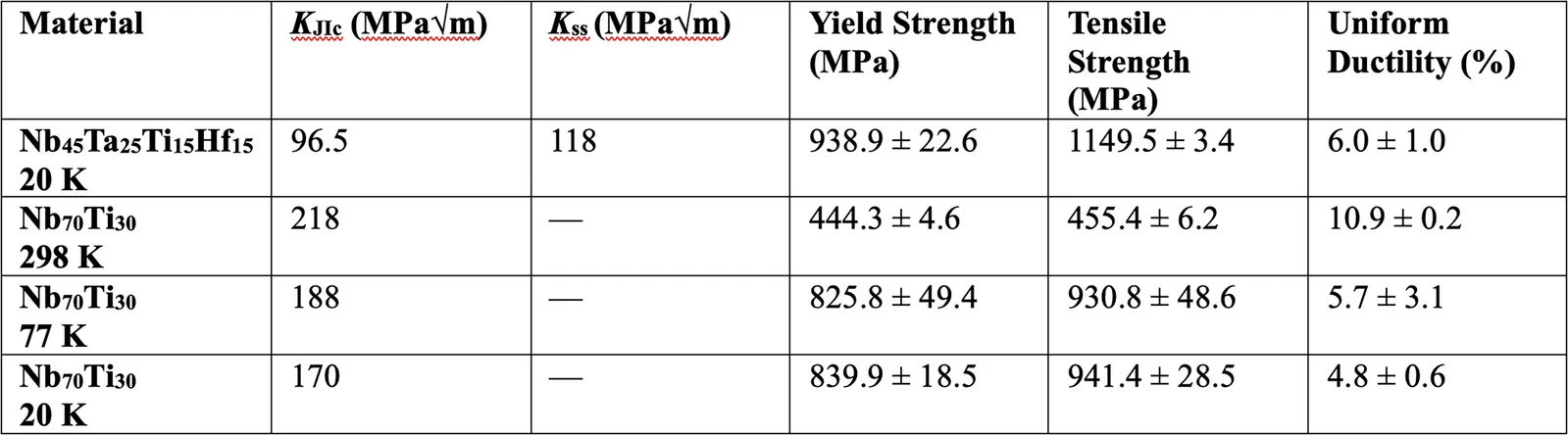
Summary of mechanical properties of Nb_45_Ta_25_Ti_15_Hf_15_ and Nb_70_Ti_30_

Data are collected from three tensile tests and two fracture tests per temperature.

## Data Availability

All data that support the findings of this study are reported in the main paper, extended data and supplementary materials. Source data are provided with this paper.
