# Supplementary material for: Competing mechanisms toughen bcc binary and medium-entropy alloys at 20 K
Source: Nature. 2026 Oct 14;658(8137):923–9. doi: 10.1038/s41586-026-11065-7 (PMC13686945; doi:10.1038/s41586-026-11065-7)
Supplement: Supplementary file 1 [file 41586_2026_11065_MOESM2_ESM.pdf]

nature portfolio

Peer Review File

Competing mechanisms ductilize bcc binary and medium-entropy alloys at 20 K

Corresponding Author: Professor Robert Ritchie

Any redactions in this file are there to maintain patient confidentiality, the confidentiality of unpublished data, or to remove third-party material.

This file contains all reviewer reports in order by version, followed by all author rebuttals in order by version.

Version 0:

Reviewer comments:

Referee #1

(Remarks to the Author)
A Summary of the key results

Body centered cubic (BCC) metals and alloys exhibit characteristic transitions from low to high ductility/toughness when surpassing a certain temperature – the brittle-to-ductile transition temperature (BDTT). This type of embrittlement obtained at low temperatures is often limiting the engineering application scenarios of otherwise interesting candidate materials.
In the present manuscript, the authors provide a comprehensive analysis of the low temperature mechanical performance, incl. ductility and toughness, of a concentrated solid solution, i.e. $Nb_{45}Ta_{25}Ti_{15}Hf_{15}$ formerly introduced in https://doi.org/10.1126/science.adn2428. The data impressively shows that the alloy in the microstructural condition investigated exhibits reasonable ductility (not only strain to fracture but also uniform strain!) as well as toughness even at 20 K. For most application scenarios, no relevant BDTT is thus obtained. It is specifically noteworthy that this is the case for both assessment types, i.e. ductility and toughness, which is not often provided at the same time in literature. The microstructural and theoretical assessment of the finding reveals that an unusual combination of deformation mechanisms contributes to this behavior. In the present case, deformation twinning and phase transformation mediated plasticity prevent a temperature-dependent increase of strength at low temperatures. This behavior mitigates a strong constriction of the plastic zone at the crack tip.

B Originality and significance, C Data & methodology, D Appropriate use of statistics and treatment of uncertainties, E Conclusions

The findings are surprising and interesting to the field of physical metallurgy, the quality of the provided data is high, the assessment sound and statistically relevant. Thus, I recommend the manuscript for publication after addressing my comments provided in the following.

F Suggested improvements

In contrast to other publications, the authors provide data on the contamination levels. The authors might append the information that the ppm are indeed weight-ppm. The reported contamination levels are comparably low which is important for the assessment of phase stability and transformation induced plasticity. Thus, I recommend including an assessment of the data for the reader with reference to literature.
The material under investigation is stated to be “near-fully” recrystallized. Indeed, the electron backscatter diffraction (EBSD) data in the Extended Data reveals substantial deformation being left in the microstructure. (i) With respect to the presentation of the EBSD data, the pattern quality maps should always be provided separated to allow for the assessment of localized features (twinning/transformation/kink banding/etc.). (ii) For the assessment of the recrystallization progress, EBSD data on the rolled condition should be provided for proper assessment; 1000 °C/1 h are indeed low/short while the grain size is comparably coarse for static recrystallization of a concentrated solid solution. Thus, proof of any kind of recrystallization (as opposed to recovery) is required. (iii) The impact of remaining dislocations on the obtained behavior remains elusive – however, proper pre-deformation was previously proven to substantially affect ductility and BDTT of refractory elements.

The atmosphere in the stainless-steel container during the heat treatment should be mentioned.
I recommend including the strain hardening curves into Fig. 1a to visualize the ability of the material to uniformly deform. This is unusual for BCC metals and alloys at such low temperatures and an important finding.
The microvoids indicated in Fig. 1e are not to be identified by the reader. The micrographs should be revised to properly prove the statement.
The authors provide evidence that the sequence of deformation mechanisms is changing as a function of temperature, specifically from 77 to 20 K. However, the strain hardening does not change. This needs to be discussed.
With respect to the common (and potentially not correct) interpretation of twinning/transformation induced plasticity as a dynamic microstructure refinement process, dislocation activity (to promote hardening by the additional boundaries) might be required to explain the strain hardening. This would not be the case if exclusively deformation twinning is active. A mechanistic discussion is required to explain why strain hardening remains the same for 77 and 20 K.
The authors state "It is not initially intuitive as to how twinning can cause intergranular fracture. After all, it is a form of plasticity." This needs further elaboration. In contrast to dislocation slip, deformation twinning will always result in a very confined shear strain accumulation within the surrounding material (depending on the thickness of the twin) at the twin "tip"/termination. This might particularly be relevant when twins approach grain boundaries. The localization aspect anyway comes a bit short in the discussion.
Based on the investigations, it is not possible to conclude whether twinning or transformation comes first. I agree with this. Hence, the terminology "omega wetting twin boundaries" is not suitable as it suggests the sequence of twins forming first and then omega on their boundaries.
Deformation twinning can exhibit rather pronounced size dependences in BCC metals and alloys. This might need to be considered when the interaction of twins with grain boundaries and cracks are concerned. This is currently not properly addressed in the manuscript (please see the suggestions on literature on refractory elements and their alloys).
The authors state "The inverted sequence, however, is critical to sidestepping deformation through dislocation glide, which would otherwise cause embrittlement." This is somewhat misleading. The dislocation mediated plasticity is strictly not the cause of embrittlement. With respect to ductility, it is the increasing yield strength and its competition to fracture stress that limits low temperature plastic deformation, not dislocation activity itself. With respect to cracks, a constriction of the plastic zone is certainly expected but this should be a continuous behavior and not causing a "transition". The statement requires more elaboration.
It is noted that the appearance of twinning or transformation comes along with stress drops. This is not further mentioned and outlined but needs to be discussed in further detail. What are the stress drops seen at any temperature in the stress-strain curves presented?
Localized deformation, i.e. by twinning/transformation, will cause local heating which is of particular importance during low temperature deformation as heat capacity and thermal conductivity are very low. Is there any temperature surveillance during the tests to proof the local temperature specifically when crack propagation is addressed?
I recommend appending the relevant mechanical data (yield strength, stress of first stress drop, ultimate tensile strength, uniform strain, strain to fracture, K_JIc, K_ss, etc.) to Table 1 to also provide quantitative insights to the performance of the alloy.

G References

Deformation twinning in BCC metals and alloys is still not properly understood, specifically when compared to face centered cubic metals and alloys. Though, there are plenty of investigations on refractory elements and concentrated alloys thereof found in literature, pure Nb, Ta, Cr-Fe, Nb-V, Mo-Re, MoWRe, etc. The proper assessment of these reports comes a bit short in the manuscript. With respect to ductility and the activation of deformation twinning in group 4/5 concentrated solid solutions, the authors might consider a recently published pre-print from another group, https://doi.org/10.21203/rs.3.rs-6422281/v1.

H Clarity and context

The title and abstract denote the present alloy as _medium_ entropy alloy. These are the only occasions when the term is introduced. In other instances, only refractory _high_ entropy alloys are named. In the current assessment, no evidence for the relevance of entropy is provided. While I understand that the naming might be relevant to attract attention, the basic mechanisms seem to be specific to solid solutions or concentrated solid solutions (of group 4/5 elements). The designation might therefore be adopted.
The authors frequently note twinning and transformation to be "overactivated". The terminology needs to be properly introduced and defined. While I can anticipate the background of the terminology, the use of such a concept has some restrictions with respect to a deformation mechanism which can never be "overactivated" from a plasticity point of view.

Referee #2

(Remarks to the Author)
This study discusses the mechanism by which $Nb_{45}Ta_{25}Ti_{15}Hf_{15}$ alloys produce a unique deformation mode called slip-twinning transition, which mitigates the sudden increase in yield strength due to temperature decrease and avoids DBTT caused by plastic deformation due to dislocations. We believe that this is an excellent discovery for an alloy that overcomes an intrinsic weakness. I believe it is basically worthy of publication, though I'm concerned that there is overlap with the discussion in Reference 20. I concur that this work is impactful and suitable for a prestigious publication like Nature. Nonetheless, I suggest some revisions to further refine the manuscript before recommending its acceptance.

pp.2: To evaluate how these mechanisms interact to affect this alloy's DBTT, we investigated its mechanical behavior in

tension at 20 K,…
pp.2: We observe that by passing through its slip-twinning transition, this alloy reverses the sequence of mechanisms occurring at higher temperatures: twins activate first, generate a plastic zone, and produce dislocation sources.
I would appreciate it if you could explicitly describe the difference between the results observed at 77K and 20K, as this statement does not convey the clear difference between 77K and 20K

pp. 2: We observe that by passing through its slip-twinning transition, this alloy reverses the sequence of mechanisms occurring at higher temperatures: twins activate first, generate a plastic zone, and produce dislocation sources.
I agree that twinning and ω-phase interactions can be a source of dislocation from a stress concentration source. However, the critical stress for dislocation formation from such stress concentration sources is generally much higher than the critical stress required for the motion of pre-existing dislocation. I think any discussion of critical stresses for dislocation motion needs to clearly show these differences.

pp.3: This is associated with a corresponding rise in the instantaneous strain-hardening rate immediately following yielding, as shown in Extended Fig. 2.
In Extended Figure 2, dσ/dε is shown; I am curious if there is also important information to be obtained for the Considere condition, (dσ/dε)/σ 1=0.

pp.3: To understand how these mechanical responses are related to fracture behavior in these two different stress-states, i.e., uniaxial tension and crack-tip triaxiality
pp.3: These observations underscore that stress-state plays a critical role in governing fracture mode.
The difference in fracture morphology due to different stress conditions is of great interest. The results from fracture surface observations were discussed here, but I would like to know if you could discuss the specific stress components that caused these differences.

pp.4: Figure 1, Note: the slightly lower crack-initiation toughness (K_{JIc}) at 77 K compared to 20 K is attributed to the statistical variation related to processing defects in the tested samples.
If possible, please include statistical variation by STD in the figure as in Fig. 1 C?
Please define what R is for J-R curves.

pp. 4 :show conventional {112}<111> nanotwins…
pp. 5: Although it is unclear whether the crystal collapsed to ω first to aid twin nucleation second, or if the local shear stress from the twins triggered the ω transformation Fig. 2(c)
Did the authors observe any {332} (113) Twin as observed in β-Ti alloys? Also, please provide any physical background why {112}<111> Twin is more likely to form in this alloy system.

pp.4: Our investigation began by analyzing the sequence of tensile deformation mechanisms. Tensile specimens, one interrupted shortly after yielding ($\varepsilon_p=0.004, \sigma_e\approx1000$ MPa), and a second at the uniform elongation (($\varepsilon_p=0.051, \sigma_e\approx1150$ MPa) were jet polished and examined in a transmission electron microscope (TEM).
Please clarify what you mean by ε_p and σ_e. If ε_p refers to plastic strain, please explain how you measured it.
In "At higher stresses, specifically at the ultimate tensile stress of 1150 MPa (which corresponds to 5.1% engineering plastic strain)," it is clearly stated that this is plastic strain. The expression should be consistent.

pp. 5: bowing into mixed orientations from pinning-depinning events
Please specify whether the pinning-depinning event is due to the presence of twins or to the solid solution states in HEA?

pp. 5: with a nanometer-thick interfacial layer of ω that expands in the space between the twins.
Based on the analogy from βTi, ω phase is expected to be formed in the region with high concentration of Gr. IV elements. Is it possible to analyze the elemental distribution in the region where ω phase and Twin are formed? Is the APT observation shown in Extended Fig. 14 and 15 actually related to ω phase formation?
Is it correct that unlike the TRIP effect, the ω phase itself does not play some role in plastic deformation?

pp. 5: At higher stresses, specifically at the ultimate tensile stress of 1150 MPa (which corresponds to 5.1% engineering plastic strain), bulk dislocation glide is activated.
Is the dislocation activation discussed here due to dislocation formation from a stress concentration source due to the twinning mentioned at the beginning, or is it due to kink formation of the pre-dislocation?

pp. 6: There also exists a marked stress gradient.
What is the effect of stress gradients on the activity of plastic deformation modes?

pp. 6: These kink bands are formed by $a_0/2$<111> dislocations with a {110} glide plane
I understand the idea of earning plastic deformation by kink bands. I guess that the formation of kink bands by blade dislocations is accompanied by crack slowing at the crack tip. I would appreciate a detailed discussion of whether this means that blade dislocations are easier to form specifically in this alloy system.

pp. 7: This is especially problematic since as a low symmetry hexagonal phase incapable of plastic deformation, ω can also act as a site for crack nucleation.
I would expect that the formation of the ω phase would prevent the stress concentration~fracture initiation, corresponding to

the ease of nucleation of dislocations from such a stress concentration source. Is this true? Is it possible to demonstrate this based on the fundamental material properties in this alloy system?

pp. 9: we show that the constraint imposed by stress-triaxiality prevents crack-tip stresses from reaching the screw glide threshold, limiting crack-tip plasticity to twins and edge dislocations that redistribute twinning-induced damage.
I believe that the effect of stress distribution at the crack tip on dislocation and twin formation is a universally important discussion that does not depend on the material. I would like the authors to describe what stress components in the triaxial stress state would bring about the above features.

pp. 12: J-integral was calculated as a sum of the elastic, Jel, and plastic, Jpl, strain energy contributions, Eq (6)
I believe that estimating the J-integral from experiments can lead to important information. I have not been able to fully follow the derivation of the plastic component of the J-integral in equation (6), but please mention how accurately it can be estimated.

Extended Figure 11.
The caption ends with an incomplete sentence.

Overall comments:
There is no doubt that plastic deformation mechanisms other than dislocations are important for ductility and toughness. On the other hand, dislocations have the important feature that plastic deformation can be continued by Flank-Read sources (especially in FCC) and other growth mechanisms. How much plastic deformation can be induced in twinning and phase transformation will be the key to improving mechanical properties by utilizing other deformation modes. Therefore, I would appreciate it if you could mention the plastic deformation capacity and its limit that the twinning and ω transformation in this study can produce.
Also, the authors discussed that twinning and phase transformation occur in the initial stage of plastic deformation. Is it possible to specify the relationship of critical stresses between twin formation and phase transformation?

The alloy Nb45Ta25Ti15Hf15 is composed of Group IV and Group V. Recent research has helped us understand the role of Group IV, but we are very interested in how the detailed composition of 45-25-15-15 was designed. Is it an optimal design for mechanical properties such as plastic deformation capacity and toughness? I know that it is difficult to investigate on an ad hoc basis due to the large degrees of freedom, but could you please add any data you have on the effect of the concentration of each constituent element on these properties?

Referee #3

(Remarks to the Author)

Congrats on the nice and detailed work.

Topic is interesting and results are in part also interesting, however, I have a few concerns on the interpretation and explanations, as outlined below, specifically on the mechanism explanations that are given to explain this response.

Divergent deformation pathways: not so clear what that means. See below, is a bit ill-defined. The interplay of slip and twinning is a very normal ting, also in BCC, or is s t else meant by that?

Question when I see this composition: Hf and Ti – containing alloys are extremely prone to severe H embrittlement, due to dislo core structure changers and hydride formation, particularly at defects such as dislos and GBs. This should be very critically discussed I suppose (likewise, replacing Hf by Zr or more Ti in these types of BCC 'Senkov' taste alloys leads to the same type of environmental H- related embrittlement). For example, did the authors look at the response of samples under non idealized / real world conditions with e g some water content or so (the corrosive surface water splitting leads to ingress of atomic H in Hf-containing alloys). These aspects deserve attention, in order to not heal the DBTT problem from C, N, S etc but making the material instead vulnerable to H. (this is NOT to be confused with 'normal' H-embrittlement in BCC alloys / steels etc as these are no (localized) hydride formers).

Not clear what is meant by 'multi-character dislocation glide' ? When rather clean, all these BCC alloys and BCC steels operate by 48 slip systems (see original papers by authors such as Christian and Sir Peter Hirsch e g). In FeSi it can be also twining plus 48 slip systems. In Ti – Nb / Ta / V etc it is dislo, twins and omega etc. - Is that meant here ?

Best check for a few statements: '…severely restricting their operating conditions..' is quite an overstatement, when considering the 1.7 billion tons of BCC steels produced and used every year… this is not quite underpinning such as statement as 'severely restricting their operating conditions' ; -) Also, this transition depends very strongly on C, N, S content etc as is well known, and modern BCC alloys do not really suffer from this limit, because they can be made very clean. Even artic region structures are since decades made of BCC steels, just that they are well controlled w r t cleanliness.. so better rewrite…

Omega phase forming BCC alloys are well studied in the Ti- Nb / Mo / V etc systems and local transformation to the omega phase is very well known – is that also observed in such simpler materials ? The fact that omega phase is formed does not require such as complex composition I guess. What is here the exact role than of this compelx composition ? Why this specific Nb / Ta ratio ? What is the rationale alloy design principle behind it ? How was this specific composition derived ?

I do not quite understand this sentence: 'By inverting the sequence of deformation mechanisms typical in metals and alloys, tensile yielding and crack-tip blunting are controlled by temperature-insensitive defects that generate dislocation sources at interfaces where they intersect and elevate the local stress.' Inverting what ? dislos are active from the first moment, already in the microplastic regime in BCC. Dislos in BCC are for sure NOT temperature-insensitive mechanisms, but due to their high Peierls barriers they are actually much more temp-sensitive than FCC dislos. Maybe check this sentence – I could not really understand what the message is here. Maybe I misunderstood it?

Statement 'This process keeps…' there was not really a process explained ? Also, any defect activity in BCC is very rate dependent – so that it would be interesting to see at which specific rates these statements holds and where they break down.

Statement is not quite correct: '…from the temperature sensitivity of screw dislocations which are significantly less mobile 50 than their edge counterparts, and rely on the thermally activated double-kink nucleation process..' What the authors mean likely is NOT formation of double kinks- this would mean two consecutive kinks of the same sign – BUT instead the likely refer to a kink PAIR, i.e. a plus and a minus kink (this the motion nucleus), which in themselves are actually + / - edge dislocations, and then the role of the secondary Peierls potential sets in. Better check such statements for correctness.

Statement: 'Consequently, facilitating screw dislocation glide through careful substitutional and interstitial alloying has been the governing principle to suppress the DBTT..' not quite sure if this is correct, because at low temperatures / higher rates the barrier for double cross slip plus FR source formation is the real barrier to replenish the dislo flux and comply with the imposed BCs, not so much the individual dislo motion, which actually depends a bot more on the segment length distribution. Sources always need the double cross slip, otherwise the BCC alloys undergo dislo starvation.

The loading rates are essential in this field. When I make tests ever and ever slower, I see such behavioral trends also – have the authors tested the results for several loading rates ?

Another boundary condition that is really essential in the field of measuring the ductile to brittle transition temperatures is the dwell time. This means that repeating the same tests after different waiting times gives completely different results, as particularly the literature on the refractory body centered cubic metals has often shown. Which is the reason? That many of these temperatures are plotted. Both as a function of time and function of purity, of course. So it might be interesting to repeat some of these tests after different dwell times And see if any trends are emerging similar as well known for. Binary refractory bodies in the cubic metals, for instance.

For many body centered cubic steels, it was shown that the grain size plays a very important role for the. Transition point. And hence the question whether the authors looked into this dependence here.

This statement about the underlying material design principle is not entirely clear w r t to the DBTT quest: 'alloying group IV into group V elements enhances screw dislocation glide by lowering unstable stacking fault energies. This combination of elements also reduces phonon frequencies, induces high lattice distortion, and when combined with high variations in local chemical environments intrinsic to RHEAs, increases the equilibrium concentration of double-kinks on screw dislocations that eliminates the need to thermally activate them'
The change in unstable stacking fault energies should be quite small, hence why is this a reason for the observed phenomenon ? Why should this promote kink PAIR formation ? (again: pls correct: these are not double kinks, they do not really help much here).
Why is it helpful is this alloy mix reduces phonon frequencies? I had assumed the opposite, i.e. that phonon are kionks little helpers, ie in BCC we want more of them, not less ? Is a bit counterintuitive or do I overlook s t here ?
Statement: 'induces high lattice distortion': given that we talk about BCC here these elements do not cause 'high' lattice distortion, all distorions stemming from interstitials in BCC are much much higher, but these do not help but instead counteract DBTT reduction. These is s t not quite lining up in this discussion. Also Cr or Ni etc in BCC Steels have also rather high distortions.
The statement 'high variations in local chemical environments intrinsic' is also not quite clear because BCC Fe Cr (Mn Si Al) C steels (and many others) have also very complex local chem landscapes, this reads maybe a little bit handwaving and less like a clear thermodynamic alloy design approach. Maybe these points need to be explained and justified and proven in more detail to leverage this response ? Reads otherwise scientifically not quite so clear.

The statement that all these effects reduce edge dislocation mobilities is not so clear. The edges are not really relevant as the authors stated above (excepts for those that move with near sound speed along the Peierls ridges). Not so sure about the chain of thought here.

Likewise, the claimed decrease in twinning stresses and promotion of the transformation to the omega phase at cryogenic temperature is well know for BCC-Ti/Zr/Hf alloys and is usually well known to very embrittling. This seems to contradict the literature on the role of omega ?

Yes, agree: the competition between dislocation glide stresses and twinning stresses that is important (but it is not completely temperature insensitive) but I had assumed that the dislo stress remains ALWYS below that for twinning in such alloys – this depends BTW very highly on grain size – was that checked here ? When GS goes down, dislo nucleation becomes hard and twins take over – this migth be essential here – however this is NOT due to the screw mobility in BCC, BUT due to the missing / insufficient bow out length required for FR / double cross slip and FR formation vs twinning out if the GBs. So this response should be highly GS dependent.

Statement: 'The bcc RHEA also contains interstitial elements: 0.059 at. % (60 ppm) C, 83 0.0495 at.% (58 ppm) N, and 0.146 at. % (197 ppm) O. Is the H content known ? This is game changer when Ti and Hf are used. Otherwise tis material of course super clean, where actually most BCCs have very low DBTTs.

Fig 1: better also always show the derivative of stress stran curves in addition, otherwise hard to see mechanism transitions.

Strain rate was very low, was any higher strain rate tested ? see explanations above.

Statement 'twinning overactivates and delaminates the grain boundary' not so clear. The GB is the start point / and sometimes also the end point of a twin, why is a twin delaminating a GB ? not so clear.

I understand from the paper that all the dislocation characterization that is presented in Figs 2 and 4 is post deformation. This means that all the screw dislocations are relaxed back to their energy valleys. So no direct proof is given for the claims of the change in the activation mechanism change that was discussed before. In other words, the statistics shown in the paper is quite nice, however, it is not really underpinning what is claimed as the underlying reason for this change in the DBTT. Seems to be quite pertinent to make a closer connection between these dislocation observations and the actual mechanism claims (see above, SFaults, phonons, kink populations under load etc.). This link is missing. Dislocation analysis in such BCC materials typically is done under load and not after relaxation when aiming to show changes in the kink and double crosslip behaviour.

One essential point that is still puzzling me after reading the paper is why the composition has to be so very complex and why the same main trade off question, namely between twinning and dislocation kink activity cannot also be achieved by much simpler alloys. This point has not really been explained very well. This point relates a little bit to the other comments above, where the rationality behind this specific alloy composition has not been very well explained. What is the alloy design sweet spot that requires this specific alloy composition? What is the main physics handle here? That was left a little bit unanswered. Why does it not work on Fe Si or Ti Nb etc (where dislos, twins and omaga etc are long known to interact.). Maybe it would be helpful to plot corresponding reference data on such a simpler alloy also.

Version 1:

Reviewer comments:

Referee #1

(Remarks to the Author)
A. Summary of the key results
(includes statements of my previous assessment) Body centered cubic (BCC) metals and alloys exhibit characteristic transitions from low to high ductility/toughness when surpassing a certain temperature – the brittle-to-ductile Transition temperature (BDTT). This type of embrittlement obtained at low temperatures is often limiting the engineering application scenarios of otherwise interesting candidate materials.
In the present manuscript, the authors provide a comprehensive analysis of the low temperature mechanical performance, incl. ductility and toughness, of concentrated solid solutions, i.e. $Nb_{45}Ta_{25}Ti_{15}Hf_{15}$ as well as $Nb_{70}Ti_{30}$. The data impressively shows that the alloys, in the microstructural condition investigated, exhibit reasonable ductility (not only strain

to fracture but also uniform strain) as well as toughness even at 20 K. For most application scenarios, no relevant BDTT is thus obtained. It is specifically noteworthy that this is the case for both assessment types, i.e. ductility and toughness, which is not often provided at the same time in literature. Furthermore, the authors appended the investigation on the binary Nb-Ti alloy during the revision process proving that the findings are general and not related to the chemical complexity of the quaternary alloy. The microstructural and theoretical assessment of the finding reveals that an unusual combination of deformation mechanisms contributes to this behavior. In the present case, deformation twinning and phase transformation mediated plasticity prevent a temperature-dependent increase of strength at low temperatures. This behavior mitigates a strong constriction of the plastic zone at the crack tip.

B. Originality and significance: if not novel, please include reference
The findings are surprising and interesting to the field of physical metallurgy, the quality of the provided data is high, the assessment sound and statistically relevant. Thus, I recommend the manuscript for publication. The authors have addressed relevant aspects raised by the reviewers. Reviewers no. 2 and 3 have raised important points, for example regarding hydrogen embrittlement and strain rate impact. I agree that these are fundamental issues to be investigated. The authors convincingly outline that the present study is limited to fully exploiting these topics in the relevant depth at the moment. The limitation to quasistatic conditions is reasonable and an upper rough estimation for the H content was provided by APT to put the investigations into perspective.

C. Data & methodology: validity of approach, quality of data, quality of presentation
Minor stylistic aspects might be improved regarding the presentation:
- Please check the capitalization of the vertical axis descriptions in Figs. 1 and ext. 2.
- Please unify the true strain notation in the main text and the mechanical characterization section, ll. 358-361.
- I recommend introducing “electron _backscatter_ diffraction”, l. 456.
- There is a superfluous “that” in l. 512. Section “Micromechanical models of stress-controlled fracture for an inverted mechanistic sequence”, ll. 507-534, should be cross-checked once more regarding the text.
- There is a superfluous “deformation” in the caption of ext. Fig. 10.

D. Appropriate use of statistics and treatment of uncertainties
The data is statistically robust. One remark by reviewer no. 2 was addressed by referring to literature results as the material under investigation is currently limited in quantity. This is reasonable.

E. Conclusions: robustness, validity, reliability
The assessment and conclusions are reasonable and display an important finding for the community. As described above, there are open questions to be resolved (rate dependence, grain size dependence, hydrogen/interstitial impact) which seems reasonable given the importance of the discovery. I consider this work to be beyond the scope of the present article and I’m convinced that the community will take up the principle presented here.

F. Suggested improvements: experiments, data for possible revision
None.

G. References: appropriate credit to previous work?
This aspect has appropriately improved over the previous version.

H. Clarity and context: lucidity of abstract/summary, appropriateness of abstract, introduction and conclusions
This aspect has appropriately improved over the previous version.

Referee #2

(Remarks to the Author)
I appreciate the authors' sincere attitude in addressing the reviewer's comments, including additional TEM observations, and elemental characterization using APT and TEM-EDX. I confirm that the authors have provided clear and convincing responses to the questions raised by reviewers, based on their deep knowledge and insight into materials science and mechanics. The design strategy for the alloy system in this study is also very clearly presented and may serve as a useful guideline for the future design of HEA and solid solution alloys. The proposed deformation mechanism represents an excellent finding that could help overcome the intrinsic issue of low-temperature brittleness in conventional BCC alloys, and I look forward to its publication. I also expect to see that the formation of dislocations within twins and the subsequent complex processes will be further elucidated through MD simulations using MLP.

* I have one final question. The authors' additional analyses have clarified that the segregation of Group IV elements at grain boundaries serves as the chemical driving force for the omega-phase transformation. While the authors have also explained the relationship between the omega phase and twins from an atomic-scale perspective, it remains unclear how the formed ω phase influences deformation governed by dislocation motion and twinning. In particular, how does the presence of the ω phase affect macroscopic plastic deformation behavior?

Although the coexistence of the ω phase and twins is frequently observed, there appears to be limited discussion on their combined impact on macroscopic plasticity. In the present alloy system, the omega phase forms due to compositional fluctuations, and its elastic constants might differ significantly from those of the matrix phase. While the authors have addressed its potential contribution to embrittlement, it would be very interesting to further understand the mechanisms by which such microstructures lead to fracture.

This point may also be addressed as part of the future outlook of this study.

Referee #3

(Remarks to the Author)

See last comments about vague title…: not much better now ... The new title: 'Inverted deformation sequences inhibit ductile-brittle 2 transitions…' is likewise very unclear: 'Inverted deformation sequences': Not so sure if this makes things better, title is still pretty unclear. What exactly is inverted here? As stated in the last review cycle: sequence & interplay of mechanical twinning and dislocation activity is a very normal thing in many BCC alloys (see overview papers ch as that by Christian and Mahajan in 1995 (Progr in Materials Science) and the papers of Sleeswyk and Seeger etc. in which many alloys such as Fe, Fe Cr, Ti – Mo / V / Cr / Fe / Nb etc alloys and all the Fe Si alloys etc as well as many Mo alloys etc were reviewed w r t twinning and screw core effects. There is however no such thing as a strict 'inversion' (?) or very strict sequence (see e g original single crystal papers from the 70s and 80s on BCC with twins). There is an immense temperature and rate dependence, orientation dependence, Schmid factor dependence etc - not sure if this 'fancy' terminology really aids or blurs the view here.

'ductile-brittle 2 transitions…' plural is by purpose – are the authors sure ? Figure 1 does not cover the use of a plural here ? Or did I miss s t here ? I see only one transition.

'catastrophic drop in toughness': depends in BB very highly on alloy cleanliness. For very clean alloys it is not 'catastrophic'.

Are the authors really sure to claim that 'plastic deformation begins with twinning' ? This is practically impossible I guess…. You need at least a few partials to nucleate a BCC twin I suppose. Hence, no dislocations and spontaneous twins… ? not sure of this can be true. The activation stress of a wide dangling segment and it s double cross slip plus FR source is happening at very low stresses. I would tend to revisit such a claim and be rather very careful.

Unclear: twinning in BCC is not a 'temperature insensitive defect': very well documented e g for Fe Si and Fe C etc that twinning is temperature- and rate dependent. In general, I suggest that a bit more care is taken in considering the original literature on BCC twins and reflect more crisply, what exactly the novelty is compared to the know physics dating back to the 1950s.

The tendency of BCC metals and alloys to deform by mechanical twinning rather than dislocation slip ALONE is a deeply temperature-sensitive phenomenon; mechanical twinning in BCC is thermally usually more suppressed at elevated temperatures, where mobile screw dislocations more easily accommodate plastic strain by slip, and progressively activated at lower temperatures, where screw dislocation mobility is frozen out and the locally elevated stresses drive twin nucleation. This understanding, rooted – as explained again in this paper - in due to the core structure of BCC screws, and remains the foundation for BCC design and DTB transition rationales till today. See e g the recent sunmary in https://pubs.aip.org/aip/jap/article/134/5/055102/2905122/Competition-between-full-slip-and-twinning-in-BCC and others.

I am still not satisfied wit the slightly handwaving discussion about the claimed inversion: early and quite direct demonstrations of the screw / twin and (later) twin / omega temperature dependence was e g given by Hull in 1961 (about the former) in tensile deformation experiments on Fe–3Si across a wide range of temperatures (from room temperature down to –253 °C !). He very clearly showed that as temperature dropped, twinning became increasingly prevalent at the expense of slip, providing one of the first quantitative datasets linking temperature to the slip-to-twin transition in a BCC system. Thornton and Mitchell also showed the same trend in 1962 for a broader class of alloys under cryogenic conditions, demonstrating that the competition between slip and twinning is a general feature of BCC low-temperature plasticity rather than a peculiarity; see e g https://www.sciencedirect.com/science/article/abs/pii/0001616061900694
Along similar lines, Bolling and Richman also looked at temperature-dependent twinning behavior in BCC in their paper series on continual mechanical twinning in Acta Metallurgica in 1965 and found there BCC alloys that deform almost exclusively by twinning over a wide temperature range, and they were able to isolate the flow behavior associated with twinning and characterize it independently of slip contributions. What is quite remarkable is that they found that in regimes dominated by twinning, the flow stress displayed a positive sensitivity to temperature and a negative sensitivity to strain rate — the exact inverse of what is observed for BCC slip, which is thermally activated. This anomalous behavior attracted considerable attention and helped motivate a more mechanistic understanding of why twinning in BCC metals behaves so differently from its FCC counterpart. Best look at their papers e g here https://www.sciencedirect.com/science/article/abs/pii/0001616065901367 to place ur work in to a better perspective of what is already state of the art in this field. (the mechanistic foundations are still subject to debates of course to some extend)

Also, on the 'sequence' discussion (see critique above) I would also recommend to look a the excellent papers of Sleeswyk

in 1962 (see your citation 51) and later in 1974, focusing on ½⟨111⟩ screw dislocations in the BCC lattice and connecting their tendency to adopt a non-planar, sessile core configuration at low temperatures to the nucleation barrier for twinning. The physical picture that emerged from these studies is that the same core structure responsible for the high Peierls stress of BCC screws — and thus for the steep temperature dependence of BCC yield strength — also controls the stress concentrations necessary to nucleate a twin. Some of these points overlap with some of the content of this paper, yet, it might help a bit to avoid any 'overclaiming' or sequential dislocation-twin pathways that are not likely to be true; as these two defects are very closely linked in their activation. I can only firmly underlin that these papers from the 1950s onwards looked in part in a very details manner at these links.
Also, Priestner and Leslie in 1965 modeled twin nucleation at slip-plane intersections and argued that the dramatic increase in yield stress at low temperatures generates the local stress concentrations required to operate a twinning mechanism. This is in my view a very likely connection to be considered in the argumentation here.
In other words, it is precisely because BCC screw dislocations become so immobile at low temperatures that the applied stress can reach the critical twinning stress before the material yields homogeneously by slip. The slip–twin transition is therefore not an arbitrary threshold but a natural consequence of the differing temperature sensitivities of the two competing deformation mechanisms.

'predicted to be intrinsically ductile at 0 K…' … Nernst Theorem ?

'…it is also unclear how to reliably predict precise twinning stresses, but general trends indicate that alloying which promotes slip nearly always lowers twinning stresses relative to pure elements..' The paper is sometimes imprecise and a bit handwaving on some of these interaction mechanisms: see e g the very broad overview of these observations which was compiled already in the 1973 paper by Mahajan and Williams (and several others…. ; see above), who synthesized most available experimental data across a range of BCC metals and alloys. They concluded that BCC metals exhibit a negative temperature dependence of the critical twinning stress — meaning that twinning becomes easier as temperature falls — though the precise relationship is material-dependent and modulated by alloy composition, grain size, and strain rate. This paper was not considered here. Similarly, a comprehensive treatment of the subject is quite eminent review by Christian and Mahajan in 1995, Progress in Materials Science, a paper not considered by the authors... The BCC related part of this classic review synthesizes decades of experimental data and mechanistic modeling into a pretty coherent picture: below a critical temperature that shifts upward with increasing strain rate, the critical resolved shear stress for twinning becomes accessible before or alongside slip, and twinning emerges as a primary or co-operative deformation mode. This transition is not merely a low-temperature curiosity but has direct consequences for the ductile-to-brittle transition in BCC steels and alloys, since twins can act both as carriers of plasticity and as potent crack nucleation sites. This duality is also not really discussed in the current paper, although it had been addressed in vcery deep details before by Christian and others. In other words, some of the claims or argimention bits were already published (even with a more precise wording in part) more than 50 years ago – better make sure to consider these works on properly cite them when similar aspects are discussed.

"The expansive set of current and potential applications for bcc alloys, from building and transportation infrastructure to energy technologies, are often limited by this transition." I understand that the authors want to underpin the importance, but again, extreme cryogenic applications are not so limiting as claimed here and in these fields usually FCC steels fo a good job, so better avoid any overstatements.

I come back here to my last comments in the last review cycle : 'I do not quite understand this sentence: 'By inverting the sequence of deformation mechanisms typical in metals and alloys, tensile yielding and crack-tip blunting are controlled by temperature-insensitive defects that generate dislocation sources at interfaces where they intersect and elevate the local stress.' Inverting what ? dislos are active from the first moment, already in the microplastic regime in BCC. Dislos in BCC are for sure NOT temperature-insensitive mechanisms, but due to their high Peierls barriers they are actually much more temp-sensitive than FCC dislos. Maybe check this sentence – I could not really understand what the message is here. Maybe I misunderstood it?'
And I see that the authors stick to there wording of the 'inversion' and also use it now as a title – in view of the comments made above I still do not understand what they mean what exactly is inverted (compared to exactly what?). The many older papers cited above (and there are many more, properly done on high purity single crystals…) show clearly that these deformation mechanisms do not follow a linear sequence but they are highly nonlinearly linked. Some of that is simply not acknowledged by the authors here . I do not see the inversion argument here.

Version 2:

Reviewer comments:

Referee #3

(Remarks to the Author)
In my humble view, the manuscript continues a bit to rely on a number of rather impressionistic arguments and appears to

conflate aspects of the well-established physics of BCC twinning and ω-phase switching—phenomena that have been extensively documented in systems such as Nb / Ta / Mo blended with Zr / Hf / and Ti. I unfortunately find myself with little to add beyond the points already articulated in my previous reviews. At present, I remain a bit unconvinced by some of the the central claims of the paper and their claimed novelty character and by the evidentiary basis on which they rest. Hence, I regret I cannot fully support this paper.

Open Access This Peer Review File is licensed under a Creative Commons Attribution 4.0 International License, which permits use, sharing, adaptation, distribution and reproduction in any medium or format, as long as you give appropriate credit to the original author(s) and the source, provide a link to the Creative Commons license, and indicate if changes were made.
In cases where reviewers are anonymous, credit should be given to 'Anonymous Referee' and the source.
The images or other third party material in this Peer Review File are included in the article’s Creative Commons license, unless indicated otherwise in a credit line to the material. If material is not included in the article’s Creative Commons license and your intended use is not permitted by statutory regulation or exceeds the permitted use, you will need to obtain permission directly from the copyright holder.
To view a copy of this license, visit https://creativecommons.org/licenses/by/4.0/

RESPONSE to REVIEWERS' COMMENTS

At the outset, we would like to thank all the reviewers for carefully reading the paper and providing commentary on how to improve it. In response to a few questions posed by the reviewers, primarily on the role of chemical complexity on deformation mechanisms and the DBTT, we decided to perform the same set of experiments on a chemically simpler, but electronically similar $Nb_{70}Ti_{30}$ alloy. The extensive set of experiments has delayed our response and for that we apologize. We have made some significant changes to the text of the paper; however, the main story and results remain the same. These changes are made to accommodate the new results in the main text, generalize the paper to discuss *bcc* refractory alloys in general, insert some discussion on strain hardening, and simultaneously remove some text for the editorial team. We think that these new experiments and resulting analysis strengthen the paper. We hope you all concur.

Referee #1 (Remarks to the Author):

A Summary of the key results

Body centered cubic (BCC) metals and alloys exhibit characteristic transitions from low to high ductility/toughness when surpassing a certain temperature – the brittle-to-ductile transition temperature (BDTT). This type of embrittlement obtained at low temperatures is often limiting the engineering application scenarios of otherwise interesting candidate materials.
In the present manuscript, the authors provide a comprehensive analysis of the low temperature mechanical performance, incl. ductility and toughness, of a concentrated solid solution, i.e. Nb45Ta25Ti15Hf15 formerly introduced in https://doi.org/10.1126/science.adn2428. The data impressively shows that the alloy in the microstructural condition investigated exhibits reasonable ductility (not only strain to fracture but also uniform strain!) as well as toughness even at 20 K. For most application scenarios, no relevant BDTT is thus obtained. It is specifically noteworthy that this is the case for both assessment types, i.e. ductility and toughness, which is not often provided at the same time in literature. The microstructural and theoretical assessment of the finding reveals that an unusual combination of deformation mechanisms contributes to this behavior. In the present case, deformation twinning and phase transformation mediated plasticity prevent a temperature-dependent increase of strength at low temperatures. This behavior mitigates a strong constriction of the plastic zone at the crack tip.

B Originality and significance, C Data & methodology, D Appropriate use of statistics and treatment of uncertainties, E Conclusions

The findings are surprising and interesting to the field of physical metallurgy, the quality of the

provided data is high, the assessment sound and statistically relevant. Thus, I recommend the manuscript for publication after addressing my comments provided in the following.

F Suggested improvements

We would like to thank the reviewer at the outset for carefully reading and commenting on our paper. We have addressed the questions below and performed additional experiments to further contextualize our results. Due to the addition of these experiments to the main text and supplementary figures, as well as the request from editors to reduce the length of the main text, we have made substantial changes to the manuscript; however, the findings remain the same. We hope that our additional work and changes are satisfactory.

In contrast to other publications, the authors provide data on the contamination levels. The authors might append the information that the ppm are indeed weight-ppm. The reported contamination levels are comparably low which is important for the assessment of phase stability and transformation induced plasticity. Thus, I recommend including an assessment of the data for the reader with reference to literature.
The material under investigation is stated to be “near-fully” recrystallized. Indeed, the electron backscatter diffraction (EBSD) data in the Extended Data reveals substantial deformation being left in the microstructure. (i) With respect to the presentation of the EBSD data, the pattern quality maps should always be provided separated to allow for the assessment of localized features (twinning/transformation/kink banding/etc.). (ii) For the assessment of the recrystallization progress, EBSD data on the rolled condition should be provided for proper assessment; 1000 °C/1 h are indeed low/short while the grain size is comparably coarse for static recrystallization of a concentrated solid solution. Thus, proof of any kind of recrystallization (as opposed to recovery) is required. (iii) The impact of remaining dislocations on the obtained behavior remains elusive – however, proper pre-deformation was previously proven to substantially affect ductility and BDTT of refractory elements.

We thank the reviewer for the insightful comments on the role of microstructure on the DBTT. We have added an additional supplementary note that discusses the role of microstructure in reference to the literature and acknowledge some limitations that this presents to our study. We have also clarified that the contamination levels are indeed weight ppm.

i) We have added pattern quality maps to the supplementary information. There is some residual deformation in the microstructure.
ii) We have performed studies on the recrystallization and creep behavior of this alloy in a separate paper. We have found that recrystallization in this alloy occurs at temperatures beginning above 900°C (at least on the time scale of 3 hours for this temperature, which is a very short time period).[1] At 1200°C, we observe dynamic recrystallization, which occurs within several minutes of exposure to this

temperature.[2] Studies on a similar alloy (with 5% Nb swapped for 5% Zr) from one of our collaborative groups show the same behavior.[3] The ease in which recrystallization occurs is due to the lack of particles pinning grain boundaries and producing Zener drag forces. While this is not a good property from the perspective of high temperature applications, it eases the processability of the alloy.

iii) We agree that partial recrystallization has a large effect on the DBTT, and we have included additional citations and discussion in the manuscript. One caveat here is that partial recrystallization removes the need for dislocation sources and makes it such that the determining parameter governing the DBTT is kink pair activation, and not dislocation source starvation.[4,5] This can also help ductility at low temperatures in Nb alloys.[6] When we originally performed these experiments, we believed that this was a contributor to the low DBTT. However, our TEM observation indicate that the pre-existing dislocations cannot move at yielding. We also observe that dislocations only begin to move after twins produce them. We have performed additional TEM and appended fig. 4 to show this process. Thus, the alloy has a mechanism by which it can produce dislocations that glide, and to yield and plastically deform at stresses below those required for dislocation glide. We have acknowledged in the manuscript that the dislocations we observe at 4.9% plastic strain could come from a combination of the nucleation from twins or pre-existing dislocations in the microstructure However, partial recrystallization can raise strength through forest hardening, thereby inhibiting dislocation glide, particularly at low temperatures.[3,7] This can have deleterious effects on cryogenic toughness.

The atmosphere in the stainless-steel container during the heat treatment should be mentioned.
We have added this information to the methods and materials section. The samples were sealed in stainless steel bags that were welded shut, and heat treated in air.

I recommend including the strain hardening curves into Fig. 1a to visualize the ability of the material to uniformly deform. This is unusual for BCC metals and alloys at such low temperatures and an important finding.
We have included these curves in the figure. To prevent making figures that are overly crowded, we have moved the fracture surface images to their own figure which has made observation of microvoids (mentioned in the following comment) much easier.

The microvoids indicated in Fig. 1e are not to be identified by the reader. The micrographs should be revised to properly prove the statement.
We have moved the micrographs to a separate figure which makes them more easily identifiable, as mentioned in the previous response.

The authors provide evidence that the sequence of deformation mechanisms is changing as a

function of temperature, specifically from 77 to 20 K. However, the strain hardening does not change. This needs to be discussed. With respect to the common (and potentially not correct) interpretation of twinning/transformation induced plasticity as a dynamic microstructure refinement process, dislocation activity (to promote hardening by the additional boundaries) might be required to explain the strain hardening. This would not be the case if exclusively deformation twinning is active. A mechanistic discussion is required to explain why strain hardening remains the same for 77 and 20 K.

There is a subtle change in the strain hardening, and we agree that the microstructural refinement argument is not quite correct for this case. The instantaneous rate is nominally the same at both 77 K and 20 K in the first ~4% plastic deformation but the hardening is not sustained at 20 K, whereas it plateaus at 77 K. The reason this occurs is now mentioned in the discussion section. Essentially, twinning and slip are competitive processes in *bcc* alloys. In the same grain, a {112}<111> twin can nucleate from a screw dislocation instead of that screw dislocation gliding.[8] The twins therefore transform the microstructure into plates with varying Schmidt factors. As thew stress increases, the dislocations that have the fewest barriers to glide activate first and channel along highways of highest Schmidt factor (we have included an extended figure showing this). At 77 K, it is easier for dislocations to move and so they can more easily activate homogeneously which promotes uniform ductility. At lower temperatures, it becomes more difficult and so dislocation glide is confined to regions where it is easiest, promoting strain localization.

The authors state "It is not initially intuitive as to how twinning can cause intergranular fracture. After all, it is a form of plasticity." This needs further elaboration. In contrast to dislocation slip, deformation twinning will always result in a very confined shear strain accumulation within the surrounding material (depending on the thickness of the twin) at the twin "tip"/termination. This might particularly be relevant when twins approach grain boundaries. The localization aspect anyway comes a bit short in the discussion.

Based on the investigations, it is not possible to conclude whether twinning or transformation comes first. I agree with this. Hence, the terminology "omega wetting twin boundaries" is not suitable as it suggests the sequence of twins forming first and then omega on their boundaries.

We agree that this statement in the introduction is oversimplified and therefore incorrect. We have removed it and rewritten this section. What we originally intended to communicate is that when twinning occurs during TWIP processes, it often enhances ductility by preventing necking. However, this is not always the case (as is seen at low temperatures in some refractory *bcc* materials).

We did not originally intend for the term "wetting" to suggest an order to the mechanisms; however, we can see how the extension of this term to grain boundary phases can be interpreted to imply a temporal sequence. We have altered this terminology to talk about it as a transitional structure.

Deformation twinning can exhibit rather pronounced size dependences in BCC metals and alloys. This might need to be considered when the interaction of twins with grain boundaries and cracks are concerned. This is currently not properly addressed in the manuscript (please see the suggestions on literature on refractory elements and their alloys).
We agree with the reviewer that there can be a size dependence. We have included additional discussion in a supplementary file on the role of microstructure. However, literature suggests that grain size does not have an effect on this transition or mechanical behavior, at least in compression.[9]

The authors state "The inverted sequence, however, is critical to sidestepping deformation through dislocation glide, which would otherwise cause embrittlement." This is somewhat misleading. The dislocation mediated plasticity is strictly not the cause of embrittlement. With respect to ductility, it is the increasing yield strength and its competition to fracture stress that limits low temperature plastic deformation, not dislocation activity itself. With respect to cracks, a constriction of the plastic zone is certainly expected but this should be a continuous behavior and not causing a "transition". The statement requires more elaboration.
We agree with the reviewer that this is poorly phrased. We have rephrased this sentence.

It is noted that the appearance of twinning or transformation comes along with stress drops. This is not further mentioned and outlined but needs to be discussed in further detail. What are the stress drops seen at any temperature in the stress-strain curves presented?
The stress drops are twinning drops. We have added a statement to the main text. We detected acoustic emission at 77 and 20 K and attribute these stress drops to twinning.

Localized deformation, i.e. by twinning/transformation, will cause local heating which is of particular importance during low temperature deformation as heat capacity and thermal conductivity are very low. Is there any temperature surveillance during the tests to proof the local temperature, specifically when crack propagation is addressed?
I recommend appending the relevant mechanical data (yield strength, stress of first stress drop, ultimate tensile strength, uniform strain, strain to fracture, K_JIc, K_ss, etc.) to Table 1 to also provide quantitative insights to the performance of the alloy.

We have added the additional mechanical properties to the table.

The point about measuring the temperature rise during testing is interesting, and it is something that we measured, but cannot confidently contribute to plastic deformation. There are several confounding factors that make these measurements unreliable. First, the radiation-based temperature measurement systems such as pyrometer or IR camera doesn't work for these temperatures. Also, it's difficult to place thermocouple or a contact sensor on or near the location where plastic deformation is occurring as it would severely interfere with the stress-state and

invalidate our calculations. It is not allowed in the ASTM-E1820 standard. The second variable is that there is a small amount of friction in the grips that produces some heat. Because we first measure the temperature rise in the grips, followed later in the test sample, we believe that these temperature changes (which do not exceed 7 K) are most likely from the friction. This temperature change has already been factored into our averages.

We were also unable to measure the temperature during crack propagation for this same reason. We did not want the thermocouple to alter the calculated and geometry-sensitive stress-strain states in compact tension samples. Crack extension was generally stable during tests; we only measured unstable crack propagation once the crack was too long for validity.

G References

Deformation twinning in BCC metals and alloys is still not properly understood, specifically when compared to face centered cubic metals and alloys. Though, there are plenty of investigations on refractory elements and concentrated alloys thereof found in literature, pure Nb, Ta, Cr-Fe, Nb-V, Mo-Re, MoWRe, etc. The proper assessment of these reports comes a bit short in the manuscript. With respect to ductility and the activation of deformation twinning in group 4/5 concentrated solid solutions, the authors might consider a recently published pre-print from another group, https://doi.org/10.21203/rs.3.rs-6422281/v1.

We have added additional references including this one to our paper. Since we received this review, this paper has been published and so we have cites the published manuscript, instead. We agree that it is a very poorly understood mechanism and we hope that our insights into the fact it can control yield strength and invert typical TWIP mechanisms sheds further light on its nature in *bcc* alloys.

With respect to the other alloys, we now reference some of these as well. Alloying in bcc systems, generally always decreases twinning stresses, particularly if it shifts the VEC away from 6.[10] MoWRe is a particularly interesting case because it can activate this inverse TWIP mechanism at higher temperatures. These tests are performed in compression, and our conversations with the authors about this alloy have indicated that it is brittle in tension due to grain boundary embrittlement.

H Clarity and context

The title and abstract denote the present alloy as _medium_ entropy alloy. These are the only occasions when the term is introduced. In other instances, only refractory _high_ entropy alloys are named. In the current assessment, no evidence for the relevance of entropy is provided. While I understand that the naming might be relevant to attract attention, the basic mechanisms

seem to be specific to solid solutions or concentrated solid solutions (of group 4/5 elements). The designation might therefore be adopted.

We agree with the reviewer and have changed the terminology to talk about multi-principal element alloys and concentrated alloys. We agree that the differentiation between "medium" and "high" entropy is arbitrary and not scientifically rigorous. We have just adopted the term which has a historic meaning.

The authors frequently note twinning and transformation to be "overactivated". The terminology needs to be properly introduced and defined. While I can anticipate the background of the terminology, the use of such a concept has some restrictions with respect to a deformation mechanism which can never be "overactivated" from a plasticity point of view.

We agree that there is not hard cut-off between "over" or "under" activated. We have used the term simply to denote activation that is high enough to cause brittle failure. We have removed this term and stated this explicitly in the manuscript:

"However, these elements can act as "a double-edged sword" by promoting the transformation to omega (particularly at low temperatures), which is brittle in large amounts.[11,12] This is especially problematic for samples containing cracks because the high hydrostatic stress can promote stress-controlled transformations and exploit the brittleness of the new phase to accelerate fracture.[13,14]"

Referee #2 (Remarks to the Author):

This study discusses the mechanism by which Nb45Ta25Ti15Hf15 alloys produce a unique deformation mode called slip-twinning transition, which mitigates the sudden increase in yield strength due to temperature decrease and avoids DBTT caused by plastic deformation due to dislocations. We believe that this is an excellent discovery for an alloy that overcomes an intrinsic weakness. I believe it is basically worthy of publication, though I'm concerned that there is overlap with the discussion in Reference 20. I concur that this work is impactful and suitable for a prestigious publication like Nature. Nonetheless, I suggest some revisions to further refine the manuscript before recommending its acceptance.

We thank the reviewer for taking time to carefully read our manuscript and provide insight into ways in which it can be improved. We have answered the questions below, made relevant edits to the paper, and added additional experiments on another alloy to further contextualize our results.

pp.2: To evaluate how these mechanisms interact to affect this alloy's DBTT, we investigated its

mechanical behavior in tension at 20 K,…
pp.2: We observe that by passing through its slip-twinning transition, this alloy reverses the sequence of mechanisms occurring at higher temperatures: twins activate first, generate a plastic zone, and produce dislocation sources.
I would appreciate it if you could explicitly describe the difference between the results observed at 77K and 20K, as this statement does not convey the clear difference between 77K and 20K

We understand how this statement can be vague. We have added additional analysis of the strain hardening behavior into the results and discussion section and moved the strain hardening figure to figure 1. We have also removed those sentences as they are not very helpful in that part of the paper. Essentially, one of the main differences is that the strain hardening is not sustained over large strains at progressively lower temperatures. We attribute this to a competition between dislocation slip and twinning that progressively favors twinning at lower temperatures (this is elaborated in the discussion section) and we have added an additional figure to the supplementary information.

pp. 2: We observe that by passing through its slip-twinning transition, this alloy reverses the sequence of mechanisms occurring at higher temperatures: twins activate first, generate a plastic zone, and produce dislocation sources.
I agree that twinning and ω-phase interactions can be a source of dislocation from a stress concentration source. However, the critical stress for dislocation formation from such stress concentration sources is generally much higher than the critical stress required for the motion of pre-existing dislocation. I think any discussion of critical stresses for dislocation motion needs to clearly show these differences.

The reviewer raises a good point. At low temperatures, the mobility of screw dislocations in bcc metals is known to be controlled by kink-pair nucleation rather than kink-pair migration. The question then becomes, what are the properties of this alloy that enhance dislocation nucleation at low temperatures compared to elemental bcc metals?

We have observed that Ti and Hf substantially lower the USF energy, and according to the Peierls-Nabarro model for dislocation nucleation, that would be linked with a decrease in the critical stress for dislocation nucleation, favoring nucleation against migration.[15] Further, due to the soft vibrational spectra and large local lattice distortions in this RHEA, the equilibrium dislocation line profile is expected to be naturally kinked, which may also favor nucleation.[15]

Although not perfect, we can demonstrate this principle more quantitatively by comparing the stress required to nucleate a dislocation obtained from the gradient of the USF energy profile to the stress required to rigidly glide a dislocation obtained from the gradient of the Peierls potential.[2,16] We have obtained stress values equal to approximately 7.5 GPa and 6.5 GPa for

dislocation nucleation and glide, respectively. It should be noted that these stress values are considered a theoretical upper bound. Nevertheless, comparing the results for NbTaTiHf to pure Nb, the stress required for dislocation nucleation and glide in Nb is equal to 7.51 GPa and 0.79 GPa, respectively, presenting a much more substantial disparity compared to that found for NbTaTiHf.[17,18]

pp.3: This is associated with a corresponding rise in the instantaneous strain-hardening rate immediately following yielding, as shown in Extended Fig. 2.
In Extended Figure 2, dσ/dε is shown; I am curious if there is also important information to be obtained for the Considere condition, (dσ/dε)/σ 1=0.

We were only able to ascertain the true strain at necking using Considère's criterion.
We did not note any unusual behavior with this criterion.

pp.3: To understand how these mechanical responses are related to fracture behavior in these two different stress-states, i.e., uniaxial tension and crack-tip triaxiality
pp.3: These observations underscore that stress-state plays a critical role in governing fracture mode.
The difference in fracture morphology due to different stress conditions is of great interest. The results from fracture surface observations were discussed here, but I would like to know if you could discuss the specific stress components that caused these differences.

The reviewer brings up a great point. We have elaborated upon it in the main text and changed the figures such that figure 2 addresses difference in stress state. In a tensile sample the stress tensor only has one non-zero component, whereas the strain tensor has three non-zero components. In contrast, the stress-state at a crack tip is triaxial (the stress tensor has 3-5) non-zero components, whereas the strain tensor has two non-zero components. Moreover, these stresses are ~3 times higher than in a tensile sample and the strain is ~10 times lower. As a result, the hydrostatic stress is high, but the shear stresses are comparably low. The high hydrostatic stress exploits weak points in the microstructure that would fail in a brittle manner in tension and restricts the possibility for plastic deformation. We include this discussion at the end of the discussion section.

This is one of the primary reasons twinning is so effective at preventing the DBTT. It allows for plastic deformation to activate at shear stresses below what would be allowed if dislocation glide were controlling crack-tip blunting/tensile yielding. As a result, plastic deformation is activated at a stress below the critical one that would cause a brittle, local microstructure dependent failure.

pp.4: Figure 1, Note: the slightly lower crack-initiation toughness (K_JIc) at 77 K compared to

20 K is attributed to the statistical variation related to processing defects in the tested samples. If possible, please include statistical variation by STD in the figure as in Fig. 1 C?
Please define what R is for J-R curves.

There is statistical variation in defects that cause fracture to occur. This can be observed in both brittle and ductile materials. We do not have sufficient material to perform additional tests that would bring statistical significance to the test-to-test variation in the global fracture toughness, but it is entirely due to small variations in the local crack-tip microstructure. We have also included some cited examples from our group and others that show this variation.[4,19,20]

R stands for "resistance." This information is presented in the mechanical properties section.

pp. 4 :show conventional {112}<111> nanotwins…
pp. 5: Although it is unclear whether the crystal collapsed to ω first to aid twin nucleation second, or if the local shear stress from the twins triggered the ω transformation Fig. 2(c)
Did the authors observe any {332}(113) Twin as observed in β-Ti alloys? Also, please provide any physical background why {112}<111> Twin is more likely to form in this alloy system.

We thought we would observe {332}(113) twinning and so we very extensively looked for it but could not find any. We believe this twinning system is not activated in this alloy because it does not contain enough group IV elements. We have stated that we did not observe other twinning systems in the main text:

"We did not observe any other twinning systems."

{112}<111> twinning is the conventional twinning system in *bcc* elements and alloys. It is generally promoted by alloying. Other twinning systems become active when the stability of the *bcc* phase is low (such as {332}(113) twinning in β-Ti alloys).

pp.4: Our investigation began by analyzing the sequence of tensile deformation mechanisms. Tensile specimens, one interrupted shortly after yielding ($\varepsilon_p=0.004, \sigma_e \approx 1000$ MPa), and a second at the uniform elongation (($\varepsilon_p=0.051, \sigma_e \approx 1150$ MPa) were jet polished and examined in a transmission electron microscope (TEM).
Please clarify what you mean by ε_p and σ_e. If ε_p refers to plastic strain, please explain how you measured it.
In "At higher stresses, specifically at the ultimate tensile stress of 1150 MPa (which corresponds to 5.1% engineering plastic strain)," it is clearly stated that this is plastic strain. The expression should be consistent.

We understand how this is confusing. We measured the plastic strain by subtracting out the elastic contribution (i.e. the strain at yielding). We have changed all the stress and strains to true stress/strain.

pp. 5: bowing into mixed orientations from pinning-depinning events
Please specify whether the pinning-depinning event is due to the presence of twins or to the solid solution states in HEA?

While difficult to precisely determine what is causing all the pinning, we do note that some of it is from dislocation loops, as can be seen most clearly in some additional TEM we performed and included as fig. 3(c). Other probably reasons include the formation of an edge jog from the intersection of a dislocation with a different Burgers vector.

pp. 5: with a nanometer-thick interfacial layer of ω that expands in the space between the twins.
Based on the analogy from βTi, ω phase is expected to be formed in the region with high concentration of Gr. IV elements. Is it possible to analyze the elemental distribution in the region where ω phase and Twin are formed? Is the APT observation shown in Extended Fig. 14 and 15 actually related to ω phase formation?
Is it correct that unlike the TRIP effect, the ω phase itself does not play some role in plastic deformation?

The reviewer brings up an interesting point. We have analyzed the elemental concentration at the point on the boundary where the twin and omega formed and found an increase in group IV elements at that precise location. We have clarified this in the discussion section of the manuscript and the supplementary information figure:

"Atom probe tomography (APT) analysis of grain boundaries in Extended Fig. 14 shows that Ti and Hf enrich the boundaries in a ~3 nm range, raising the group IV concentration to 42 at.% in the measured region. A TEM energy dispersive X-ray (EDX) analysis (Extended Fig. 15) of the grain boundary from Fig. 4 at the twin/omega impact site indicates this boundary is also enriched in Ti and Hf, thereby producing an additional chemical driving force for stress-induced ω formation."

pp. 5: At higher stresses, specifically at the ultimate tensile stress of 1150 MPa (which corresponds to 5.1% engineering plastic strain), bulk dislocation glide is activated.
Is the dislocation activation discussed here due to dislocation formation from a stress concentration source due to the twinning mentioned at the beginning, or is it due to kink formation of the pre-dislocation?

There are invariably two sources of dislocations. The first is that of pre-existing dislocations in the material. This is a well-documented phenomenon in *bcc* materials. The second source of dislocations is the twin-interface sites. To provide documentation of these sites in uniaxial tension, we performed additional TEM on tensile samples that had been yielded. These additional experiments are now included in fig. 3. We found that if we followed twins to their termination sites, they generate stress concentrations high enough to nucleate very high densities of dislocations that glide ~2.5 µm into the material (into the adjacent and parent grain as well as along the twin boundary). We believe that these dislocations continue to glide away from the impact site at increasingly higher strains to mediate plastic flow.

pp. 6: There also exists a marked stress gradient.
What is the effect of stress gradients on the activity of plastic deformation modes?

The stress gradient at a crack tip causes the mechanisms to activate spatially rather than temporally (as they do in tensile samples). We have changed the statement to reflect this:

"Here, it is important to note that in the vicinity of a crack there are three principal stresses that are each approximately three times higher than those in tensile specimens which generates a hydrostatic stress, suppresses shear stresses and additionally produces a stress gradient."

pp. 6: These kink bands are formed by a_0⁄2<111> dislocations with a {110} glide plane
I understand the idea of earning plastic deformation by kink bands. I guess that the formation of kink bands by blade dislocations is accompanied by crack slowing at the crack tip. I would appreciate a detailed discussion of whether this means that blade dislocations are easier to form specifically in this alloy system.

The reviewer brings up an interesting point. There are two important distinctions to make here: does the material have a unique source for blade dislocations? *or* do the blade dislocations have a unique property that allows them to form kink bands?

The answer is that it is a bit of both. First, we observe that twins are a source of edge dislocations in these materials. Second, our HRSTEM studies of kink band boundaries indicate that the structure of the dislocations in the boundaries is very complicated. The pure edge dislocations will decompose in the boundary into pairs of mixed dislocations with <001> and <110> Burgers vectors, which stabilize and immobilize the boundary. Our corresponding MD simulations of these boundaries show that edge dislocations in this concentrated alloy are significantly more easily trapped in a kink band boundary than in a pure element because they are rugged and wavy. As they sluggishly glide through the material, they will easily interact with others and get trapped in the boundary. In pure elements, on the other hand, the edge dislocations are perfectly straight and move quickly. Their low glide stress easily destabilizes the boundary when there is

an applied global stress. We are preparing these experiments and simulations for a separate publication.

In summary, the twins are a unique source of edge dislocations. When twinning activates before dislocation glide, it impinges on boundaries and produces a unique stress concentration that promotes nucleation of multiple character dislocations. Moreover, the edge dislocations are not quite "blade" like; they are wavy and sluggish, which makes it easier for them to interact.

pp. 7: This is especially problematic since as a low symmetry hexagonal phase incapable of plastic deformation, ω can also act as a site for crack nucleation.
I would expect that the formation of the ω phase would prevent the stress concentration~fracture initiation, corresponding to the ease of nucleation of dislocations from such a stress concentration source. Is this true? Is it possible to demonstrate this based on the fundamental material properties in this alloy system?

This is an important caveat about the omega phase (or any brittle phase an alloy can TRIP to). Yes, it is true that omega is a form of plastic deformation that delays brittle fracture. However, the omega phase is in itself brittle. In a tensile test, it may take a large volume fraction of omega to see this effect occur, but in a fracture test, the high hydrostatic stresses cause the omega to fracture and crack much sooner. We have included a discussion of the hydrostatic stress in the Discussion section:

"However, these elements can act as "a double-edged sword" by promoting the transformation to omega (particularly at low temperatures), which is brittle in large amounts.[11,12] This is especially problematic for samples containing cracks because the high hydrostatic stress can promote stress-controlled transformations and exploit the brittleness of the new phase to accelerate fracture.[13,14]"

This process is not so different from the effects seen in materials with the TRIP effect. For instance, at the same temperature *fcc* CrCoNi transforms to the *hcp* ε martensite phase which delays fracture only in small quantity by elevating strain hardening.[21] However, if too much martensite phase formed then it would transform too much of the material into a brittle phase by which the crack could easily propagate.

pp. 9: show that the constraint imposed by stress-triaxiality prevents crack-tip stresses from reaching the screw glide threshold, limiting crack-tip plasticity to twins and edge dislocations that redistribute twinning-induced damage.
I believe that the effect of stress distribution at the crack tip on dislocation and twin formation is a universally important discussion that does not depend on the material. I would like the authors

to describe what stress components in the triaxial stress state would bring about the above features.

The reviewer brings up an interesting point about the stress triaxiality acting as a constraint on plastic deformation. It may seem initially counterintuitive, but increasing the stress, as well as adding stress tensor components does not necessarily enhance plasticity; in fact, the latter action suppresses it. It is this principle that is responsible for the fact that the fracture toughness in all materials decreases as a function of sample thickness until full plane strain conditions are met. In all materials that have a crack under an external tensile opening stress, there always exists a competition between brittle fracture and ductile dislocation emission. Whereas the brittle fracture is a function of the hydrostatic stress, the emission of the dislocation is purely a function of the shear stress. Under plane stress conditions, the stress state is biaxial, and the third principal stress is zero. Adding an out-of-plane tensile component to the stress tensor, σ_z, by making the material thicker (i) creates a small out-of-plane shear stress and (ii) decreases the in-plane shear stress. This is visualized by a nested Mohr's circle whose diameter shrinks as it is shifted to the right, such that all principal stresses are positive. For this reason, once plane strain conditions are met, the opening stress is enormous: on the order of 3-5 times the global tensile stress; but, the shear stress is diminished.

Materials with low yield strengths have low dislocation shear glide stresses, which cause plasticity to be activated before brittle fracture can occur. In materials with high yield stress, the shear stress to move a dislocation may be higher than the tensile fracture stress, and so brittle fracture ensues.

In this alloy, the twin shear stresses are lower than the dislocation glide stresses. In a tensile sample with no stress-imposed constraint, the plastic deformation runs its full course: starting with twinning and the phase transformation and proceeding to dislocation glide. However, in a sample with a crack, which imposes a triaxial stress constraint, the hydrostatic stress component will exploit brittle features in the microstructure, maybe even before screw dislocation glide stresses are reached. This is the basic principle governing DBTTs in *bcc* materials. But here, the fracture stress sits in between the twinning stress and the dislocation glide stress. Moreover, twinning stresses are not sensitive to temperature and so there is **no** temperature at which a truly brittle cleavage fracture can occur at least down to 20 K.

This generates a **universal** condition in materials with an inverted TWIP effect. If they can activate twinning first, *i.e.,* if the alloy is designed to have anomalously low twinning stresses, it will **not** pass through a traditional DBTT (given that these twinning stresses are low!). There will come a temperature below which twinning will control yielding and therefore crack-tip blunting. Since twinning stresses are largely temperature insensitive (and if they are aided by a phase transformation as we see here), then a fully brittle fracture may never occur.

pp. 12: J-integral was calculated as a sum of the elastic, Jel, and plastic, Jpl, strain energy contributions, Eq (6)
I believe that estimating the J-integral from experiments can lead to important information. I have not been able to fully follow the derivation of the plastic component of the J-integral in equation (6), but please mention how accurately it can be estimated.

The plastic component of the *J*-integral is obtained from the Merkle-Corten analysis.[22] We have cited this paper in the manuscript. It is a very accurate way to measure the plastic component of the *J*-integral. It simply converts the area under the curve into an energy measurement given the local tensile and bending moment at each instantaneous crack length.

Extended Figure 11.
The caption ends with an incomplete sentence.

We thank the reviewer for pointing this out. We did not intend for there to be a sentence there; it has been removed.

Overall comments:
There is no doubt that plastic deformation mechanisms other than dislocations are important for ductility and toughness. On the other hand, dislocations have the important feature that plastic deformation can be continued by Flank-Read sources (especially in FCC) and other growth mechanisms. How much plastic deformation can be induced in twinning and phase transformation will be the key to improving mechanical properties by utilizing other deformation modes. Therefore, I would appreciate it if you could mention the plastic deformation capacity and its limit that the twinning and ω transformation in this study can produce.
Also, the authors discussed that twinning and phase transformation occur in the initial stage of plastic deformation. Is it possible to specify the relationship of critical stresses between twin formation and phase transformation?

We believe that because the two mechanisms are complementary, *i.e.*, one cannot occur without the other, that the twinning stresses and transformation stresses are nominally the same. While there are likely minute differences, which is why we cannot claim which mechanism occurs first, it is apparent that when omega collapses between the twins, it occurs between the twinning-induced stress drop and the point at which we interrupted the tensile test.

One additional point to note here is that twins are produced by successive displacement of atoms along <111>, whereas omega is produced by alternating displacements along <111>. Therefore, as the twins migrate, the atoms between the matrix and the twin shuffle instead to form omega.[12]

This similarity in formation mechanisms could also drive similar stresses for twin and omega formation.

The alloy Nb45Ta25Ti15Hf15 is composed of Group IV and Group V. Recent research has helped us understand the role of Group IV, but we are very interested in how the detailed composition of 45-25-15-15 was designed. Is it an optimal design for mechanical properties such as plastic deformation capacity and toughness? I know that it is difficult to investigate on an ad hoc basis due to the large degrees of freedom, but could you please add any data you have on the effect of the concentration of each constituent element on these properties?

To address the point about composition we have added a Supplementary Note on the alloy design rationale. In summary, the 45/25 composition was chosen to maximize lattice distortion and solid solution strengthening but minimize the amount of Ta because this is a very dense element. The group IV composition was chosen to enhance ductility but was limited to 30% to keep the *bcc* phase stable. The 15-15 break-up was once again used to increase the lattice distortion. We used Hf instead of Zr because of Zr’s tendency to produce secondary, often detrimental, phases especially along the grain boundaries.

Referee #3 (Remarks to the Author):

Congrats on the nice and detailed work.

Topic is interesting and results are in part also interesting, however, I have a few concerns on the interpretation and explanations, as outlined below, specifically on the mechanism explanations that are given to explain this response.

We thank the reviewer for their detailed and thoughtful response. We have added additional experiments to the paper; specifically, we have investigated a simpler $Nb_{70}Ti_{30}$ alloy to identify the concentrated alloying effects and to see whether this mechanistic transition is specific to the multi-principal element alloy or whether it can be generalized to other systems. We have found that this transition also begins to occur in the binary alloy and that it prevents the toughness from decreasing with decreasing temperature down to 20 K. We hope that our additional experiments, edits, and answers to the questions below are satisfactory.

Divergent deformation pathways: not so clear what that means. See below, is a bit ill-defined. The interplay of slip and twinning is a very normal ting, also in BCC, or is s t else meant by that?

We have changed the title to "Inverted deformation sequenced inhibit ductile-brittle transitions in refractory alloys." We agree that the original title is vague.

Question when I see this composition: Hf and Ti – containing alloys are extremely prone to severe H embrittlement, due to dislo core structure changers and hydride formation, particularly at defects such as dislos and GBs. This should be very critically discussed I suppose (likewise, replacing Hf by Zr or more Ti in these types of BCC 'Senkov' taste alloys leads to the same type of environmental H- related embrittlement). For example, did the authors look at the response of samples under non idealized / real world conditions with e g some water content or so (the corrosive surface water splitting leads to ingress of atomic H in Hf-containing alloys). These aspects deserve attention, in order to not heal the DBTT problem from C, N, S etc but making the material instead vulnerable to H. (this is NOT to be confused with 'normal' H-embrittlement in BCC alloys / steels etc as these are no (localized) hydride formers).

The reviewer brings up a really interesting point about stress-corrosion cracking in structural materials. To address the issue of interstitial hydrogen in the alloy, we measured the concentration of hydrogen in our samples by APT. While APT does not give a very accurate measurement of the hydrogen and consistently overestimates the overall concentration. We have measured a concentration ~0.45 at.%, which is inflated by the measurement but is still

significantly lower than the concentration required (~1%) to trigger the *hcp* to *fcc* transition observed in Ti and Hf. Our tests at cryogenic temperatures (77 K) were performed in liquid nitrogen, and the materials showed a high fracture toughness. The experimental set-up of tests at 20 K (the ENGIN-X beamline) requires a vacuum to cool the samples. However, we believe that at such low temperatures the diffusion of hydrogen and other interstitials inside the metal would be very limited. Moreover, we believe that the stress-corrosion effects and hydrogen embrittlement caused by environmental exposure are outside the scope of this study which focuses on mechanistic sequences at quasi-static rates.

Not clear what is meant by 'multi-character dislocation glide' ? When rather clean, all these BCC alloys and BCC steels operate by 48 slip systems (see original papers by authors such as Christian and Sir Peter Hirsch e g). In FeSi it can be also twining plus 48 slip systems. In Ti – Nb / Ta / V etc it is dislo, twins and omega etc. - Is that meant here ?

We apologize for the miscommunication; our wording was unclear. The word "character" was given in reference to Burgers vector angles, not slip systems. We have removed this altogether and discussed the dislocations specifically as screw, edge or mixed.

Best check for a few statements: '…severely restricting their operating conditions..' is quite an overstatement, when considering the 1.7 billion tons of BCC steels produced and used every year… this is not quite underpinning such as statement as 'severely restricting their operating conditions' ; -) Also, this transition depends very strongly on C, N, S content etc as is well known, and modern BCC alloys do not really suffer from this limit, because they can be made very clean. Even artic region structures are since decades made of BCC steels, just that they are well controlled w r t cleanliness.. so better rewrite…

We agree that this is a bold statement and have removed the word "severely." We have instead just stated in the introduction:

"The expansive set of current and potential applications for *bcc* alloys, from building and transportation infrastructure to energy technologies, are often limited by this transition."

We have also added a Supplementary Note on the role of interstitials. We agree that low interstitial concentrations are critical for low DBTTs.

Omega phase forming BCC alloys are well studied in the Ti- Nb / Mo / V etc systems and local transformation to the omega phase is very well known – is that also observed in such simpler materials ? The fact that omega phase is formed does not require such as complex composition I guess. What is here the exact role than of this compelx composition ? Why this specific Nb / Ta ratio ? What is the rationale alloy design principle behind it ? How was this specific composition

derived ?

The reviewer raises a valid point regarding the alloy design, which we have addressed in a Supplemental Note. To summarize, we chose an alloy composition with multiple-principal elements because these systems have a much better synergy of strength, ductility and toughness over a much larger temperature range compared to binary alloys.[2,23,24] They can also activate a much larger set of deformation mechanisms across a wider temperature range. For example, at higher temperatures, the NbTaTiHf alloy can generate toughness through multi-angle character dislocation glide, kink-band induced softening, and TWIP effects. At low temperatures, it can maintain high toughness by changing the mechanisms that activate. This complexity and versatility in plastic modes are nearly absent in binary *bcc* Ti-alloys, where TWIP/TRIP effects are dominant, and the most controlled mechanisms are achieved by dilute or binary alloying.[25] For example, kink bands are rarely observed (unless at high strain rates).[26]

This specific composition was chosen for several applications. Initially, the composition was finely tuned to stabilize the *bcc* phase while also adding group IV elements to promote dislocation glide (through lowering of unstable stacking fault energies). This is the primary motivation (including the Nb/Ta concentration) that was chosen for the phase stability from the Calphad calculations that we performed, while minimizing the concentration of Ta, which is dense. Concerning cryogenic properties, we noticed that at 77 K this material had a significantly lower yield stress than what we predicted with MD simulations.[27] We also observed that the yield stress here was likely controlled by twinning, and so we wanted to know how this transition affected the lower temperature mechanical properties.

To understand the role of the complex composition, we made and performed additional tests on a $Nb_{70}Ti_{30}$ alloy. We found that this material passes through the same slip-twinning transition, and as a result, it also sees a plateau in strength and toughness. We note by TEM that the same mechanisms occur and that there is multi-angle character dislocation glide. The one notable difference, however, is that the strength is ~100 MPa lower and the UTS is nearly 200 MPa lower. This causes the fracture process to be strain-controlled rather than stress-controlled, and can elevate the toughness by another ~70 MPa√m. This indicates that while the high-entropy alloy has a much better combination of strength and toughness across a wide temperature range, the binary alloy can have higher toughness at low temperatures simply because plastic deformation by twinning, the transformation, and slip occurs at lower stresses. We have included some discussion about this in the discussion section:

"Although this sequence, one that begins with twinning/transformation and ends with dislocation glide, promotes tensile ductility, it is not initially clear why it does not run to completion to create a fully ductile fracture under the triaxial stress conditions present at a crack tip, particularly for $Nb_{45}Ta_{25}Ti_{15}Hf_{15}$ whose fracture surface has more brittle features. To

understand why inverted TWIP/TRIP is so sensitive to the stress-state, we obtained screw, edge, and twinning stresses and mapped them onto the HRR fields ahead of the crack tip. For $Nb_{45}Ta_{25}Ti_{15}Hf_{15}$, we obtained screw and edge dislocation tensile glide stresses using, respectively, the Rao-Suzuki model and the Maresca-Curtin model at 20 K with the experimental strain rate of $1 \cdot 10^{-3}\ s^{-1}$.[27–29] These values are listed in Table 1 for both alloys and mapped onto the HRR fields in Fig. 4(g)-(h). As can be seen in Fig. 4(g), whereas the local shear stresses at the crack-tip are high enough to activate twinning in $Nb_{45}Ta_{25}Ti_{15}Hf_{15}$ and $Nb_{70}Ti_{30}$, they are only high enough in $Nb_{70}Ti_{30}$ to activate dislocation glide within a characteristic distance for fracture. This indicates that the electronic structure of these alloys (primarily controlled by the group IV concentration) is responsible for this mechanistic transition, whereas chemical complexity simply raises the activation stresses—a property beneficial to the high temperature strength, but with a modest cost to the cryogenic toughness.[30,31]"

I do not quite understand this sentence: 'By inverting the sequence of deformation mechanisms typical in metals and alloys, tensile yielding and crack-tip blunting are controlled by temperature-insensitive defects that generate dislocation sources at interfaces where they intersect and elevate the local stress.' Inverting what ? dislos are active from the first moment, already in the microplastic regime in BCC. Dislos in BCC are for sure NOT temperature-insensitive mechanisms, but due to their high Peierls barriers they are actually much more temp-sensitive than FCC dislos. Maybe check this sentence – I could not really understand what the message is here. Maybe I misunderstood it?

We agree that this is poorly phrased and causes confusion. We have edited this sentence to refer to the sequence of mechanisms that cause TRIP and TWIP effects to arise: dislocations glide first, and form stress concentrations that then trigger twinning and/or transformations. Here, we have observed a reversed sequence where twins and a transformation come first, and then form stress concentrations that nucleate dislocations. It means that dislocation glide and twinning are "co-active" but twinning dominates; otherwise, the yield stress would rise considerably.

Statement 'This process keeps…' there was not really a process explained ? Also, any defect activity in BCC is very rate dependent – so that it would be interesting to see at which specific rates these statements holds and where they break down.

Please see above response; we have rewritten this paragraph.

We would like to perform high strain-rate experiments, but this is out of scope of the present study. We are emphasizing the quasi-static mechanical properties here.

Statement is not quite correct: '…from the temperature sensitivity of screw dislocations which are significantly less mobile 50 than their edge counterparts, and rely on the thermally activated

double-kink nucleation process..' What the authors mean likely is NOT formation of double kinks- this would mean two consecutive kinks of the same sign – BUT instead the likely refer to a kink PAIR, i.e. a plus and a minus kink (this the motion nucleus), which in themselves are actually + / - edge dislocations, and then the role of the secondary Peierls potential sets in. Better check such statements for correctness.

We thank the reviewer for pointing out this error. We are referring to kink pairs and have made corrections for this statement, and another later in the paper.

Statement: 'Consequently, facilitating screw dislocation glide through careful substitutional and interstitial alloying has been the governing principle to suppress the DBTT..' not quite sure if this is correct, because at low temperatures / higher rates the barrier for double cross slip plus FR source formation is the real barrier to replenish the dislo flux and comply with the imposed BCs, not so much the individual dislo motion, which actually depends a bot more on the segment length distribution. Sources always need the double cross slip, otherwise the BCC alloys undergo dislo starvation.

We agree that this sentence is vague. What we meant to say is that DBTTs are engineered to be lower in materials through careful alloying processes that either remove interstitials, which pin dislocations, or the addition of substitutional elements that soften the material (*e.g.*, Re in W, Ti in Nb, etc.). We have edited the sentence to reflect this. It is also included as part of an edit for a previous query:

"*Bcc* alloys are inhibited at low temperature by their crystal structure, which forces dislocations, specifically screws, to rely on thermally activated kink-pair nucleation to move.[32,33] Consequently, screw dislocation glide becomes sluggish at low temperatures, thereby causing strength to increase precipitously and plasticity to be constricted at the tip of cracks until fully brittle failure ensues.[34] Indeed, traditional strategies to suppress the DBTT are primarily governed by facilitating screw dislocation glide through careful substitutional and interstitial alloying.[1,2][15,35–37] While interstitial elements raise DBTTs by creating additional pinning stresses, substitutional alloying in some cases can soften *bcc* materials.[15,35–37] This has been observed in refractory binary and multi-principal element alloys (RMPEAs), that are often extremely brittle.[15,38,39] Here, concentrated additions of group IV (Ti, Zr, Hf) into group V (V, Nb, Ta) elements enhances screw dislocation glide by lowering unstable stacking fault energies, and raising the kink-pair equilibrium concentration.[15,38,40,41]"

The loading rates are essential in this field. When I make tests ever and ever slower, I see such behavioral trends also – have the authors tested the results for several loading rates ?

We have been consistent and used the same strain rate of 10^{-3} which is recommended in ASTM E8 for quasi-static loading conditions.[42] It is well known that with increasing strain rate, strength increases and ductility decreases in *bcc* materials. Studies of other refractory high-entropy alloys show that this is also the case for them.[43] We believe that understanding the role of strain rate on the behavior of these alloys is important and it is part of ongoing research projects in our group. This project includes investigation of dynamic properties of this material at multiple strain rates, including significantly higher strain rates, which is useful for impact resistance and spall strength measurements. However, we believe that these investigations are out of scope of the current study which explicitly focuses on quasi-static testing. We have investigated the properties holistically from this perspective: looking at tensile, *J*-integral fracture tests, and fracture surfaces, which provides a comprehensive measurement of DBTT.

Another boundary condition that is really essential in the field of measuring the ductile to brittle transition temperatures is the dwell time. This means that repeating the same tests after different waiting times gives completely different results, as particularly the literature on the refractory body centered cubic metals has often shown. Which is the reason? That many of these temperatures are plotted. Both as a function of time and function of purity, of course. So it might be interesting to repeat some of these tests after different dwell times And see if any trends are emerging similar as well known for. Binary refractory bodies in the cubic metals, for instance.

We agree that dwell time has a deleterious effect not only on the DBTT in *bcc* materials, but also on the ductility of *fcc* and *hcp* materials, as well as the fatigue life of all alloys. However, these tests are generally done for specific industry applications, mostly concerning fatigue, and we believe are out of scope of the present study.

For many body centered cubic steels, it was shown that the grain size plays a very important role for the. Transition point. And hence the question whether the authors looked into this dependence here.

We agree that grain size plays an important role here and it is one that we are very interested in investigating. However, producing a fine-grained *bcc* RHEA large enough to perform fracture tests is very challenging, particularly given the need for full recrystallization of the material. Our samples at room temperature must be nearly 14 mm thick to meet validity criterion of fracture tests according to ASTM E1820 (we did not even meet it at 8 mm thick with our current samples). Smaller thicknesses are possible for the lower temperatures because the strength increases to nearly 1 GPa, we would be unable to extrapolate data to higher temperatures and therefore measure any meaningful DBTT. While this is possible to achieve with conventional materials, it is not possible with conventional manufacturing techniques for refractories. We have considered additive manufacturing, but this is an ongoing project, where we will have multiple other compounding effects concerning texture, inclusions, contamination, etc to deal with. Due

to the similarity of this question with a more scientifically detailed following one, we have answered some scientific points about the effects of grain size in-depth below. To address the limitations of our study in relation to the grain size we have added a Supplementary Note. We do show that grain size does not affect this transition in another Cr-Mo alloy system.[9]

This statement about the underlying material design principle is not entirely clear w r t to the DBTT quest: 'alloying group IV into group V elements enhances screw dislocation glide by lowering unstable stacking fault energies. This combination of elements also reduces phonon frequencies, induces high lattice distortion, and when combined with high variations in local chemical environments intrinsic to RHEAs, increases the equilibrium concentration of double-kinks on screw dislocations that eliminates the need to thermally activate them'
The change in unstable stacking fault energies should be quite small, hence why is this a reason for the observed phenomenon ? Why should this promote kink PAIR formation ? (again: pls correct: these are not double kinks, they do not really help much here).
Why is it helpful is this alloy mix reduces phonon frequencies? I had assumed the opposite, i.e. that phonon are kionks little helpers, ie in BCC we want more of them, not less ? Is a bit counterintuitive or do I overlook s t here ?
Statement: 'induces high lattice distortion': given that we talk about BCC here these elements do not cause 'high' lattice distortion, all distorions stemming from interstitials in BCC are much much higher, but these do not help but instead counteract DBTT reduction. These is s t not quite lining up in this discussion. Also Cr or Ni etc in BCC Steels have also rather high distortions.
The statement 'high variations in local chemical environments intrinsic' is also not quite clear because BCC Fe Cr (Mn Si Al) C steels (and many others) have also very complex local chem landscapes, this reads maybe a little bit handwaving and less like a clear thermodynamic alloy design approach. Maybe these points need to be explained and justified and proven in more detail to leverage this response ? Reads otherwise scientifically not quite so clear.

There are several questions couched in here, and so we will answer them sequentially:

1. Unstable stacking fault energies: The unstable stacking fault energies (USFEs) of this alloy on all potential glide planes—{110}, {112}, and {123}—are significantly lower than for even Nb. For example, the USFE of Nb on {110}, {112}, and {123} is 647, 753, and 757 mJ/m^2, respectively.[15] However, for NbTaTiHf these values drop to 469, 480, and 483 mJ/m^2.[15] The lowering of USF energies also lowers the kink-pair formation energy by the Peierls-Nabarro framework.[44,45]
2. Phonon frequencies: Lowering phonon frequences aids in kink-pair formation (the amount of phonons here is irrelevant as this quantity is only a product of the number of atoms and dimensions). If we consider dislocations in the line tension model, then lowering the elastic wave frequencies may promote interactions between kink pairs and these waves.[46]

3. Lattice distortion: While it is true that interstitials in dilute alloys and in pure *bcc* elements induce significant distortion, in NbTaTiHf-based alloys, this distortion is less than that produced by the complex alloying in the lattice. We have calculated what these distortions are, and they are published in the literature. In this alloy, we calculated the lattice distortion from the compositional complexity to be an average 0.13 Å, with a minimum and maximum of 0.11 and 0.16 Å, respectively, for 100 SQS.[12] The distortion from oxygen interstitials is 0.10 Å on the long axis and -0.05 Å along the short axis for 100 SQS.[37] Therefore, the minimum lattice distortion due to chemical complexity is larger than the largest distortion produced by interstitials.
4. "High variations in local chemical environment": We agree that it is not well defined between alloys and have removed this part.

The statement that all these effects reduce edge dislocation mobilities is not so clear. The edges are not really relevant as the authors stated above (excepts for those that move with near sound speed along the Peierls ridges). Not so sure about the chain of thought here.

Our experimental evidence (Figs. 3 and 4) indicates that edges are significant for the creation of kink bands, but these bands do not play a very important role in the deformation as compared to higher temperatures. We have removed this discussion, especially since our experiments shows that Nb70Ti30 is also ductile, and here the edges are way more mobile (see Table 1).

Likewise, the claimed decrease in twinning stresses and promotion of the transformation to the omega phase at cryogenic temperature is well know for BCC-Ti/Zr/Hf alloys and is usually well known to very embrittling. This seems to contradict the literature on the role of omega ?

We also agree that omega is embrittling. We have added a clarifying statement in the discussion section outlining how the hydrostatic stresses at a crack tip can promote fracture. Our concluding paragraph discusses the balancing act of group IV elements (increasing them promotes dislocation glide but also increases the driving force to produce omega. We also discuss this in the results section. Our key observation here is that we only observe very small amounts of omega. We do not observe any regions of the crystal transformed to omega that are larger than what we show in Fig. 2 where omega is seen between two twins and is ~50 nm wide at its largest. We also do not observe the crack connecting between regions that have transformed to omega, as occurs in triaxial stress fields in these alloys (and any alloy with a critically large enough volume of a second brittle phase):

"Previous studies have shown the group IV elements promote twinning and slip, processes necessary for inhibiting the DBTT.[2,15,39,41] However, these elements can act as "a double-edged sword" by promoting the transformation to omega (particularly at low temperatures), which is brittle in large amounts.[11,12] This is especially problematic for samples containing cracks because

the high hydrostatic stress can promote stress-controlled transformations and exploit the brittleness of the new phase to accelerate fracture.[13,14]"

Yes, agree: the competition between dislocation glide stresses and twinning stresses that is important (but it is not completely temperature insensitive) but I had assumed that the dislo stress remains ALWYS below that for twinning in such alloys – this depends BTW very highly on grain size – was that checked here ? When GS goes down, dislo nucleation becomes hard and twins take over – this migth be essential here – however this is NOT due to the screw mobility in BCC, BUT due to the missing / insufficient bow out length required for FR / double cross slip and FR formation vs twinning out if the GBs. So this response should be highly GS dependent.

We thank the reviewer for pointing out this phenomenon. We have added statements about microstructural contributions to toughness in the discussion. It is a well-documented observation that twinning stresses can become lower than that of dislocation glide stresses. This occurs in Mo, Cr, W, and even in Nb at these temperatures. It also occurs in MoWRe.[47] However, the one caveat is that these properties are measured in compression. We have stressed this point in the Introduction. What is novel about our findings is that we observe this transition in tension and that it is nominally not embrittling, as has been documented before.

We agree that grain size influences ductility and toughness, and as the grain size decreases, twinning is promoted. Given that our grain sizes are coarse, we would expect this effect to become more pronounced with decreasing grain size. Our observation that it is not detrimental to the properties would suggest that decreasing grain size would not have an appreciable effect, as has been observed in a Cr-Mo system.[9] We have added statements to the Discussion and Supplementary Materials to emphasize that this is an important factor in the toughness (one that our micromechanics model is predicated on, as are other similar micromechanical models), and that our inability to test different grain sizes is a limitation of this study.[20]

Statement: 'The bcc RHEA also contains interstitial elements: 0.059 at. % (60 ppm) C, 83 0.0495 at.% (58 ppm) N, and 0.146 at. % (197 ppm) O. Is the H content known ? This is game changer when Ti and Hf are used. Otherwise tis material of course super clean, where actually most BCCs have very low DBTTs.

As stated in a previous answer, we were able to get an upper-bound estimate of the H concentration with APT. It is 0.45 at. %. This is, of course, an overestimate of the amount that is in the material, but it is still too low to induce any phase changes in Ti and Hf. We agree that clean *bcc* materials have low DBTTs. We have added a supplementary note about microstructural conditions to our paper. However, even clean *bcc* materials rarely have DBTTs as low as this alloy, and their strengths still increase dramatically. Nb may be ductile, but it is much more sensitive to impurities compared to its alloys with group IV elements.[37] One reason why it is

believed that the fact that some refractories (Nb, for example) have no DBTTs is due to literature that tested single crystals in compression (which, of course, produces significant plasticity).[32] Tests done in tension and especially in fracture (which is a rigorous method to test the DBTT) show that the mechanical behavior diverges.[38,40]

Fig 1: better also always show the derivative of stress stran curves in addition, otherwise hard to see mechanism transitions.

We have included the instantaneous strain hardening curves in the main text.

Strain rate was very low, was any higher strain rate tested ? see explanations above.

Our tests were performed under quasi-static conditions (and under one of the highest possible strain rates to meet these conditions). These tests are performed to keep the fracture and tensile properties self-consistent. We agree that increasing the strain rate would raise the DBTT, but analysis of the effects of strain rate is out of scope of the present study. Our combination of tests is a very holistic and standard way of testing the DBTT.

Statement 'twinning overactivates and delaminates the grain boundary' not so clear. The GB is the start point / and sometimes also the end point of a twin, why is a twin delaminating a GB ? not so clear.

We agree that this is not so clear and have edited the sentence to reflect the fact that our experiments cannot ascertain whether the twin impinges on the boundary to break it, or that it originates from the boundary that it breaks.

I understand from the paper that all the dislocation characterization that is presented in Figs 2 and 4 is post deformation. This means that all the screw dislocations are relaxed back to their energy valleys. So no direct proof is given for the claims of the change in the activation mechanism change that was discussed before. In other words, the statistics shown in the paper is quite nice, however, it is not really underpinning what is claimed as the underlying reason for this change in the DBTT. Seems to be quite pertinent to make a closer connection between these dislocation observations and the actual mechanism claims (see above, SFaults, phonons, kink populations under load etc.). This link is missing. Dislocation analysis in such BCC materials typically is done under load and not after relaxation when aiming to show changes in the kink and double crosslip behaviour.

The reviewer raises a really important point about dislocation characterization. We are not aiming to show changes in the kink and cross-slip behavior, simply what the general angle-character of the dislocations is. We understand that this can be affected by relaxation; however, it

is not possible to relate dislocation behavior obtained from *in situ* TEM foils to bulk tensile/fracture samples. This is because of small-scale effects and the large differences in the stress-state. We do agree that this method would be essential to characterizing kink populations, and it would be good to draw experimental evidence for some of these other variables. However, there is ample current experimental data (summarized in this theoretical paper)[15] that demonstrates the correlation between ductility and these parameters.

We are presently conducting extensive TEM ptychography studies of dislocation cores and find that the dislocations are highly kinked and wavy. We are preparing this for a separate publication.

One essential point that is still puzzling me after reading the paper is why the composition has to be so very complex and why the same main trade off question, namely between twinning and dislocation kink activity cannot also be achieved by much simpler alloys. This point has not really been explained very well. This point relates a little bit to the other comments above, where the rationality behind this specific alloy composition has not been very well explained. What is the alloy design sweet spot that requires this specific alloy composition? What is the main physics handle here? That was left a little bit unanswered. Why does it not work on Fe Si or Ti Nb etc (where dislos, twins and omaga etc are long known to interact.). Maybe it would be helpful to plot corresponding reference data on such a simpler alloy also.

We agree with the reviewer's assessment that it is not clear whether these effects would also occur in simpler binary alloys. To address this question, we performed the same experiments on a chemically simpler, but electronically similar $Nb_{70}Ti_{30}$ alloy and found that the same transition occurs here as well. Our results indicate that the chemical complexity is not as important a variable as the group IV concentration. In fact, the chemical complexity primarily serves to elevate the yield stress compared to the binary alloy. This is helpful for the high-temperature strength of this alloy, especially in comparison to the binary $Nb_{70}Ti_{30}$ material,[24] but it causes the yield strength to be higher at low temperatures, which can promote a more stress-controlled fracture, more brittle features, and slightly lower toughness. Our TEM results also indicate that dislocation glide is easily promoted in this alloy and that the twins nucleate them when they terminate against an interface.

1. Sahragard-Monfared, G. *et al.* Tensile creep behavior of the Nb45Ta25Ti15Hf15 refractory high entropy alloy. *Acta Materialia* **272**, 119940 (2024).
2. Cook, D. H. *et al.* Kink bands promote exceptional fracture resistance in a NbTaTiHf refractory medium-entropy alloy. *Science* **384**, 178–184 (2024).
3. Zhang, C. *et al.* Strong and ductile refractory high-entropy alloys with super formability. *Acta Materialia* **245**, 118602 (2023).

4. Bonnekoh, C. *et al.* The brittle-to-ductile transition in cold-rolled tungsten sheets: Contributions of grain and subgrain boundaries to the enhanced ductility after pre-deformation. *Nuclear Materials and Energy* **25**, 100769 (2020).
5. Yoshida, S., Ohba, Y. & Nagata, N. The Effect of Prestraining on the Ductility of Pure Chromium. *Transactions of the Japan Institute of Metals* **1**, 49–53 (1960).
6. Read, D. T. Metallurgical effects in niobium-titanium alloys. *Cryogenics* **18**, 579–584 (1978).
7. An, Y. *et al.* Thermally Stable Heterogeneous-Grained Refractory High-Entropy Alloy Offering Superior Strength from 77 to 973 K. *Nano Lett.* **25**, 15785–15792 (2025).
8. Sleeswyk, A. W. ½<111> screw dislocations and the nucleation of 112<111> twins in the b.c.c. lattice. *The Philosophical Magazine: A Journal of Theoretical Experimental and Applied Physics* **8**, 1467–1486 (1963).
9. Hinrichs, F. *et al.* A ductile chromium–molybdenum alloy resistant to high-temperature oxidation. *Nature* **646**, 331–337 (2025).
10. Anand, A. & Singh, C. V. Origin of gamma surface asymmetries in body-centered cubic refractory high entropy alloys. *Computational Materials Science* **233**, 112680 (2024).
11. Xing, H. & Sun, J. Mechanical twinning and omega transition by ⟨111⟩ {112} shear in a metastable β titanium alloy. *Applied Physics Letters* **93**, 031908 (2008).
12. Borges, P. P. P. O., Ritchie, R. O. & Asta, M. Local lattice distortions and the structural instabilities in bcc Nb–Ta–Ti–Hf high-entropy alloys: An ab initio computational study. *Acta Materialia* **262**, 119415 (2024).
13. Banerjee, D. & Williams, J. C. Perspectives on Titanium Science and Technology. *Acta Materialia* **61**, 844–879 (2013).
14. Ballor, J., Li, T., Prima, F., Boehlert, C. J. & Devaraj, A. A review of the metastable omega phase in beta titanium alloys: the phase transformation mechanisms and its effect on mechanical properties. *International Materials Reviews* **68**, 26–45 (2023).
15. Borges, P. P. P. O., Ritchie, R. O. & Asta, M. Electronic descriptors for dislocation deformation behavior and intrinsic ductility in bcc high-entropy alloys. *Science Advances* **10**, eadp7670 (2024).
16. Borges, P. P. P. O. Tunable energy landscape of screw dislocation cores by compositional fluctuations in bcc high-entropy alloys from first-principles calculations. *Phys. Rev. Mater.* **9**, (2025).
17. Xu, S., Su, Y., Smith, L. T. W. & Beyerlein, I. J. Frank-Read source operation in six body-centered cubic refractory metals. *Journal of the Mechanics and Physics of Solids* **141**, 104017 (2020).
18. Bienvenu, B., Dezerald, L., Rodney, D. & Clouet, E. *Ab initio* informed yield criterion across body-centered cubic transition metals. *Acta Materialia* **236**, 118098 (2022).
19. Gludovatz, B. *et al.* Exceptional damage-tolerance of a medium-entropy alloy CrCoNi at cryogenic temperatures. *Nature Communications* **7**, (2016).

20. Ritchie, R. O., Knott, J. F. & Rice, J. R. On the relationship between critical tensile stress and fracture toughness in mild steel. *Journal of the Mechanics and Physics of Solids* **21**, 395–410 (1973).
21. Liu, D. *et al.* Exceptional fracture toughness of CrCoNi-based medium- and high-entropy alloys at 20 kelvin. *Science* **378**, 978–983 (2022).
22. Merkle, J. G. & Corten, H. T. A J Integral Analysis for the Compact Specimen, Considering Axial Force as Well as Bending Effects. *J. Pressure Vessel Technol* **96**, 286–292 (1974).
23. Wang, S. *et al.* Mechanical instability and tensile properties of TiZrHfNbTa high entropy alloy at cryogenic temperatures. *Acta Materialia* **201**, 517–527 (2020).
24. Begley, R. T. & Bechtold, J. H. Effect of alloying on the mechanical properties of niobium. *Journal of the Less Common Metals* **3**, 1–12 (1961).
25. Ballor, J., Li, T., Prima, F., Boehlert, C. J. & Devaraj, A. A review of the metastable omega phase in beta titanium alloys: the phase transformation mechanisms and its effect on mechanical properties. *International Materials Reviews* **68**, 26–45 (2023).
26. Zheng, Y., Zeng, W., Wang, Y. & Zhang, S. Kink deformation in a beta titanium alloy at high strain rate. *Materials Science and Engineering: A* **702**, 218–224 (2017).
27. Wang, W. *et al.* A theoretical study of solid solution strengthening in the refractory medium entropy alloy Nb45Ta25Ti15Hf15. *Materials & Design* **261**, 115368 (2026).
28. Lee, C. *et al.* Strength can be controlled by edge dislocations in refractory high-entropy alloys. *Nat Commun* **12**, 5474 (2021).
29. Rao, S. I., Woodward, C., Akdim, B., Senkov, O. N. & Miracle, D. Theory of solid solution strengthening of BCC Chemically Complex Alloys. *Acta Materialia* **209**, 116758 (2021).
30. Maresca, F. & Curtin, W. A. Theory of screw dislocation strengthening in random BCC alloys from dilute to "High-Entropy" alloys. *Acta Materialia* **182**, 144–162 (2020).
31. Wang, F. *et al.* Multiplicity of dislocation pathways in a refractory multiprincipal element alloy. *Science* **370**, 95–101 (2020).
32. Christian, J. W. Some surprising features of the plastic deformation of body-centered cubic metals and alloys. *Metall Trans A* **14**, 1237–1256 (1983).
33. Dezerald, L., Proville, L., Ventelon, L., Willaime, F. & Rodney, D. First-principles prediction of kink-pair activation enthalpy on screw dislocations in bcc transition metals: V, Nb, Ta, Mo, W, and Fe. *Phys. Rev. B* **91**, 094105 (2015).
34. Ashby, M., Shercliff, H. & Cebon, D. Fracture and fracture toughness. in *Materials* 203–230 doi:10.1016/B978-0-08-102376-1.00008-1.
35. Wang, S. *et al.* The effects of interstitial oxygen on mechanical properties of TiZrNb medium-entropy alloy over a wide temperature range. *Journal of Alloys and Compounds* **989**, 174394 (2024).
36. Belcher, C. H., MacDonald, B. E., Apelian, D. & Lavernia, E. J. The role of interstitial constituents in refractory complex concentrated alloys. *Progress in Materials Science* **137**, 101140 (2023).

37. Borges, P. P. P. O., Ritchie, R. O. & Asta, M. Chemical trends favoring interstitial cluster formation in bcc high-entropy alloys from first-principles calculations. *Acta Materialia* **294**, 121091 (2025).
38. Kumar, P. *et al.* Degradation of the mechanical properties of NbMoTaW refractory high-entropy alloy in tension. *Acta Materialia* **279**, 120297 (2024).
39. Huang, H. *et al.* Achieving high tensile strength and ductility in refractory alloys by tuning electronic structure. *Nat. Mater.* 1–9 (2026) doi:10.1038/s41563-025-02464-8.
40. Kumar, P. *et al.* Compressive vs. tensile yield and fracture toughness behavior of a body-centered cubic refractory high-entropy superalloy Al0.5Nb1.25Ta1.25TiZr at temperatures from ambient to 1200°C. *Acta Materialia* **245**, 118620 (2023).
41. Tsuru, T. *et al.* Intrinsic factors responsible for brittle versus ductile nature of refractory high-entropy alloys. *Nat Commun* **15**, 1706 (2024).
42. ASTM E8: Standard Test Methods for Tension Testing of Metallic Materials. (2022) doi:10.1520/E0008_E0008M-22.
43. Wang, Z. *et al.* Strain rate sensitivity and dynamic constitutive response of a Zr42Ti15Nb20Ta20Al3 refractory high-entropy alloy. *Intermetallics* **189**, 109124 (2026).
44. Ngan, A. H. W. A generalized Peierls-Nabarro model for nonplanar screw dislocation cores. *Journal of the Mechanics and Physics of Solids* **45**, 903–921 (1997).
45. Joós, B. The Peierls Stress of Dislocations: An Analytic Formula. *Phys. Rev. Lett.* **78**, 266–269 (1997).
46. Eshelby, J. D. The interaction of kinks and elastic waves. *Proceedings of the Royal Society of London. Series A. Mathematical and Physical Sciences* **266**, 222–246 (1997).
47. Senkov, O. N., Rao, S. I., Balbus, G. H., Wheeler, R. & Payton, E. J. Microstructure and deformation behavior of MoReW in the temperature range from 25°C to 1500°C. *Materialia* **27**, 101688 (2023).

RESPONSE TO REVIEWERS' COMMENTS

Our response to the reviewers' comments is in blue text; changes to the manuscript are in red text, both here and in the actual manuscript.

Referee #1

Summary of the key results
(includes statements of my previous assessment) Body centered cubic (BCC) metals and alloys exhibit characteristic transitions from low to high ductility/toughness when surpassing a certain temperature – the brittle-to-ductile Transition temperature (BDTT). This type of embrittlement obtained at low temperatures is often limiting the engineering application scenarios of otherwise interesting candidate materials.
In the present manuscript, the authors provide a comprehensive analysis of the low temperature mechanical performance, incl. ductility and toughness, of concentrated solid solutions, i.e. Nb45Ta25Ti15Hf15 as well as Nb70Ti30. The data impressively shows that the alloys, in the microstructural condition investigated, exhibit reasonable ductility (not only strain to fracture but also uniform strain) as well as toughness even at 20 K. For most application scenarios, no relevant BDTT is thus obtained. It is specifically noteworthy that this is the case for both assessment types, i.e. ductility and toughness, which is not often provided at the same time in literature. Furthermore, the authors appended the investigation on the binary Nb-Ti alloy during the revision process proving that the findings are general and not related to the chemical complexity of the quaternary alloy. The microstructural and theoretical assessment of the finding reveals that an unusual combination of deformation mechanisms contributes to this behavior. In the present case, deformation twinning and phase transformation mediated plasticity prevent a temperature-dependent increase of strength at low temperatures. This behavior mitigates a strong constriction of the plastic zone at the crack tip.

B. Originality and significance: if not novel, please include reference
The findings are surprising and interesting to the field of physical metallurgy, the quality of the provided data is high, the assessment sound and statistically relevant. Thus, I recommend the manuscript for publication. The authors have addressed relevant aspects raised by the reviewers. Reviewers no. 2 and 3 have raised important points, for example regarding hydrogen embrittlement and strain rate impact. I agree that these are fundamental issues to be investigated. The authors convincingly outline that the present study is limited to fully exploiting these topics in the relevant depth at the moment. The limitation to quasistatic conditions is reasonable and an upper rough estimation for the H content was provided by APT to put the investigations into perspective.

C. Data & methodology: validity of approach, quality of data, quality of presentation
Minor stylistic aspects might be improved regarding the presentation:
- Please check the capitalization of the vertical axis descriptions in Figs. 1 and ext. 2.
- Please unify the true strain notation in the main text and the mechanical characterization section, ll. 358-361.

- I recommend introducing “electron _backscatter_ diffraction”, l. 456.
- There is a superfluous “that” in l. 512. Section “Micromechanical models of stress-controlled fracture for an inverted mechanistic sequence”, ll. 507-534, should be cross-checked once more regarding the text.
- There is a superfluous “deformation” in the caption of ext. Fig. 10.

D. Appropriate use of statistics and treatment of uncertainties
The data is statistically robust. One remark by reviewer no. 2 was addressed by referring to literature results as the material under investigation is currently limited in quantity. This is reasonable.

E. Conclusions: robustness, validity, reliability
The assessment and conclusions are reasonable and display an important finding for the community. As described above, there are open questions to be resolved (rate dependence, grain size dependence, hydrogen/interstitial impact) which seems reasonable given the importance of the discovery. I consider this work to be beyond the scope of the present article and I’m convinced that the community will take up the principle presented here.

F. Suggested improvements: experiments, data for possible revision
None.

G. References: appropriate credit to previous work?
This aspect has appropriately improved over the previous version.

H. Clarity and context: lucidity of abstract/summary, appropriateness of abstract, introduction and conclusions
This aspect has appropriately improved over the previous version.

Response:
At the outset, we would like to thank the reviewer for taking the time to read our revision, as well as our comments to points raised by the other reviewers. We have combed through the text and figures and made the typographic revisions.

Referee #2:

I appreciate the authors' sincere attitude in addressing the reviewer's comments, including additional TEM observations, and elemental characterization using APT and TEM-EDX. I confirm that the authors have provided clear and convincing responses to the questions raised by reviewers, based on their deep knowledge and insight into materials science and mechanics. The design strategy for the alloy system in this study is also very clearly presented and may serve as a useful guideline for the future design of HEA and solid solution alloys. The proposed deformation mechanism represents an excellent finding that could help overcome the intrinsic issue of low-temperature brittleness in conventional BCC alloys, and I look forward to its publication. I also expect

to see that the formation of dislocations within twins and the subsequent complex processes will be further elucidated through MD simulations using MLP.

* I have one final question. The authors' additional analyses have clarified that the segregation of Group IV elements at grain boundaries serves as the chemical driving force for the omega-phase transformation. While the authors have also explained the relationship between the omega phase and twins from an atomic-scale perspective, it remains unclear how the formed ω phase influences deformation governed by dislocation motion and twinning. In particular, how does the presence of the ω phase affect macroscopic plastic deformation behavior?

Although the coexistence of the ω phase and twins is frequently observed, there appears to be limited discussion on their combined impact on macroscopic plasticity. In the present alloy system, the omega phase forms due to compositional fluctuations, and its elastic constants might differ significantly from those of the matrix phase. While the authors have addressed its potential contribution to embrittlement, it would be very interesting to further understand the mechanisms by which such microstructures lead to fracture.

This point may also be addressed as part of the future outlook of this study.

Response:
We would like to firstly thank the reviewer for taking the time to read our revised manuscript and provide additional comments.

To address the first point about strain hardening, the omega phase as a transitional structure between the twin and the matrix, can change the hardening behavior. It is possible that due to elastic incompatibilities and differences in slip systems between omega and the *bcc* phase, as well as the volumetric contraction of the omega phase, it becomes more difficult for a dislocation to transmit through a twin boundary if that boundary has additional layers of omega. This could theoretically promote dislocation accumulation along these boundaries, or conversely, cross-slip along the boundary. This would require in-situ experiments to verify. Second, our observation that omega can expand in the space between twins indicates that this would indeed act a strain hardening mechanism by essentially blocking the “highways” created by the twins.

In reference to the second point, the reviewer correctly remarks that the segregation of group IV elements induces a driving force for the transformation of the bcc phase towards omega.[1] The reviewer also correctly remarks that omega transformation and twin formation are closely correlated deformation pathways in bcc materials, especially at low temperature and in alloys enriched with group IV elements.[2] In a recent computational study, collaborators in our group have further investigated the synergies between these deformation mechanisms in NbTaTiHf.[3] First-principles calculations have been performed to obtain unstable stacking fault energies in bulk bcc and omega phases, the interface energy between bcc and omega phases and twin boundary stacking fault energies. Results show that the formation of bcc-omega interfaces is energetically preferred over

the growth of the bulk omega phase. Further, calculations of the Rice parameter for intrinsic ductility show that omega phase formation is a potent embrittlement factor. As a result, the growth of the omega phase is challenged by its brittleness and the energetic preference for the formation of bcc-omega interfaces. These computational findings are consistent with the experimental reports in the present work, in which we have observed that the omega phase remains constrained to nanolayers within the bcc matrix. Twin formation is different. Specifically, the calculations show that the Rice parameter for twin boundaries does not represent an embrittlement factor, being more favorable compared to that found for the bulk bcc phase. In this sense, twins can grow to coarser length-scales, presenting a more favorable pathway for plastic deformation compared to omega formation.

To address these points, we have added additional statements in red to the discussion.

"Density Functional Theory (DFT) calculations at 0 K reveal that in NbTaTiHf alloys, increasing group IV concentrations promotes lattice instabilities from *bcc* to ω and that ω is predicted to be intrinsically brittle by the Rice parameter.[1,3]"

"As the applied stress increases, glide is progressively favored over twinning (~ 50 percent of the grains are in the anti-twinning orientation, they are expected to deform by screw dislocation glide with the original compact core). Dislocations with the lowest barriers to slip are selected—specifically those that can cross-slip along twins—channeling them along these boundaries. Omega as a twin transitional structure can further promote channeling by preventing dislocation transmission across twin boundaries. In regions where omega spans the distance between adjacent twins (Fig. 3(b)), dislocations can accumulate, similarly to material with pre-existing omega particles. While this hampers uniform ductility, it assists sustained necking, leaving the total fracture strain high and nominally elevating toughness.[4]"

Referee #3:

At the outset, we would like to thank the reviewer for carefully reading our paper and making insightful comments on our manuscript. We have combed through the text to revise some of the wording, adding additional clarifying statements, as well as citations. We hope that these changes address the reviewer's concerns.

See last comments about vague title…: not much better now ... The new title: 'Inverted deformation sequences inhibit ductile-brittle 2 transitions…' is likewise very unclear: 'Inverted deformation sequences': Not so sure if this makes things better, title is still pretty unclear. What exactly is inverted here? As stated in the last review cycle: sequence & interplay of mechanical twinning and dislocation activity is a very normal thing in many BCC alloys (see overview papers ch as that by Christian and Mahajan in 1995 (Progr in Materials Science) and the papers of Sleeswyk and Seeger etc. in which many alloys such as Fe, Fe Cr, Ti – Mo / V / Cr / Fe / Nb etc alloys and all the Fe Si alloys etc as well as many Mo alloys etc were reviewed w r t twinning and screw core

effects. There is however no such thing as a strict ‘inversion’ (?) or very strict sequence (see e g original single crystal papers from the 70s and 80s on BCC with twins). There is an immense temperature and rate dependence, orientation dependence, Schmid factor dependence etc - not sure if this ‘fancy’ terminology really aids or blurs the view here.

‘ductile-brittle 2 transitions…’ plural is by purpose – are the authors sure ? Figure 1 does not cover the use of a plural here ? Or did I miss s t here ? I see only one transition.

Response:
We have addressed this statement at the end of our response, as the word “inverted” is vague in several ways. We have removed this word where necessary and clarified it in other places. We have also made “transition” singular. The title is now: “competing deformation mechanisms inhibit the ductile-brittle transition in refractory alloys.”

We have chosen this phrasing because twinning and slip are competitive processes in *bcc* alloys, and we observe this competition happening in the investigated alloys.

‘catastrophic drop in toughness’: depends in BB very highly on alloy cleanliness. For very clean alloys it is not ‘catastrophic’.

We agree with the reviewer that the “suddenness” of the DBTT can vary as a function of composition. We have removed this word.

Are the authors really sure to claim that ‘plastic deformation begins with twinning’ ? This is practically impossible I guess…. You need at least a few partials to nucleate a BCC twin I suppose. Hence, no dislocations and spontaneous twins… ? not sure of this can be true. The activation stress of a wide dangling segment and it s double cross slip plus FR source is happening at very low stresses. I would tend to revisit such a claim and be rather very careful.

Unclear: twinning in BCC is not a ‘temperature insensitive defect’: very well documented e g for Fe Si and Fe C etc that twinning is temperature- and rate dependent. In general, I suggest that a bit more care is taken in considering the original literature on BCC twins and reflect more crisply, what exactly the novelty is compared to the know physics dating back to the 1950s.

We have included our response to this statement in the paragraphs at the end of the review.

The tendency of BCC metals and alloys to deform by mechanical twinning rather than

dislocation slip ALONE is a deeply temperature-sensitive phenomenon; mechanical twinning in BCC is thermally usually more suppressed at elevated temperatures, where mobile screw dislocations more easily accommodate plastic strain by slip, and progressively activated at lower temperatures, where screw dislocation mobility is frozen out and the locally elevated stresses drive twin nucleation. This understanding, rooted – as explained again in this paper - in due to the core structure of BCC screws, and remains the foundation for BCC design and DTB transition rationales till today. See e g the recent sunmary in https://pubs.aip.org/aip/jap/article/134/5/055102/2905122/Competition-between-full-slip-and-twinning-in-BCC and others.

I am still not satisfied wit the slightly handwaving discussion about the claimed inversion: early and quite direct demonstrations of the screw / twin and (later) twin / omega temperature dependence was e g given by Hull in 1961 (about the former) in tensile deformation experiments on Fe–3Si across a wide range of temperatures (from room temperature down to −253 °C !). He very clearly showed that as temperature dropped, twinning became increasingly prevalent at the expense of slip, providing one of the first quantitative datasets linking temperature to the slip-to-twin transition in a BCC system. Thornton and Mitchell also showed the same trend in 1962 for a broader class of alloys under cryogenic conditions, demonstrating that the competition between slip and twinning is a general feature of BCC low-temperature plasticity rather than a peculiarity; see eg https://www.sciencedirect.com/science/article/abs/pii/0001616061900694

Along similar lines, Bolling and Richman also looked at temperature-dependent twinning behavior in BCC in their paper series on continual mechanical twinning in Acta Metallurgica in 1965 and found there BCC alloys that deform almost exclusively by twinning over a wide temperature range, and they were able to isolate the flow behavior associated with twinning and characterize it independently of slip contributions. What is quite remarkable is that they found that in regimes dominated by twinning, the flow stress displayed a positive sensitivity to temperature and a negative sensitivity to strain rate — the exact inverse of what is observed for BCC slip, which is thermally activated. This anomalous behavior attracted considerable attention and helped motivate a more mechanistic understanding of why twinning in BCC metals behaves so differently from its FCC counterpart. Best look at their papers e g here https://www.sciencedirect.com/science/article/abs/pii/0001616065901367 to place ur work in to a better perspective of what is already state of the art in this field. (the mechanistic foundations are still subject to debates of course to some extend)

We have included our response to this point in the paragraphs at the end of the review.

Also, on the ‘sequence’ discussion (see critique above) I would also recommend to look a the excellent papers of Sleeswyk in 1962 (see your citation 51) and later in 1974, focusing on ½⟨111⟩ screw dislocations in the BCC lattice and connecting their tendency to adopt a non-planar, sessile core configuration at low temperatures to the nucleation barrier for twinning. The physical picture that emerged from these studies is that the same core structure responsible for the high Peierls stress of BCC screws — and thus for the steep temperature dependence of BCC yield strength — also controls the stress concentrations necessary to nucleate a twin. Some of these points overlap with some of the content of this paper, yet, it might help a bit to avoid any ‘overclaiming’ or sequential dislocation-twin pathways that are not likely to be true; as these two defects are very closely linked in their activation. I can only firmly underlin that these papers from the 1950s onwards looked in part in a very details manner at these links.
Also, Priestner and Leslie in 1965 modeled twin nucleation at slip-plane intersections and argued that the dramatic increase in yield stress at low temperatures generates the local stress concentrations required to operate a twinning mechanism. This is in my view a very likely connection to be considered in the argumentation here.
In other words, it is precisely because BCC screw dislocations become so immobile at low temperatures that the applied stress can reach the critical twinning stress before the material yields homogeneously by slip. The slip–twin transition is therefore not an arbitrary threshold but a natural consequence of the differing temperature sensitivities of the two competing deformation mechanisms.

We have included our response to this point in the paragraphs at the end of the review.

‘predicted to be intrinsically ductile at 0 K…’ … Nernst Theorem ?

We agree with the reviewer that this sentence is poorly written. We have changed it to:

“To understand the mechanical behavior of *bcc* alloys predicted to have a pronounced competition between dislocation slip and twinning emission from a crack tip at 0 K...[1]”

The paper we cite shows that atomic simulations of crack tips indicate there is a competition between slip and twinning as the mechanisms controlling crack-tip blunting. Our experimental results indicate that twinning is the global shielding mechanism, however. We have included a response to microplasticity at the end.

‘…it is also unclear how to reliably predict precise twinning stresses, but general trends indicate that alloying which promotes slip nearly always lowers twinning stresses relative to pure elements..’ The paper is sometimes imprecise and a bit handwaving on some of these interaction mechanisms: see e g the very broad overview of these observations which was compiled already in the 1973 paper by Mahajan and Williams (and several others…. ; see above), who synthesized most available experimental data across a range of BCC metals and alloys. They concluded that BCC metals exhibit a

negative temperature dependence of the critical twinning stress — meaning that twinning becomes easier as temperature falls — though the precise relationship is material-dependent and modulated by alloy composition, grain size, and strain rate. This paper was not considered here. Similarly, a comprehensive treatment of the subject is quite eminent review by Christian and Mahajan in 1995, Progress in Materials Science, a paper not considered by the authors... The BCC related part of this classic review synthesizes decades of experimental data and mechanistic modeling into a pretty coherent picture: below a critical temperature that shifts upward with increasing strain rate, the critical resolved shear stress for twinning becomes accessible before or alongside slip, and twinning emerges as a primary or co-operative deformation mode. This transition is not merely a low-temperature curiosity but has direct consequences for the ductile-to-brittle transition in BCC steels and alloys, since twins can act both as carriers of plasticity and as potent crack nucleation sites. This duality is also not really discussed in the current paper, although it had been addressed in vcery deep details before by Christian and others. In other words, some of the claims or argimention bits were already published (even with a more precise wording in part) more than 50 years ago – better make sure to consider these works on properly cite them when similar aspects are discussed.

We thank the reviewer for this insightful comment. We have removed the original sentence, as we agree that its phrasing could lead to ambiguity. Our intent was not to suggest a lack of understanding of general phenomenology, but rather to emphasize that the detailed alloying dependence of twinning stresses remains insufficiently resolved.

We also sincerely appreciate the reviewer for pointing out the foundational contributions of Mahajan and Williams and the comprehensive review by J. W. Christian and Mahajan. In response, we have cited some of the mentioned papers where relevant, particularly in the introduction. Please see the comments at the end where we address the mechanisms in these papers in detail.

“The expansive set of current and potential applications for bcc alloys, from building and transportation infrastructure to energy technologies, are often limited by this transition.” I understand that the authors want to underpin the importance, but again, extreme cryogenic applications are not so limiting as claimed here and in these fields usually FCC steels fo a good job, so better avoid any overstatements.

We agree that *fcc* alloys are the best cryogenic materials in terms of toughness, our group has done a lot of previous work demonstrating that this is the case with these systems. *Bcc* alloys do not have good properties at low temperatures compared to *fcc* alloys; however, we have found that this is not the case. For this reason, we think that this work is even more interesting and impactful because *bcc* alloys are avoided for cryogenic applications because of their DBTT. It is also not true that *bcc* alloys do not have

applications in these temperature regimes. Nb-Ti and Nb-Ta-Ti-Hf-Zr systems have exceptionally high superconductivity transition temperatures (and the high-entropy analogues can retain this transition at high pressures).[9,10] Moreover, these alloys can have good mechanical performance at high temperatures, whereas most single phase *fcc* alloys do not. This makes them attractive for many space applications (especially because some, like Nb-Ti, can achieve relatively low density). We have included additional citations to bolster this point.

I come back here to my last comments in the last review cycle : 'I do not quite understand this sentence: 'By inverting the sequence of deformation mechanisms typical in metals and alloys, tensile yielding and crack-tip blunting are controlled by temperature-insensitive defects that generate dislocation sources at interfaces where they intersect and elevate the local stress.' Inverting what ? dislos are active from the first moment, already in the microplastic regime in BCC. Dislos in BCC are for sure NOT temperature-insensitive mechanisms, but due to their high Peierls barriers they are actually much more temp-sensitive than FCC dislos. Maybe check this sentence – I could not really understand what the message is here. Maybe I misunderstood it?'
And I see that the authors stick to there wording of the 'inversion' and also use it now as a title – in view of the comments made above I still do not understand what they mean what exactly is inverted (compared to exactly what?). The many older papers cited above (and there are many more, properly done on high purity single crystals…) show clearly that these deformation mechanisms do not follow a linear sequence but they are highly nonlinearly linked. Some of that is simply not acknowledged by the authors here . I do not see the inversion argument here.

First, we agree that the word inversion can be confusing and so we have replaced it with the word competing, which we think accurately summarizes the interplay between twinning and slip. To clarify, we are not saying 'dislocations' are not needed for twinning. On the contrary, we are saying 'screw dislocation cores' transform from the regular compact core to a twinning core and twin propagates, from the transformed twin core before the original compact core becomes mobile (this was originally referred to in the discussion but is now also in the introduction). This restricts the yield stress to lower values (lower than what it would have been if the yield stress had been the stress to make the original compact core of the screws mobile (this we call dislocation slip stress)) and thereby give ductility to the alloy (We are saying yield stress and ductility have an inverse dependence). We are also saying that the screw dislocation twinning stress is *less* temperature dependent than the screw dislocation compact core mobility stress (It does not increase as steeply with decreasing temperature as the screw dislocation compact core mobility stress; we recognize the compact core mobility stress is highly temperature, strain rate, and orientation dependent). We have added clarifying statements throughout the abstract, introduction, results and discussion to clarify this point. As for the word

“inversion” itself, we called it inverted sequence as compared to what happens in *fcc* structures where you have dislocation slip first (analogous to compact core mobility stress in bcc structures) and then, twinning (which is also glide dislocation mediated). We understand that this is an entirely different crystal structure with different phenomena. However, we have used this word because (as the reviewer aptly pointed out) *fcc* alloys have no DBTT and are the toughest cryogenic materials we know of. Our previous work has shown that this is the case because of TWIP and TRIP.[2] As it is not mechanistically possible to “invert” this sequence in *fcc* materials, it therefore becomes quite unusual that in a *bcc* alloy the reversed sequence would also lead to cryogenic toughness, especially since this sequence has been historically associated with embrittlement.

Moreover, it is true that there is microplasticity before yielding, and that this microplasticity can occur by dislocation glide in grains and local regions where it is favored over twinning. We know that microplasticity also occurs by twinning as we observe some twinning-induced stress drops before yielding. However, when we say inverted, we are referring to the global mechanisms. Yielding (a global event) occurs by twinning (and the phase transformation). If it did not, the yield stress would appreciably increase because (and we absolutely agree with you) dislocations regardless of character have highly temperature dependent glide stresses. Global dislocation glide follows shortly thereafter and occurs by the glide of pre-existing dislocations with compact cores (that did not transform to twins), and dislocations nucleated by the twins. We also agree with the reviewer that this transition is not sudden; there is a range of temperatures across which it begins to occur. This is most likely the case in $Nb_{70}Ti_{30}$, as it is possible within our statistical variation that the yield stress increased (albeit not very rapidly). Finally, we know microplasticity by dislocation glide will happen in the vicinity of a crack tip as well; however, high toughness is generated by *crack-tip shielding which occurs over large length-scales*. This can only be achieved through a mechanism of global yielding, which twinning accomplished before dislocation glide. As a result, twinning can distribute damage away from the crack tip along much larger length-scales than dislocations *because* it is globally activated first.

To the reviewer’s point about originality. The slip-twinning transition is not a new phenomenon and we never claimed that it was (especially since our introduction is about it). However, it has always been associated with embrittlement, especially in the literature the reviewer has mentioned. We demonstrate that this is not necessarily true, and that in fact we attribute the lack of a DBTT in the tested alloys to the fact that this transition prevents a crack-tip plastic zone from undergoing a constriction. Moreover, tension and fracture toughness is rarely reported in the literature, often because these are more difficult properties to measure than compression.

Pasted below are some of the changes (with some of the newly cited papers the reviewer has referred us to as well as other papers we have found).

In the abstract:

“These materials instead exhibited a plateau in tensile yield strength and high fracture toughness at cryogenic temperatures which we attribute to the global activation of twinning and transformation induced plasticity before classical screw dislocation slip. Here, plastic deformation begins with twinning, mediated by screw dislocation core transformation, and a transformation to the omega phase, relatively temperature insensitive defects. Classical screw dislocation glide occurs at higher stresses.”

In the introduction:

“Beyond promoting dislocation glide, additional strategies for improving toughness involve activating multiple deformation mechanisms.[2] Twinning- and transformation-induced plasticity (TWIP and TRIP) can elevate toughness by increasing strain hardening and uniform ductility,[2–4] while some softening mechanisms, such as kink banding, can also elevate toughness by promoting plasticity.[5,6] Although TWIP and TRIP mechanisms are highly effective at increasing cryogenic damage tolerance in *fcc* alloys, their efficacy is limited in *bcc* alloys at low temperatures due to fundamental differences in the mechanisms controlling nucleation, propagation, and migration. This limitation arises from competition between temperature-dependent dislocation glide stresses and more temperature-insensitive twinning stresses which can converge at a slip-twinning transition.[7–9] Around this transition, it is hypothesized that the stress to transform ½<111> screw dislocation cores into three $^1/_6$<111> fractional dislocations that bind a propagating twin embryo is lower than for the same dislocation to retain its compact core and glide.[10,11] Phase transformations to omega (ω) can further decrease twin migration stresses and promote twin thickening.[12] Above this transition, twinning enhances ductility through the TWIP effect; however, near or below it, twins can instead promote brittle failure by impinging on interfaces.[13–18] Even though twinning prevents further increases in the yield stress (and therefore constriction of a crack-tip plastic zone),[13,19] the progressively rising barriers for dislocation glide impede the dissipation of damage at interfaces.[20] For this reason, this transition has predominantly been measured in compression, a stress-state that closes cracks.[13,19,20] Those that are measured in tension, such as in Fe-Si, show brittle behavior once the transition occurs.[21] Consequently, it has been largely seen as detrimental for mechanical properties.[22]”

In the results section:

“We believe twinning initiates due to screw dislocation core transformation from a regular compact core to a twinning core, and is accompanied by the omega transformation.[10,12]”

And...

“To address how this global ~~inverted~~ sequence of tensile deformation mechanisms inverted relative to *fcc* alloys, beginning with twinning coupled to a phase transformation and ending with screw dislocation glide, affects the fracture properties, we investigated compact-tension samples at the mid-thickness by Transmission Kikuchi Diffraction (TKD), and 4D-STEM.”

In the discussion section:

“As the applied stress increases, glide is progressively favored over twinning (~ 50% of the grains are in the anti-twinning orientation, they are expected to deform by screw dislocation glide with the original compact core).”

In the conclusion:

“Whereas most metallic alloys (particularly those with the *fcc* structure), regardless of crystal structure, generate high toughness from a mechanistic sequence that begins with dislocation glide and progresses to twinning and a phase transformation that delocalize dislocation induced damage,[2,5] this sequence in the current alloys begins with twinning (mediated by screw dislocation core transformation) and a phase transformation that nucleate dislocations that then glide to dissipate stress concentrations produced by the former two mechanisms.”

And in the Supplementary Information:

“While multiple experiments illustrate that the additional of this interstitial increases room temperature ductility by promoting strain hardening, it can simultaneously raise the DBTT to above 77 K.[23,24] It is also known to raise twinning and phase transformation stresses to additionally suppress these mechanisms.[9,25] However, this interplay is highly non-linear, *i.e.* O interstitials suppress the ω transformation more relative to twinning.[25] Further work to understand the role in suppressing ω on twin embryo growth and propagation is needed.”

“Moreover, experimental work on multiple alloys indicates that materials undergo the slip-twinning transition across a large temperature range, and that increases in grain size can promote twin propagation, but potentially suppress twin boundary migration.[20,21] In Fe-Si alloys, increasing grain size promotes embrittlement through this mechanism.[21] It is found that, like dislocations, twins obey a Hall-Petch relationship.[9] Further experimental work is needed to assess the Hall-Petch constants.”

1. Wang, W. *et al.* Ductility mechanisms in complex concentrated refractory alloys from atomistic fracture simulations. *npj Comput Mater* **11**, 330 (2025).

2. Liu, D. *et al.* Exceptional fracture toughness of CrCoNi-based medium- and high-entropy alloys at 20 kelvin. *Science* **378**, 978–983 (2022).

3. Gludovatz, B. *et al.* Exceptional damage-tolerance of a medium-entropy alloy CrCoNi at cryogenic temperatures. *Nature Communications* **7**, (2016).

4. Gludovatz, B. *et al.* A fracture-resistant high-entropy alloy for cryogenic applications. *Science* **345**, 1153–1158 (2014).

5. Cook, D. H. *et al.* Kink bands promote exceptional fracture resistance in a NbTaTiHf refractory medium-entropy alloy. *Science* **384**, 178–184 (2024).

6. Fan, X. J., Qu, R. T. & Zhang, Z. F. Remarkably high fracture toughness of HfNbTaTiZr refractory high-entropy alloy. *Journal of Materials Science & Technology* **123**, 70–77 (2022).

7. Meyers, M. A., Vöhringer, O. & Lubarda, V. A. The onset of twinning in metals: a constitutive description. *Acta Materialia* **49**, 4025–4039 (2001).

8. Mahajan, S. & Williams, D. F. Deformation Twinning in Metals and Alloys. *International Metallurgical Reviews* **18**, 43–61 (1973).

9. Christian, J. W. & Mahajan, S. Deformation twinning. *Progress in Materials Science* **39**, 1–157 (1995).

10. Sleeswyk, A. W. ½<111> screw dislocations and the nucleation of 112<111> twins in the b.c.c. lattice. *The Philosophical Magazine: A Journal of Theoretical Experimental and Applied Physics* **8**, 1467–1486 (1963).

11. Xiao, J. *et al.* Unveiling deformation twin nucleation and growth mechanisms in BCC transition metals and alloys. *Materials Today* **65**, 90–99 (2023).

12. Chen, G., Li, D., Zheng, Y. & Qi, L. Stability and growth kinetics of {112} twin embryos in β-Ti alloys. *Acta Materialia* **263**, 119520 (2024).

13. Marcinkowski, M. J. & Lipsitt, H. A. The plastic deformation of chromium at low temperatures. *Acta Metallurgica* **10**, 95–111 (1962).

14. Edmondson, B. & Allen, N. P. The effect of composition and structure on the tensile properties of high-purity iron single crystals, with particular reference to the question of brittle fracture. *Proceedings of the Royal Society of London. Series A. Mathematical and Physical Sciences* **264**, 176–197 (1997).

15. Reid, C. N. A review of mechanical twinning in body-centred cubic metals and its relation to brittle fracture. *Journal of the Less Common Metals* **9**, 105–122 (1965).

16. Reid, C. N., Gilbert, A. & Hahn, G. T. *Investigation of the Relationship of Twinning to Brittle Fracture of Refractory Metals*. (Directorate of Materials and Processes, Aeronautical Systems Division, Air Force Systems Command, United States Air Force, 1963).

17. Liu, L., Wu, H. C., Wang, J., Gong, S. K. & Mao, S. X. Twinning-dominated nucleation, propagation and deflection of crack in molybdenum characterized with in situ transmission electron microscopy: Philosophical Magazine Letters: Vol 94, No 4. *Philosophical Magazine Letters* **94**, 225–232 (2014).

18. Zhang, Y., Millett, P. C., Tonks, M. & Biner, B. Deformation-twin-induced grain boundary failure. *Scripta Materialia* **66**, 117–120 (2012).

19. Senkov, O. N., Rao, S. I., Balbus, G. H., Wheeler, R. & Payton, E. J. Microstructure and deformation behavior of MoReW in the temperature range from 25°C to 1500°C. *Materialia* **27**, 101688 (2023).

20. Hinrichs, F. *et al.* A ductile chromium–molybdenum alloy resistant to high-temperature oxidation. *Nature* **646**, 331–337 (2025).

21. Hull, D. Effect of grain size and temperature on slip, twinning and fracture in 3% silicon iron. *Acta Metallurgica* **9**, 191–204 (1961).

22. Reid, C. N. The association of twinning and fracture in bcc metals. *Metall Trans A* **12**, 371–377 (1981).

23. Wang, S. *et al.* The effects of interstitial oxygen on mechanical properties of TiZrNb medium-entropy alloy over a wide temperature range. *Journal of Alloys and Compounds* **989**, 174394 (2024).

24. Lei, Z. *et al.* Enhanced strength and ductility in a high-entropy alloy via ordered oxygen complexes. *Nature* **563**, 546–550 (2018).

25. Borges, P. P. P. O., Ritchie, R. O. & Asta, M. Effects of interstitial oxygen on ω transformations and twin formation in bcc NbTaTiHf multi-principal element alloy from first-principles. *Computational Materials Science* **266**, 114569 (2026).

Competing mechanisms ductilize *bcc* binary and medium-entropy alloys at 20 K

David H. Cook[1,2#], Xiaoyu Chen[1,2#], Michael O'Leary[3], Satish Rao[2], Wenqing Wang[1,2], Calvin H. Belcher[4], Madelyn Payne[1,2], Pedro Borges[1,2], Tung Lik Lee[5], Arun Devaraj[6], Mark Asta[1,2], Enrique J. Lavernia[7], Diran Apelian[4], Andrew M. Minor[1,8], Dong Liu[3,9*], Punit Kumar[1,10*], and Robert O. Ritchie[1,2*]

[1]Department of Materials Science and Engineering, University of California, Berkeley, CA, USA
[2]Materials Sciences Division, Lawrence Berkeley National Laboratory, Berkeley, CA, USA
[3]Department of Physics, University of Bristol, UK
[4]Department of Materials Science and Engineering, University of California, Irvine, CA, USA
[5]ISIS Neutron and Muon Source, Rutherford Appleton Laboratory, Didcot, UK
[6]Physical and Computational Sciences Directorate, Pacific Northwest National Laboratory, Richland, WA, USA
[7]Department of Materials Science and Engineering, Texas A&M University, TX, USA
[8]National Center for Electron Microscopy, Molecular Foundry, Lawrence Berkeley National Laboratory, Berkeley, CA, USA
[9]Department of Engineering Science, University of Oxford, UK
[10]School of Mechanical and Aerospace Engineering, Nanyang Technological University, Singapore

[#]Authors with equal contribution, [*]Corresponding authors

Abstract: Plastic deformation in body-centered cubic (*bcc*) metals and alloys is conventionally mediated by screw dislocations. Their temperature-sensitive motion is attributed to a steep rise in strength with decreasing temperature that constricts plastic zones at crack tips, leading to a ductile-to-brittle transition (DBTT).[1] An evaluation of the mechanical properties of group IV-V *bcc* refractory alloys with similar electronic structures at 20 K revealed that these materials instead exhibited a plateau in tensile yield strength and fracture toughness as a function of temperature, with no apparent DBTT. This behavior arises from the global activation of twinning and an omega phase transformation, which nucleate dislocations at interfaces prior to global dislocation motion. The initial mechanism is temperature-insensitive and controls yielding, thereby preventing a crack-tip plastic zone from constricting with decreasing temperature.

INTRODUCTION

Alloys with the body-centered cubic (*bcc*) crystal structure have a ductile-to-brittle transition temperature (DBTT) below which plastic deformation is restricted, leading to embrittlement.[1,2] This problem has plagued *bcc* materials ranging from steels at or slightly below room temperature to refractory alloys up to 1200°C.[3,4] The expansive set of current and potential applications for *bcc* alloys, such as refractory multi-principal alloys (commonly known as medium- or high-entropy alloys), for building and transportation infrastructure, energy technologies, and hypersonic applications, is often limited by this transition.[5,6]

This limitation arises from their non-closed-packed crystal structure, which forces dislocations, specifically screws, to rely on thermally activated kink-pair nucleation to move.[2,7] Consequently, screw dislocation glide becomes sluggish at low temperatures, causing strength to increase precipitously and plasticity to be constricted at crack tips until fully brittle failure ensues.[8] Traditional strategies to suppress the DBTT primarily rely on facilitating screw dislocation glide through careful substitutional and interstitial alloying[9–12]: while interstitial elements raise DBTTs by introducing additional pinning stresses, substitutional alloying can, in some cases, ductilize *bcc* materials.[11] This has been observed in refractory binary and multi-principal element alloys (RMPEAs), which are often extremely brittle.[4,11,13] Here, concentrated additions of group IV (Ti, Zr, Hf) into group V (V, Nb, Ta) elements enhance screw dislocation glide by lowering the unstable stacking-fault energies and raising the kink-pair equilibrium concentration.[11,14]

Beyond promoting dislocation glide, additional strategies for improving toughness involve activating multiple deformation mechanisms.[15] Twinning- and transformation-induced plasticity (TWIP and TRIP) can elevate toughness by increasing strain hardening and uniform ductility,[15,16] while some softening mechanisms, such as kink banding, can also elevate toughness by promoting plasticity.[17,18] Although TWIP and TRIP mechanisms are highly effective at increasing cryogenic damage tolerance in *fcc* alloys, their efficacy is limited in *bcc* alloys at low temperatures due to fundamental differences in the mechanisms controlling nucleation, propagation, and migration. This limitation arises from a competition between temperature-dependent dislocation glide stresses and more temperature-insensitive twinning stresses, which can converge at a slip-twinning transition.[19–21] Around this transition, it is hypothesized that the stress to transform ½<111> screw dislocation cores into three $^{1}/_{6}$<111> fractional dislocations that bind a propagating twin embryo is lower than for the same dislocation to retain its compact core and glide.[22,23] This process can be accompanied by a phase transformation to omega (ω), which can further decrease twin migration stresses and promote twin thickening.[24] Above this transition, twinning enhances ductility through the TWIP effect; however, near or below it, twins instead promote brittle failure by impinging on interfaces.[25–27] Even though twinning prevents further increases in yield stress (and therefore the constriction of the crack-tip plastic zone),[25,27] the inherently high barrier to dislocation nucleation in alloys impedes the dissipation of damage at interfaces. For this reason, this transition causes brittle failure in tension (*e.g.,* in Fe-Si) and therefore is nearly always measured in compression, a stress state that closes cracks.[25,28]

Nevertheless, there is evidence that the slip-twinning transition can occur in alloys that do not exhibit a clear DBTT, such as $Nb_{45}Ta_{25}Ti_{15}Hf_{15}$ and an electronically similar, but chemically simpler, $Nb_{70}Ti_{30}$ alloy.[17,29] Previous work has demonstrated that $Nb_{45}Ta_{25}Ti_{15}Hf_{15}$ possesses exceptional strength and toughness across a wide temperature range due to a combination of dislocation glide, twinning, and kink-banding.[17] Together, these mechanisms sustain plasticity and suppress brittle failure, resulting in a fracture toughness of up to 90 MPa√m at 77 K. This combination of factors leaves the role of this transition in fracture behavior in alloys that can retain

tensile ductility at this mechanistic transition unclear. To understand the mechanical behavior of these *bcc* alloys, we investigated their properties at even lower temperatures of ~20 K by performing uniaxial tensile tests and non-linear elastic *J*-based fracture toughness tests. In addition to measuring bulk mechanical properties, we performed extensive post-fracture electron backscatter diffraction (EBSD), fractography, and transmission electron microscopy (TEM) to investigate the plastic deformation mechanisms and their spatio-temporal evolution with increasing strain and stress. We found that these *bcc* alloys nucleate dislocations instead of cracks at twin- and omega-boundary intersection sites, preventing premature brittle fracture and enabling the plastic zone to form via temperature-insensitive mechanisms.

RESULTS

As described in more detail in the *Supplementary Materials*, the $Nb_{45}Ta_{25}Ti_{15}Hf_{15}$ and $Nb_{70}Ti_{30}$ alloys were arc-melted, forged and cross-rolled to a 33% reduction in thickness, and heat treated at ~56% of their solidus temperature for one hour to produce a single-phase, near-fully recrystallized, equiaxed grain structure with an average grain size of ~259 µm for $Nb_{45}Ta_{25}Ti_{15}Hf_{15}$ and a heterogeneous, bimodal distribution ~51–412 µm for $Nb_{70}Ti_{30}$ (Extended Fig. 1). Both alloys contain low interstitial concentrations: $Nb_{45}Ta_{25}Ti_{15}Hf_{15}$ has 0.059 at. % (60 ppmw) C, 0.0495 at.% (58 ppmw) N, and 0.146 at. % (197 ppmw) O; $Nb_{70}Ti_{30}$ has 0.278 at. % (561 ppmw) O and 0.0625 at. % (111 ppmw) N.[17]

Representative tensile stress-strain curves and crack-resistance curves (*R*-curves) measured in terms of the *J*-integral (kJ/mm^2) as a function of crack extension, Δa (mm), are shown in Figs. 1(a) and (b) for $Nb_{45}Ta_{25}Ti_{15}Hf_{15}$ and Extended Fig. 2 for $Nb_{70}Ti_{30}$. The corresponding tensile stress-strain curves and *R*-curves at 77 and 298 K are also shown for comparison.[17] In $Nb_{45}Ta_{25}Ti_{15}Hf_{15}$, the yield strength at 20 K ($\sigma_y \approx 938.9 \pm 22.6$ MPa) is comparable to that measured at 77 K ($\sigma_y \approx 936.6 \pm 22.4$ MPa) (Fig. 1(d)). The ultimate tensile stress ($\sigma_{UTS} \approx 1149.5 \pm 3.4$ MPa) is ~35 MPa higher than that measured at 77 K, while the uniform ductility and the elongation to fracture decrease by ~33% and ~50%, respectively.[17] A similar effect occurs in $Nb_{70}Ti_{30}$: the yield strength at 77 K ($\sigma_y \approx 825.8 \pm 49.4$ MPa) is only 14 MPa lower than at 20 K ($\sigma_y \approx 839.9 \pm 18.5$ MPa), within the statistical variation. In both alloys, yielding at cryogenic temperatures is accompanied by stress drops and acoustic emission consistent with twinning. An evaluation of the instantaneous strain hardening in $Nb_{45}Ta_{25}Ti_{15}Hf_{15}$ (Fig. 1(c)) illustrates that at 298 K the hardening remains high at all strains, but with decreasing temperature, it is continuously less sustained with increasing strain. In the presence of a crack, the material exhibits rising R-curve behavior, *i.e.,* its resistance to fracture increases with increasing crack extension (Fig. 1(b)). The size-independent crack-initiation toughness, K_{JIc}, and crack-growth toughness, K_{ss} (defined as the toughness at maximum allowable crack extension), are calculated from the *R*-curves (*see Materials and Methods section*).[30] In $Nb_{45}Ta_{25}Ti_{15}Hf_{15}$, the crack-initiation toughness, K_{JIc}, is ~96.5 MPa√m, and the steady-state crack-growth toughness, K_{ss}, is ~118 MPa√m (Fig. 1(e)) at 20 K. Similarly, $Nb_{70}Ti_{30}$ has a crack-initiation toughness of 170 MPa√m at 20 K, showing little change from 188 and 219 MPa√m at 77 K and 298 K, respectively. These properties are summarized in Extended Table 1.

Despite the high toughness and ductility at 20 K, the fracture surfaces in both alloys show differences between tensile and compact-tension C(T) specimens used for the fracture tests (Fig. 2 (a)–(b), Supplementary Fig. 1-2, Extended Fig. 3). Here, it is important to note that in the vicinity of a crack, there are three principal stresses that are each approximately ~3–5 times higher than the one principal stress in tensile specimens. The triaxial stress-state generates a hydrostatic stress,

suppresses shear stresses, and is additionally graded.[31] Whereas in uniaxial tension, fracture is fully ductile and occurs by microvoid coalescence, under crack-tip stress triaxiality, there are some signs of brittle features. Specifically, fracture is partly intergranular/partly ductile, with crystallographically oriented bands and microvoids along grain boundaries, a transition that departs from fully ductile fracture at higher temperatures. The differences generated between the two stress states are further illustrated by EBSD band contrast (BC) and inverse pole figure (IPF) maps of tensile samples and at the mid-thickness of compact-tension samples, where plane-strain conditions and hydrostatic stresses are highest (Fig. 2 (c)–(d)). While both samples exhibit profuse {112}<111> nano-twinning, tensile samples have higher levels of dislocation glide than compact-tension samples. In the latter, there are some kink bands (significantly fewer than at higher temperatures), indicating edge dislocation glide.

To investigate the microscopic deformation mechanisms underlying this atypical mechanical behavior, we first analyzed the sequence of tensile deformation mechanisms. Tensile specimens of $Nb_{45}Ta_{25}Ti_{15}Hf_{15}$—one interrupted shortly after yielding $(\varepsilon_p^T = 0.004, \sigma^T \approx 1013\ MPa)$ and a second at a higher uniform strain $(\varepsilon_p^T = 0.049, \sigma^T \approx 1219\ MPa)$—were jet polished and examined in a TEM. These experiments reveal that twinning coupled with a phase transformation to omega initiates yielding. Virtual dark-field reconstructions of four-dimensional scanning transmission electron microscopy (4D-STEM) scans correlated with high-resolution TEM (HRTEM) images of tensile specimens interrupted at 0.4% plastic strain (Figs. 3(a)–(c), Extended Fig. 4(a)-(d)), show conventional {112}<111> nanotwins, with a nanometer-thick interfacial layer of ω that expands in the space between the twins. Indexed diffraction patterns are shown in Supplementary Fig. 3. We also observe twinning controlling yielding at 77 K (Extended Fig. 4(g)-(h)). We did not observe any other twinning systems. Although it is unclear whether the crystal collapsed to ω first to aid twin nucleation second, or if the local shear stress from the twin embryo growth and propagation triggered the ω transformation (Fig. 3(b)-(c)), in either case, this phase can assist twin nucleation and growth.[24,32–34] We also tracked the twins to their termination sites at interfaces. Here, twins produce stress concentrations high enough to nucleate dislocations (Fig. 3(d)). As deformation progresses to the uniform strain, these dislocations (invariably combined with pre-existing ones in the undeformed material) select more sluggish screw orientations to mediate plastic flow at higher strains (Fig. 3(e)–(f), Supplementary Fig. 4). This sequence is summarized in EBSD maps in Extended Fig. 5.

To address how this global sequence of tensile deformation mechanisms (we us the word global to exclude cases of microplasticity in which local regions may favor dislocation glide prior to twinning)~~ inverted relative to *fcc* alloys, beginning with twinning coupled to a phase transformation and ending with screw dislocation glide~~ affects the fracture properties, we investigated compact-tension samples at the mid-thickness (where the stress triaxiality would be highest due to plane-strain conditions) by Transmission Kikuchi Diffraction (TKD) and 4D-STEM. While EBSD maps in Fig. 2 provide an overview of the crack-tip deformation mechanisms, it remains unclear why the fracture is nominally intergranular. To identify the cause, we performed TKD and 4D-STEM on regions near grain boundaries to capture the deformation mechanisms occurring in the regions of highest stress prior to crack extension. FIB lift-outs were taken from a grain boundary several micrometers ahead of a crack tip and from an intergranular fracture facet with the distinctive crystallographic deformation markings in Fig. 2(a). Precise calculation of stress and strain using the Hutchinson–Rice–Rosengren (HRR) crack-tip plastic strain field is detailed in the *Materials and Methods* section. Reconstructed virtual dark-field images of 4D-STEM maps in Fig. 4(a)-(c) with indexed diffraction patterns in Supplementary Fig. 5 show similar

deformation structures to those seen at yielding in tensile samples: fine nanotwins accompanied by omega impinging on a grain boundary. Additional instances of twins forming the crystallographic microvoids in Fig. 1(e) on fractured grain boundaries are documented in Extended Fig. 6. However, any intergranular fracture that it could cause is delayed by dislocations nucleated in the parent and neighboring grain, some of which were identified as edge and mixed dislocations by a $g \cdot b$ analysis (Figs. 4(d)-(e), Supplementary Fig. 6). The shear stresses are high enough for edge dislocations to glide and form micro-scale kink bands, as we observed with TKD in regions close to the fracture surface (Fig. 4(f), Extended Fig. 6).[35] Other dislocations form channels along which deformation is localized (Fig. 4(a)).

DISCUSSION

Our results indicate that nonlinear interactions among four deformation mechanisms—twinning, phase transformations, dislocation glide, and dislocation nucleation—can lead to competitive behavior amongst them as a function of temperature. This manifests below 77 K as a global sequence of tensile deformation mechanisms that nominally begins with twinning/transformation and ends with dislocation glide. However, a pervasive question remains: why does the slip-twinning transition inhibit the DBTT in these NbTi alloys but cause low-temperature embrittlement in all other *bcc* materials observed to date?[27]

To discern this variable, we used experimental and computationally measured values to quantify each mechanism. First, we obtained twinning and dislocation glide stresses for both alloys. Twinning stresses were obtained from tensile curves, whereas screw (with the original compact core) and edge dislocation tensile glide stresses were calculated using, respectively, the Rao-Suzuki model and the Maresca-Curtin model at 20 K with the experimental strain rate of $1 \times 10^{-3}\ s^{-1}$.[36–38] These values are listed in Table 1. While the tensile glide stresses are only ~30% higher in $Nb_{45}Ta_{25}Ti_{15}Hf_{15}$ compared to $Nb_{70}Ti_{30}$, these apparently small differences in uniaxial tension can be exacerbated at a crack tip by the high hydrostatic stresses.[3] This is easily visualized by mapping these stresses onto the HRR fields in Fig. 4(g)-(h) and Extended Fig. 7. As can be seen in Fig. 4(g), the local shear stresses at the crack tip are high enough to activate twinning in $Nb_{45}Ta_{25}Ti_{15}Hf_{15}$ and $Nb_{70}Ti_{30}$, but they are not high enough in $Nb_{45}Ta_{25}Ti_{15}Hf_{15}$ to move pre-existing dislocations, except for within a small region close to the crack tip. Lower glide barriers in $Nb_{70}Ti_{30}$ generate a strain-controlled fracture, as evidenced by the very high and homogeneous dislocation distributions (despite local damage produced at twin-omega-interface sites) (Extended Fig. 8) and increased ductile fracture surface features in C(T) samples (Extended Fig. 3).

These observations illustrate that lowering glide stresses enables global activation of dislocation glide at a crack tip, but it is not necessarily the critical mechanism preventing brittle fracture because the toughness of $Nb_{45}Ta_{25}Ti_{15}Hf_{15}$ is still nearly 100 MPa√m. These experiments indicate that chemical complexity raises the glide stresses, which elevates high-temperature strength, albeit with a modest cost to toughness, but that it is not a variable involved in determining the slip-twinning transition or DBTT.[17] We deduce that it is the combination of low twinning migration stresses and, *most critically*, low barriers to heterogeneous dislocation nucleation which is primarily a function of the electronic structure. Our observation in two tensile stress states is that twin-grain boundary sites nucleate dislocations instead of cracks, which (i) enables the generation of a plastic zone by twinning, and (ii) suggests dislocation nucleation (not glide) is a primary driver of the DBTT. While dislocation nucleation stresses are generally very high and often difficult to compute, Density Functional Theory (DFT) studies show that adding group IV elements reduces the unstable stacking-fault energy of group V metals and alloys, a proxy for

dislocation nucleation barriers.[11] These elements concomitantly promote twinning and the omega phase transformation.[23,39] However, by promoting the transformation to ω, a phase predicted by the Rice parameter to be brittle, the group IV elements become a "double-edged sword."[40] This transformation is especially problematic for samples containing cracks because high hydrostatic stress promotes stress-controlled transformations and exploits the new phase's brittleness to accelerate fracture.[41,42] DFT calculations at 0 K reveal that in NbTaTiHf alloys, increasing group IV concentrations promote lattice instabilities from *bcc* to ω.[39] At 30 at.% Ti and Hf, the *bcc* phase in $Nb_{45}Ta_{25}Ti_{15}Hf_{15}$ is still relatively stable.[39] But the composition is heterogeneous. Atom probe tomography (APT) analysis of grain boundaries in Extended Fig. 9(a)-(b) shows that Ti and Hf enrich the boundaries in a ~3 nm range, raising the group IV concentration to 42 at.% in the measured region. A TEM energy-dispersive X-ray (EDX) analysis (Extended Fig. 9(c)-(h)) of the grain boundary from Fig. 4 at the twin/omega impact site indicates this boundary is also enriched in Ti and Hf. This produces a local environment that promotes dislocation nucleation, but it also produces an additional chemical driving force for stress-induced ω formation.[43]

CONCLUSION

To summarize, our findings demonstrate that NbTi-based alloys can retain exceptional damage tolerance, with no measurable ductile-to-brittle transition observed down to 20 K. These properties are derived from a global mechanistic sequence that is unusual for *bcc* alloys in tension, which are often susceptible to high-temperature DBTTs.[4] Whereas most metallic alloys (particularly those with the *fcc* structure) generate high toughness from a global sequence that begins with dislocation glide and progresses to twinning and a phase transformation that delocalizes dislocation-induced damage;[15,17] the global sequence in the current alloys begins with twinning (mediated by screw dislocation core transformation) coupled to a phase transformation that nucleate dislocations to dissipate stress concentrations produced by the former two mechanisms. Dislocation glide subsequently follows. This achieves an unconventional pathway to inhibit a DBTT: crack-tip blunting is initiated by twinning, a near-temperature-insensitive mechanism that prevents a crack-tip plastic zone from shrinking and serves as an efficient dislocation source. Our results illustrate another pathway, independent of chemical complexity, for inhibiting the DBTT in refractory *bcc* alloys.

Stress-state	Alloy	Screw Dislocations	Edge dislocations	{112}<111> Twinning	Omega Transformation	Kink Bands
**Uniaxial Tension** (Activation stress)	$Nb_{45}Ta_{25}Ti_{15}Hf_{15}$	Yes (2) 1048–1384 MPa (20–10b)	No 740 MPa	Yes (1) 775–938 MPa	Yes (1)	No
**Crack-Tip Stress Triaxiality**	$Nb_{45}Ta_{25}Ti_{15}Hf_{15}$	No	Yes (2)	Yes (1)	Yes (1)	Yes (3)
**Uniaxial Tension** (Activation stress)	$Nb_{70}Ti_{30}$	Yes (2) 1080 MPa (10b)	— 105 MPa	Yes (1) 473–840 MPa	—	—
**Crack-Tip Stress Triaxiality**	$Nb_{70}Ti_{30}$	— (2)	—	Yes (1)	Yes (1)	—

Table 1. A Summary of mechanisms observed in each stress state. Their sequential order with increasing stress is indicated in parentheses. Mechanisms that are not directly observed are indicated with em dashes.

REFERENCES

1. Takeuchi, S., Hashimoto, T. & Maeda, K. Plastic Deformation of bcc Metal Single Crystals at Very Low Temperatures. *Trans. JIM* **23**, 60–69 (1982).
2. Christian, J. W. Some surprising features of the plastic deformation of body-centered cubic metals and alloys. *Metall Trans A* **14**, 1237–1256 (1983).
3. Ritchie, R. O., Knott, J. F. & Rice, J. R. On the relationship between critical tensile stress and fracture toughness in mild steel. *Journal of the Mechanics and Physics of Solids* **21**, 395–410 (1973).
4. Kumar, P. *et al.* Degradation of the mechanical properties of NbMoTaW refractory high-entropy alloy in tension. *Acta Materialia* **279**, 120297 (2024).
5. Guo, J. *et al.* Robust zero resistance in a superconducting high-entropy alloy at pressures up to 190 GPa. *Proceedings of the National Academy of Sciences* **114**, 13144–13147 (2017).
6. Senkov, O., Miracle, D., Chaput, K. & Couzinie, J.-P. Development and exploration of refractory high entropy alloys—A review. *Journal of Materials Research* **33**, 3092–3128 (2018).
7. Dezerald, L., Proville, L., Ventelon, L., Willaime, F. & Rodney, D. First-principles prediction of kink-pair activation enthalpy on screw dislocations in bcc transition metals: V, Nb, Ta, Mo, W, and Fe. *Phys. Rev. B* **91**, 094105 (2015).
8. Ashby, M., Shercliff, H. & Cebon, D. Fracture and fracture toughness. in *Materials* 203–230 doi:10.1016/B978-0-08-102376-1.00008-1.
9. Wang, S. *et al.* The effects of interstitial oxygen on mechanical properties of TiZrNb medium-entropy alloy over a wide temperature range. *Journal of Alloys and Compounds* **989**, 174394 (2024).
10. Belcher, C. H., MacDonald, B. E., Apelian, D. & Lavernia, E. J. The role of interstitial constituents in refractory complex concentrated alloys. *Progress in Materials Science* **137**, 101140 (2023).
11. Borges, P. P. P. O., Ritchie, R. O. & Asta, M. Electronic descriptors for dislocation deformation behavior and intrinsic ductility in bcc high-entropy alloys. *Science Advances* **10**, eadp7670 (2024).
12. Borges, P. P. P. O., Ritchie, R. O. & Asta, M. Chemical trends favoring interstitial cluster

formation in bcc high-entropy alloys from first-principles calculations. *Acta Materialia* **294**, 121091 (2025).
13. Huang, H. *et al.* Achieving high tensile strength and ductility in refractory alloys by tuning electronic structure. *Nat. Mater.* 1–9 (2026) doi:10.1038/s41563-025-02464-8.
14. Tsuru, T. *et al.* Intrinsic factors responsible for brittle versus ductile nature of refractory high-entropy alloys. *Nat Commun* **15**, 1706 (2024).
15. Liu, D. *et al.* Exceptional fracture toughness of CrCoNi-based medium- and high-entropy alloys at 20 kelvin. *Science* **378**, 978–983 (2022).
16. Gludovatz, B. *et al.* Exceptional damage-tolerance of a medium-entropy alloy CrCoNi at cryogenic temperatures. *Nature Communications* **7**, (2016).
17. Cook, D. H. *et al.* Kink bands promote exceptional fracture resistance in a NbTaTiHf refractory medium-entropy alloy. *Science* **384**, 178–184 (2024).
18. Fan, X. J., Qu, R. T. & Zhang, Z. F. Remarkably high fracture toughness of HfNbTaTiZr refractory high-entropy alloy. *Journal of Materials Science & Technology* **123**, 70–77 (2022).
19. Meyers, M. A., Vöhringer, O. & Lubarda, V. A. The onset of twinning in metals: a constitutive description. *Acta Materialia* **49**, 4025–4039 (2001).
20. Mahajan, S. & Williams, D. F. Deformation Twinning in Metals and Alloys. *International Metallurgical Reviews* **18**, 43–61 (1973).
21. Christian, J. W. & Mahajan, S. Deformation twinning. *Progress in Materials Science* **39**, 1–157 (1995).
22. Sleeswyk, A. W. ½<111> screw dislocations and the nucleation of 112<111> twins in the b.c.c. lattice. *The Philosophical Magazine: A Journal of Theoretical Experimental and Applied Physics* **8**, 1467–1486 (1963).
23. Xiao, J. *et al.* Unveiling deformation twin nucleation and growth mechanisms in BCC transition metals and alloys. *Materials Today* **65**, 90–99 (2023).
24. Chen, G., Li, D., Zheng, Y. & Qi, L. Stability and growth kinetics of {112} twin embryos in β-Ti alloys. *Acta Materialia* **263**, 119520 (2024).
25. Marcinkowski, M. J. & Lipsitt, H. A. The plastic deformation of chromium at low temperatures. *Acta Metallurgica* **10**, 95–111 (1962).
26. Edmondson, B. & Allen, N. P. The effect of composition and structure on the tensile properties of high-purity iron single crystals, with particular reference to the question of brittle fracture. *Proceedings of the Royal Society of London. Series A. Mathematical and Physical Sciences* **264**, 176–197 (1997).
27. Reid, C. N. A review of mechanical twinning in body-centred cubic metals and its relation to brittle fracture. *Journal of the Less Common Metals* **9**, 105–122 (1965).
28. Hull, D. Effect of grain size and temperature on slip, twinning and fracture in 3% silicon iron. *Acta Metallurgica* **9**, 191–204 (1961).
29. Begley, R. T. & Bechtold, J. H. Effect of alloying on the mechanical properties of niobium. *Journal of the Less Common Metals* **3**, 1–12 (1961).
30. ASTM E1820: Standard Test Method for Measurement of Fracture Toughness, 2023. (2023).
31. Ritchie, R. O. & Thompson, A. W. On macroscopic and microscopic analyses for crack initiation and crack growth toughness in ductile alloys. *Metall Trans A* **16**, 233–248 (1985).
32. Wu, S. Q. *et al.* {112}<111> Twinning during ω to body-centered cubic transition. *Acta Materialia* **62**, 122–128 (2014).
33. Chen, B. & Sun, W. Omega transitional structure associated with {112}<111> deformation twinning in a metastable beta Ti-Nb alloy, revealed by atomic resolution high-angle annular

dark-field scanning transmission electron microscopy. *Journal of Alloys and Compounds* **766**, 123–130 (2018).
34. Xing, H. & Sun, J. Mechanical twinning and omega transition by □111□ {112} shear in a metastable β titanium alloy. *Applied Physics Letters* **93**, 031908 (2008).
35. Yamasaki, M., Hagihara, K., Inoue, S., Hadorn, J. P. & Kawamura, Y. Crystallographic classification of kink bands in an extruded Mg–Zn–Y alloy using intragranular misorientation axis analysis. *Acta Materialia* **61**, 2065–2076 (2013).
36. Lee, C. *et al.* Strength can be controlled by edge dislocations in refractory high-entropy alloys. *Nat Commun* **12**, 5474 (2021).
37. Rao, S. I., Woodward, C., Akdim, B., Senkov, O. N. & Miracle, D. Theory of solid solution strengthening of BCC Chemically Complex Alloys. *Acta Materialia* **209**, 116758 (2021).
38. Wang, W. *et al.* A theoretical study of solid solution strengthening in the refractory medium entropy alloy Nb45Ta25Ti15Hf15. *Materials & Design* **261**, 115368 (2026).
39. Borges, P. P. P. O., Ritchie, R. O. & Asta, M. Local lattice distortions and the structural instabilities in bcc Nb–Ta–Ti–Hf high-entropy alloys: An ab initio computational study. *Acta Materialia* **262**, 119415 (2024).
40. Borges, P. P. P. O., Ritchie, R. O. & Asta, M. Effects of interstitial oxygen on ω transformations and twin formation in bcc NbTaTiHf multi-principal element alloy from first-principles. *Computational Materials Science* **266**, 114569 (2026).
41. Banerjee, D. & Williams, J. C. Perspectives on Titanium Science and Technology. *Acta Materialia* **61**, 844–879 (2013).
42. Ballor, J., Li, T., Prima, F., Boehlert, C. J. & Devaraj, A. A review of the metastable omega phase in beta titanium alloys: the phase transformation mechanisms and its effect on mechanical properties. *International Materials Reviews* **68**, 26–45 (2023).
43. Devaraj, A. *et al.* Experimental evidence of concurrent compositional and structural instabilities leading to ω precipitation in titanium–molybdenum alloys. *Acta Materialia* **60**, 596–609 (2012).

MAIN FIGURE LEGENDS

Figure 1. Mechanical properties of NbTi-based alloys. (a) Tensile engineering stress-strain curves of $Nb_{45}Ta_{25}Ti_{15}Hf_{15}$ with yield strength plotted as a function of temperature in **(d)** and instantaneous strain hardening curves extracted in **(c). (b)** *J*-R curves show the resistance to fracture of the $Nb_{45}Ta_{25}Ti_{15}Hf_{15}$ alloy as a function of crack extension. Corresponding *K*-based fracture toughness values (crack-initiation toughness, $K_{J\mathrm{Ic}}$, and steady-state crack-growth toughness, K_{ss}) back-calculated from **(b)** are shown in **(d)** as a function of temperature. Both values met the criteria for *J*-dominance and plane-strain conditions. Note: the slightly lower crack-initiation toughness ($K_{J\mathrm{Ic}}$) at 77 K compared to 20 K in $Nb_{45}Ta_{25}Ti_{15}Hf_{15}$ is attributed to the statistical variation related to processing defects and microstructure in the tested samples.

Figure 2. Fracture morphology and EBSD maps of $Nb_{45}Ta_{25}Ti_{15}Hf_{15}$. The effect of stress-state is illustrated by differences in fracture morphology observed between C(T) and uniaxial tensile samples. Whereas in C(T) specimens in **(a)** fracture occurs by crystallographic microvoids on grain boundaries, in tensile specimens in **(b)** it occurs by microvoid coalescence. This is further exemplified by EBSD IPF and BC maps that show profuse {112}<111> twinning in **(c)** compact-tension and **(d)** tensile specimens but significantly more deformation by dislocation glide in tension in **(d)**.

Figure 3. Sequence of uniaxial tensile deformation mechanisms. (a)–(c) virtual dark-field images reconstructed from 4D-STEM scans of two twins with ω collapse occurring between them. Omega is additionally observed in **(b)** as a transitional twin boundary structure. Once the twins propagate, they impact interfaces such as grain boundaries, as shown in **(d)**, generating a local stress concentration that nucleates mixed dislocations. At higher plastic strains, the dislocations can glide into the grain interiors as shown in **(e).** A $g \cdot b$ analysis demonstrates they adopt screw character as plotted in **(f)** and illustrated in the inset. Note: the character of screw and mixed dislocations is determined under the assumption that the sample has negligible thickness.

Figure 4. Crack-tip twinning, omega-phase, and dislocations in $Nb_{45}Ta_{25}Ti_{15}Hf_{15}$. (a) TEM overview micrograph of a fracturing grain boundary ~30 µm ahead of the cracked ligament. **(b)** Virtual dark-field 4D-STEM scans reconstructed from the circled diffraction disk indicated in **(b)** inset show that the deformation at the grain boundary is produced by twinning, with a phase map in **(c)** reconstructed from a virtual detector (highlighted in red) placed over an ω diffraction disk that shows the hexagonal phase on twin boundaries. The stress concentration produced by twins and ω nucleates dislocations, as shown in a STEM image in **(d)**. **(e)** A $g \cdot b$ analysis reveals some dislocations in **(d)** are edges and can travel to form micro-kink bands shown in **(f)**. **(g)** These mechanisms are illustrated in a schematic that shows which part of the HRR field they create. These plots demonstrate that, unlike in tensile samples, the constraint imposed by stress triaxiality does not allow the stresses to become high enough to substantially activate dislocation glide. As a result, fracture occurs in a brittle-like process, shown in **(h)** where the stresses exceed the twinning stresses across a critical microstructural length-scale, the grain size, at the critical stress intensity. Note: the character of screw and mixed dislocations is determined under the assumption that the sample has negligible thickness.

METHODS

SUPPLEMENTARY TEXT

Note 1: Alloy Design

The $Nb_{45}Ta_{25}Ti_{15}Hf_{15}$ alloy was initially designed to balance room-temperature ductility and high-temperature strength, according to ref. [17]. In summary, the original rationale for the chosen composition was to introduce a high concentration of group IV elements to promote ductility, but not too much as to promote the formation of brittle hexagonal close-packed (*hcp*) and omega phases. We did not include Zr because of its tendency to act as a secondary phase former.[44] The Nb/Ta ratio was chosen to promote lattice distortion while simultaneously minimizing the concentration of Ta because of its high density.

While these studies were useful for the high-temperature applications of these alloys, they gave little indication of the low-temperature behavior. DFT and Molecular Dynamics (MD) simulations using a machine-learning atomic potential for this alloy indicated that the screw dislocation glide stresses had well exceeded the measured yield strength at 77 K.[38] Moreover, our experimental observations at 77 K suggested that the slip-twinning transition had already occurred and that the yield strength was controlled by twinning rather than dislocation glide.[17] The combination of experimental observations and simulations motivated the evaluation of the mechanical behavior of this alloy at even lower temperatures to assess the role of this mechanistic transition on the DBTT.

The composition of the $Nb_{70}Ti_{30}$ alloy was chosen to maintain the valence-electron count of $Nb_{45}Ta_{25}Ti_{15}Hf_{15}$ while reducing the chemical complexity to determine whether this variable is important for suppressing the DBTT. Tensile yield strength values in ref. [31] suggested that this

transition could possibly occur in this alloy as well. To further motivate investigating this material, we calculated screw dislocation glide stresses considering a kink width of 10 Burgers vectors and the same methodology and interaction energies given in ref. [40]. We found that the screw glide stresses were slightly lower than those for the multi-principal element alloy: 930 MPa and 1080 MPa at 77 K and 20 K, respectively. The edge dislocation glide stresses calculated using the Maresca-Curtin model at 77 K and 20 K are 75 MPa and 105 MPa, respectively. This provided an opportunity to assess the role of chemical complexity on the DBTT and indirectly evaluate whether the operation of Frank-Reed sources controls the DBTT.[45] Our results suggest that electronic structure is the primary variable controlling this mechanistic transition and the DBTT, and that the relative velocity of screw and edge dislocations does not inhibit dislocation multiplication and glide. This latter point can also be rationalized by the rarity of Frank-Reed sources in single-phase materials.

Note 2: Strain Hardening

The absence of notable strain hardening in tension is also of particular interest, as this property is often associated with high toughness in TWIP and TRIP alloys, exemplified by CrCoNi.[15] In these *fcc* systems, twinning and a phase transformation follow dislocation glide to elevate strain hardening and, in turn, ductility and toughness.[46,47] Conversely, in the *bcc* systems, activation of twinning prior to dislocation glide arises from an intrinsic competition between dislocation motion and dissociation into a twin embryo.[22] This process instantly transforms grains into "multi-lane highways" of varying Schmidt factor (Extended Fig. 5(b)-(c)). As the applied stress increases, glide is progressively favored over twinning, with ~ 50% of the grains in the anti-twinning orientation expected to deform by screw dislocation glide with the original compact core. Dislocations with the lowest slip barriers are selected—specifically those that can cross-slip along twins—channeling them along these boundaries. Omega, as a twin transitional structure, can further promote channeling by preventing dislocation transmission across twin boundaries. In regions where omega spans the distance between adjacent twins (Fig. 3(b)), dislocations can accumulate, similar to materials with pre-existing omega particles.[48] While this hampers uniform ductility, it assists sustained necking, leaving the total fracture strain high and nominally elevating toughness.[17]

Note 3: Role of microstructure on the DBTT

In this note, we address three important microstructural variables that can impact the DBTT, and the resulting limitations this imposes on this study. First, the DBTT is strongly dependent on the concentration of interstitials in all *bcc* alloys. Studies on group IV-V MPEAs suggest that oxygen can have a particularly pronounced impact on strength and ductility.[9] While multiple experiments illustrate that the addition of this interstitial increases room temperature ductility by promoting strain hardening, it can simultaneously raise the DBTT to above 77 K.[9,49] It is also known to raise twinning and phase transformation stresses, thereby suppressing these mechanisms.[21,40] However, this interplay is highly non-linear, *i.e.* O interstitials suppress the ω transformation more relative to twinning.[21] Further work to understand the role in suppressing ω on twin embryo growth and propagation is needed. The relatively low interstitial concentration in $Nb_{45}Ta_{25}Ti_{15}Hf_{15}$ and $Nb_{70}Ti_{30}$ suppresses additional pinning stresses that would otherwise inhibit dislocation glide and twin migration. We postulate that additions of O to the material, likely above 1 at.%, would raise the DBTT to above 20 K.[9,50]

Grain size can also affect the DBTT of *bcc* alloys by raising both dislocation and twinning stresses through the Hall-Petch effect. Coarse grain sizes can be beneficial for increasing ductility and toughness by promoting dislocation glide. However, this only works up to a certain point. In fact, toughness and ductility begin to decrease with grain coarsening past a certain size due to the promotion of strain localization.[51] Moreover, experimental work on multiple alloys indicates that materials undergo the slip-twinning transition across a large temperature range, and that increases in grain size can promote twin propagation, but potentially suppress twin boundary migration.[28,52] In Fe-Si, increasing grain size promotes embrittlement through this mechanism.[28] It is found that, like dislocations, twins obey a Hall-Petch relationship.[21] Further experimental work is needed to assess the Hall-Petch constants.

Finally, recrystallization has been shown to influence the DBTT. This effect is most pronounced in intrinsically brittle materials such as chromium and tungsten, in which extensive hot rolling can introduce high dislocation densities. The presence of these dislocations prevents the need to nucleate them at low temperatures, and due to the inherent presence of kink-pairs, these dislocations can more easily glide at low temperatures. As a result, materials like tungsten and chromium can have their DBTTs suppressed by several 100 K simply by introducing defects that are not entirely removed during recrystallization.[53,54] This effect also increases cryogenic ductility of Nb and its alloys, but in refractory MPEAs it can substantially raise the yield stress due to forest hardening, as is the case in $Nb_{40}Ta_{25}Ti_{15}Hf_{15}Zr_5$.[55,56] When this alloy is not fully recrystallized, the room-temperature yield strength increases by nearly 100 MPa, reflecting limited dislocation glide that would compound at lower temperatures. This effect has been documented in another similar alloy.[57] As a result, partial recrystallization is beneficial by providing the material with already mobile dislocations, but it also introduces additional pinning sites for dislocations, thereby raising the strength and constricting plastic deformation.

Materials processing and microstructural characterization

The $Nb_{45}Ta_{25}Ti_{15}Hf_{15}$ was produced from high-purity elements (>99.9%), which were arc-melted and then flipped and remelted several times to ensure chemical homogeneity. The resulting 12 mm thick ingots were forged and then rolled to ~8 mm (corresponding to a 33% reduction in thickness), and then heat-treated at 1100°C in air in sealed stainless-steel bags for one hour, followed by water quenching. This process was identical to that in ref. 17. The microstructure produced consisted of near-fully recrystallized grains ~259 µm in size.

The $Nb_{70}Ti_{30}$ was produced from high-purity elements (>99.9%) that were arc-melted, then flipped and remelted several times to ensure chemical homogeneity. The resulting 10 mm thick ingots were then cross-rolled to ~6.7 mm (corresponding to a 33% reduction in thickness) and heat-treated at 1077°C in air in sealed stainless-steel bags for 1 hour, followed by water quenching. This heat-treatment temperature was chosen to be the same fraction of the solidus temperature (~0.56) as $Nb_{45}Ta_{25}Ti_{15}Hf_{15}$. The microstructure consisted of a bimodal grain distribution. Some grains were fully recrystallized and averaged ~51 µm, whereas others were partially recrystallized with an average diameter of ~412 µm. These grains were further subdivided by low-angle boundaries consisting of recrystallized rotational twins and kink bands.

To analyze the microstructure of the material, the samples were cut perpendicular to the rolling direction, embedded in resin, and mechanically polished to a surface finish of 0.04 µm. The sample was then analyzed by electron backscatter diffraction in a Thermo Fisher Scios 2 Dual Beam scanning electron microscope (SEM) at an accelerating voltage of 20 kV. Bulk O and N interstitial concentrations were analyzed by inert gas fusion (IGF) and C by combustion.

Mechanical characterization

To evaluate the tensile properties, three rectangular dog-bone specimens with a gauge length of 12 mm were electron-discharge machined (EDM) from the ingots. All sides of the specimen were polished to 1000 grit SiC paper to a final cross-sectional area of 3.13 mm^2. Tensile tests were performed under quasi-static conditions with a strain rate of 10^{-3} s^{-1}. The strain was measured with a 10 mm 3442 clip-on extensometer (Epsilon Technology Corp., Jackson, WY, USA) rated to perform at temperatures as low as ~1 K.

The yield strength, σ_y, ultimate tensile strength, σ_u, uniform ductility, ε, and elongation to fracture, ε_f, were determined from the uniaxial engineering stress-strain curves. Corresponding true stress-strain (σ^T/ε^T) curves were calculated and then compared to instantaneous strain-hardening curves ($d\sigma/d\epsilon$) as a function of strain to evaluate the hardening behavior. A strain-hardening exponent was additionally evaluated using the constitutive power-hardening law $\sigma^T = k(\varepsilon^T)^n$. Here, *n* is the strain-hardening exponent and *k* is a scaling constant. However, the material did not exhibit ideal hardening behavior, and consequently, *n* represents an average hardening value.

To evaluate the fracture properties, two compact-tension C(T) specimens were cut from the ingots by EDM with nominal width *W*=16 mm and thickness *B*=3.5 mm. To prepare the samples for fracture by overload, an atomically sharp crack was inserted into the sample by fatigue pre-cracking. First, both faces were mechanically polished to a final finish of 0.04 μm to allow for crack-length measurement with a long-distance optical microscope. The machined notch, which was ~3.5 mm in length, was then micro-notched with a razor blade coated in a 6 μm diamond paste to a final notch root radius *ρ* ~10 μm. Finally, samples were cyclically loaded in a 4.5 kN servo-hydraulic 810 MTS load frame operated using an Instron 8800 digital controller using load control under a constant-amplitude sinusoidal wave of load ratio *R* = 0.1 (where *R* is the ratio of the minimum to maximum load) at a frequency of 15 Hz. To ensure that the plastic zone around the pre-crack tip did not affect the fracture tests, the stress intensity was gradually reduced such that the stress intensity range, Δ*K*, was 10–12 MPa√m at a final pre-crack length in the range of *a/W* ~ 0.6. Specimens were then side-grooved to a final thickness B_N ~3 mm to ensure the crack followed the mode I loading path.

During the fracture test, the load-line displacement was measured using a 3-mm gauge length Epsilon 3541 COD gauge rated to perform at temperatures as low as ~1 K. To measure the crack length, the sample was systematically unloaded by ~15% of the peak load to measure the elastic unloading compliance. Crack lengths, a_i, were calculated from equations provided by ASTM Standard E1820 for C(T) samples where the compliance is measured on the load-line:[30]

$$a_i/W = 1.000196 - 4.06319u + 11.242u^2 - 106.043u^3 + 464.335u^4 - 650.677u^5 \quad (3)$$

where

$$u = \frac{1}{\sqrt{B_e E C_{C(i)}} + 1}, \quad (4)$$

B_e is the effective thickness ($B_e = B - (B - B_N)^2/B$), E is the elastic modulus, and $C_{C(i)}$ is the rotation corrected compliance. Next, for each crack length, a_i, the *J*-integral was calculated as a sum of the elastic, J_{el}, and plastic, J_{pl}, strain energy contributions:

$$J_{(i)} = J_{el(i)} + J_{pl(i)}, \quad (5)$$

with $J_{\text{el(i)}} = \frac{K_{\text{i}}^2}{E'}$ where $E' = \frac{E}{1-\upsilon^2}$ is the elastic modulus in plane strain conditions (ν is Poisson's ratio) and K_{i} is the linear-elastic stress intensity calculated at each corresponding load-displacement point for C(T) geometry. Finally, the plastic component of J-integral, $J_{\text{pl(i)}}$ is derived from the Merkle-Corten analysis which yields the following expression:[59]

$$J_{\text{pl(i)}} = \left[J_{\text{pl(i-1)}} + \left(\frac{\eta_{\text{pl(i-1)}}}{b_{\text{(i-1)}}}\right)\left(\frac{A_{\text{pl(i)}}-A_{\text{pl(i-1)}}}{B_{\text{N}}}\right)\right]\left[1 - \gamma_{(i-1)}\left(\frac{a_{\text{(i)}}-a_{\text{(i-1)}}}{b_{\text{(i-1)}}}\right)\right] \quad (6)$$

where $\eta_{\text{pl(i-1)}}$ and $\gamma_{\text{(i-1)}}$ are functions of the uncracked ligament, $b_{\text{(i-1)}}$, and $A_{\text{pl(i)}} - A_{\text{pl(i-1)}}$ is the area under the plastic contribution of the load–displacement curve:

$$\gamma_{(i-1)} = 1 + 0.76\, b_{(i-1)}/W \quad (7)$$

$$\eta_{pl(i-1)} = 2 + 0.522\, b_{(i-1)}/W \quad (8)$$

With this formulation, the value of the J-integral at each instantaneous loading-unloading sequence, J_{i}, can be calculated and related to the corresponding crack length. The resulting J–Δa curve can be used to calculate (i) a provisional crack initiation toughness, J_{Q}, by finding its intersection with the 0.2 mm blunting line whose slope is two times the flow stress, $2\sigma_{\text{f}}$; and (ii) a crack-growth toughness which is evaluated at the maximum valid crack extension of Δa=0.25b_0, where b_0 is the initial uncracked ligament. To determine a valid, size-independent fracture toughness, specimens must satisfy plane-strain and J-dominance conditions for validity given by $b_0, B > \frac{10 J_{\text{Q}}}{\sigma_{\text{f}}}$. All specimens met these criteria in $Nb_{45}Ta_{25}Ti_{15}Hf_{15}$ but not all in $Nb_{70}Ti_{30}$. However, in all the latter cases, the specimens were just narrowly above validity, and we did not observe "concave-up" R-curve behavior associated with the transition to non-plane-strain conditions. We therefore believe that the values are valid as well.

To equate these values to the stress intensity, the elastic properties (Young's modulus and Poisson's ratio) were calculated from measurements of the shear and longitudinal sound velocities using a 45MG ultrasonic thickness gauge (Evident Scientific, Waltham, MA, USA). The following equations were then used to extrapolate Poisson's ratio (ν) and Young's modulus (E):

$$\upsilon = \frac{1-2(\frac{V_{\text{T}}}{V_{\text{L}}})^2}{2-2(\frac{V_{\text{T}}}{V_{\text{L}}})^2}, \quad (11)$$

where V_{L} and V_{T} are the longitudinal and transverse velocities, respectively. From these three values, the Young's modulus can then be calculated with:

$$E = \frac{V_{\text{L}}^2 \rho_d (1+\upsilon)(1-2\upsilon)}{1-\upsilon}, \quad (12)$$

where ρ_d is the density measured by the Archimedes method and determined to be 10.8 g/cm^3 for $Nb_{45}Ta_{25}Ti_{15}Hf_{15}$ and 7.4 g/cm^3 for $Nb_{70}Ti_{30}$. The values for the modulus at 77 K of $Nb_{45}Ta_{25}Ti_{15}Hf_{15}$ were obtained from a previous study: $E = 121.8$ GPa and $\upsilon = 0.37$ using values obtained from tensile tests, normalized to the ultrasonic measurements. For $Nb_{70}Ti_{30}$, the elastic properties were obtained at room temperature to be $E = 100.8$ GPa and $\upsilon = 0.386$. We used these values across the entire temperature regime, assuming negligible thermal softening.

Mechanical testing set-up

For the specific purposes of this study, uniaxial tensile and fracture toughness data were obtained with samples cooled with gaseous helium to a temperature of 20 K. Corresponding results

for $Nb_{45}Ta_{25}Ti_{15}Hf_{15}$ at temperatures between 77 K and 298 K, based on similar testing procedures described below, are available in ref. 17.

For the present study, the tensile and fracture toughness tests were performed at the ENGIN-X beamline at the ISIS Neutron and Muon Source at the Rutherford Appleton Laboratory at Harwell Campus, Oxfordshire, in the U.K. The details of this set-up are described in ref. 15. In brief, it consists of an Instron 100 kN rated hydraulic load frame with an Instron 8800 Controller attached to a vacuum-sealed chamber. Two cryo-coolers are connected to the grips with a copper clamp and a series of laminated copper sheets to ensure optimal thermal conductivity. A set of grips and pins made from a high-strength and thermally conductive CuBe alloy were installed into the cold fingers. To further ensure the temperature reached 20 K, thermally conductive copper grease was generally placed in all connective joints. The temperature was measured with two rhodium-iron thermocouples attached to the grips directly adjacent to the samples. Due to the small sample size, it was not possible to attach a thermocouple to the samples without direct interference with the strain field around the crack. To verify that samples reached 20 K, an additional, significantly larger stainless steel C(T) sample with a larger thermal mass (W=18 mm, B=9 mm) was placed in the grips with one of the thermocouples embedded in a copper foil attached to the backside of the sample. Temperatures in the grips reached 8–15 K, while those at the edge of sample reached 28–35 K. We approximate the crack-tip temperature as an average between the two: 18–25 K, which for simplicity we state as 20 K.

Post-*situ* characterization of deformation mechanisms

After testing, the broken samples were immersed in acetone to prevent oxidation. The remaining uncracked ligament in one C(T) sample was fractured by fatigue; the other C(T) sample was cut by EDM at the mid-thickness (B/2). Fractographic analysis was performed on the fractured sample using a Thermo Fisher Scios 2 Dual Beam scanning electron microscope (SEM) at an accelerating voltage of 10 kV using secondary electrons. The sample sectioned at mid-thickness was polished to a final finish of 0.04 μm and imaged in the SEM at an accelerating voltage of 20 kV by electron backscattered diffraction (EBSD) with a step size range of 0.2–1.25 μm. Analysis of EBSD data was performed using Aztec Crystal, a commercially available software.

To understand the grain-boundary chemistry, two sharp needle-like specimens of (i) a fracturing grain boundary and (ii) a grain boundary of a grain that did not undergo intergranular fracture were prepared by the focused ion beam (FIB) lift-out and annular milling techniques using a FEI Helios 5 Hydra UX. 3D atom probe (3DAP) analysis was performed using a local electrode atom probe (CAMECA LEAP 6000 XR) in laser pulse mode with a pulse energy of 60 pJ at a temperature of 70 K.

Scanning transmission electron microscopy (STEM) was carried out to characterize the defect structures in the vicinity of the crack. Site-specific TEM foil samples were cut from the fractured samples and thinned to electron transparency using focused ion beam (FIB) milling (FEI, Helios G4 UX and FEI, Scios 2). Two TEM lamellas were trenched and lifted out from the fracture surface and across grain boundaries near the crack tip from C(T) test specimens. TEM lamella preparation finished with a 5 kV followed by a 2 kV Ga^+ polish. STEM, TEM, HRTEM, and 4D-STEM imaging of samples were conducted on FEI TitanX and FEI ThemIS microscopes (operated at 300 kV) at the National Center for Electron Microscopy (NCEM, LBNL). 0.48 to 1.8 mrad convergence angles were used with an electron probe size of approximately 1.0 to 2.3 nm and a scanning step size of 1.085 to 8.61 nm. 4D-STEM data were analyzed using the py4DSTEM software package.[59] Samples were then examined in the SEM at an accelerating voltage of 30 kV

by transmission Kikuchi diffraction (TKD) with a step size of 10 nm. Samples used to characterize tensile deformation analysis were prepared from tensile samples that were interrupted post-yielding and at uniform ductility by a conventional twin-jet polishing technique, using a solution of 10% H_2SO_4 and 90% CH_4O solution at -45 °C with an applied voltage of 40V.

Crack-tip stress/strain state analysis

To quantify the stress/strain state at the crack-tip, the nonlinear elastic HRR plastic strain field was calculated as a function of the *J*-integral, radial distance, *r*, and angle, *θ*. To perform this calculation, the material was taken to have a power-hardening law. All stresses and strains are true stresses and strains (for simplicity we have removed the "T" superscript):

$$\bar{\varepsilon}_p/\varepsilon_y = \alpha\left(\bar{\sigma}/\sigma_y\right)^N \quad , \tag{9}$$

where ε_y and σ_y are the yield strain and stress, α is a unitless constant (of order unity), and N is the strain-hardening exponent (where $1 \leq N \leq \infty$). For this alloy at 20 K, $N \approx 10$. Note that this equation is the inverse of the preceding power-hardening law. Given these material constants, the HRR field can be calculated from the following expressions:

$$\varepsilon_{ij} = \alpha\varepsilon_y\left[\frac{J}{\alpha\varepsilon_y\sigma_y I_n r}\right]^{\frac{N}{N+1}}\widetilde{\varepsilon_{ij}}(\theta, N), \tag{10}$$

where I_n is an integration constant that is a function of the strain-hardening exponent N, and $\widetilde{\varepsilon_{ij}}(\theta, N)$ is a dimensionless function of strain describing the variation of each strain component as a function of θ and N. The stress can be similarly calculated:

$$\sigma_{ij} = \sigma_y\left[\frac{J}{\alpha\varepsilon_y\sigma_y I_n r}\right]^{\frac{1}{N+1}}\widetilde{\sigma_{ij}}(\theta, N), \tag{11}$$

where similarly, $\widetilde{\sigma_{ij}}(\theta, N)$, is a dimensionless function of stress describing the variations of each stress component as a function of θ and N.

Given that the sample in Fig. 3 was taken 30 μm ahead of the crack-tip ($\theta = 0°$) under plane-strain conditions, the strain tensor, converted to Cartesian coordinates is given as:

$$\varepsilon_{ij} = \begin{bmatrix} -0.741 & 0 & 0 \\ 0 & 0.741 & 0 \\ 0 & 0 & 0 \end{bmatrix} \cdot 10^{-2},$$

with the corresponding stress tensor (in MPa) as:

$$\sigma_{ij} = \begin{bmatrix} 2677.0 & 0 & 0 \\ 0 & 3876.4 & 0 \\ 0 & 0 & 3276.7 \end{bmatrix}.$$

where $\sigma_{33} = \nu(\sigma_{11} + \sigma_{22})$ and $\nu \approx 1/2$ during plastic deformation. In this region, crack-tip stresses are approximately three times higher, and the strains are smaller than those reached in uniaxial tensile samples.

Micromechanical models of stress-controlled fracture for an inverted mechanistic sequence

We constructed a micromechanical model to predict the fracture toughness, K_{Ic}, of *bcc* alloys that undergo a sequence of mechanisms that begins with twinning. Our model assumes that dislocation glide is only activated by twinning and that the screw glide stresses are significantly

higher than the twinning stresses, such that plasticity is solely controlled by twinning and subsequent edge glide. We postulate that semi-brittle intergranular cleavage occurs when the local stress calculated by the HRR field exceeds the upper-bound twinning stress. We first consider the largest difference between the principal stress calculated from the HRR field directly in front of the crack-tip, *i.e.* when $\theta = 0$, to consider effects from constraint.

$$\Delta\sigma_{max} = \sigma_{\theta\theta} - \sigma_{rr} = \sigma_{yy} - \sigma_{xx}\ (\theta = 0),$$

$$\Delta\sigma_{max} = \sigma_{yy} - \sigma_{xx} = \sigma_y \left[\frac{J}{\alpha\varepsilon_y\sigma_y I_n r}\right]^{\frac{1}{N+1}} \widetilde{\sigma_{\theta\theta}}(0, N) - \sigma_y \left[\frac{J}{\alpha\varepsilon_y\sigma_y I_n r}\right]^{\frac{1}{N+1}} \widetilde{\sigma_{rr}}(0, N),$$

$$\Delta\sigma_{max} = \sigma_y \left[\frac{J}{\alpha\varepsilon_y\sigma_y I_n r}\right]^{\frac{1}{N+1}} [\widetilde{\sigma_{\theta\theta}}(0, N) - \widetilde{\sigma_{rr}}(0, N)].$$

Based on our postulate, fracture occurs when $\Delta\sigma_{max}$ exceeds the twinning stress at a critical microstructural length-scale. Given that the upper-bound twinning stress is the yield strength, fracture occurs when $\Delta\sigma_{max} = \sigma_y$ at $r = md_g$, where m is an integer multiple of the grain boundary diameter, d_g.

$$\sigma_y = \sigma_y \left[\frac{J_{Ic}}{\alpha\varepsilon_y\sigma_y I_n m d_g}\right]^{\frac{1}{N+1}} [\widetilde{\sigma_{\theta\theta}}(0, N) - \widetilde{\sigma_{rr}}(0, N)].$$

Re-arranging gives:

$$J_{Ic} = \alpha\varepsilon_y\sigma_y I_n m d_g [\widetilde{\sigma_{\theta\theta}}(0, N) - \widetilde{\sigma_{rr}}(0, N)]^{-(N+1)}.$$

Simplifying by substituting $\varepsilon_y = {}^{\sigma_y}/_{E}$, and converting to the critical stress intensity, *i.e.* $K_{Ic} = \sqrt{{}^{J_{Ic}E}/_{1 - v^2}}$ gives the following expression:

$$K_{Ic} = \sigma_y \sqrt{m d_g \frac{\alpha I_n}{1-v^2} [\widetilde{\sigma_{\theta\theta}}(0, N) - \widetilde{\sigma_{rr}}(0, N)]^{-(N+1)}},$$

to which we assign the factor:

$$\delta(N) = \sqrt{\frac{\alpha I_n}{1-v^2} [\widetilde{\sigma_{\theta\theta}}(0, N) - \widetilde{\sigma_{rr}}(0, N)]^{-(N+1)}}.$$

which leads to the final expression:

$$K_{Ic} = \delta(N)\sigma_y\sqrt{m d_g}\ .$$

All these values can easily be obtained from a tensile test. Approximations can be made; however, we caution that the stress intensity is sensitive to small changes in J_{Ic} values when they are low.

Measurements of the DBTT

The DBTT can be assessed with multiple measurements. Typically, the mechanical properties of a material at the DBTT are characterized by an increase in strength, cessation in tensile ductility, and/or a decrease in fracture toughness. It can also be measured by an examination of the fracture mode with the change from ductile fracture (*e.g.*, microvoid coalescence) to the onset of transgranular cleavage and/or intergranular fracture, thus marking the transition. While the NbTi alloys exhibit a reduction in tensile ductility and toughness from room temperature, and a transition to a nominally intergranular fracture, the materials still have toughness of between ~100–170 MPa√m, and tensile fracture strains of ~14%. The presence of significant plasticity ahead of the crack_tip additionally indicates that the material does not exhibit brittle fracture behavior and thus shows no DBTT.

ADDITIONAL REFERENCES

44. Mills, L. H. *et al.* Temperature-dependent tensile behavior of the HfNbTaTiZr multi-principal element alloy. *Acta Materialia* **245**, 118618 (2023).
45. Lu, Y., Zhang, Y.-H., Ma, E. & Han, W.-Z. Relative mobility of screw versus edge dislocations controls the ductile-to-brittle transition in metals. *Proceedings of the National Academy of Sciences* **118**, e2110596118 (2021).
46. Mahajan, S. & Chin, G. Y. Formation of deformation twins in f.c.c. crystals. *Acta Metallurgica* **21**, 1353–1363 (1973).
47. Kibey, S., Liu, J. B., Johnson, D. D. & Sehitoglu, H. Predicting twinning stress in fcc metals: Linking twin-energy pathways to twin nucleation. *Acta Materialia* **55**, 6843–6851 (2007).
48. Lai, M. J., Li, T. & Raabe, D. ω phase acts as a switch between dislocation channeling and joint twinning- and transformation-induced plasticity in a metastable β titanium alloy. *Acta Materialia* **151**, 67–77 (2018).
49. Lei, Z. *et al.* Enhanced strength and ductility in a high-entropy alloy via ordered oxygen complexes. *Nature* **563**, 546–550 (2018).
50. Huang, A. *et al.* Predicting Interstitial Solutes in Refractory Complex Concentrated Alloys via a Combined Experimental and Computational Workflow: A Case Study on Oxygen in NbTaTiHf. *Acta Materialia* **308**, 122019 (2026).
51. Fan, Z. The grain size dependence of ductile fracture toughness of polycrystalline metals and alloys. *Materials Science and Engineering: A* **191**, 73–83 (1995).
52. Hinrichs, F. *et al.* A ductile chromium–molybdenum alloy resistant to high-temperature oxidation. *Nature* **646**, 331–337 (2025).
53. Bonnekoh, C. *et al.* The brittle-to-ductile transition in cold-rolled tungsten sheets: Contributions of grain and subgrain boundaries to the enhanced ductility after pre-deformation. *Nuclear Materials and Energy* **25**, 100769 (2020).
54. Yoshida, S., Ohba, Y. & Nagata, N. The Effect of Prestraining on the Ductility of Pure Chromium. *Transactions of the Japan Institute of Metals* **1**, 49–53 (1960).
55. Read, D. T. Metallurgical effects in niobium-titanium alloys. *Cryogenics* **18**, 579–584 (1978).
56. Zhang, C. *et al.* Cold-workable refractory complex concentrated alloys with tunable microstructure and good room-temperature tensile behavior. *Scripta Materialia* **188**, 16–20 (2020).
57. An, Y. *et al.* Thermally Stable Heterogeneous-Grained Refractory High-Entropy Alloy Offering Superior Strength from 77 to 973 K. *Nano Lett.* **25**, 15785–15792 (2025).
58. Merkle, J. G. & Corten, H. T. A J Integral Analysis for the Compact Specimen, Considering Axial Force as Well as Bending Effects. *J. Pressure Vessel Technol* **96**, 286–292 (1974).
59. Savitzky, B. H. *et al.* py4DSTEM: A Software Package for Four-Dimensional Scanning Transmission Electron Microscopy Data Analysis. *Microsc Microanal* **27**, 712–743 (2021).

DATA AVAILABILITY

All data that support the findings of this study are reported in the main paper, extended data, and supplementary materials.

ACKNOWLEDGMENTS

The authors appreciate the assistance of N.C. Levkulich, J.O. Brown, and L. Semiatin, Materials Resources LLC, in performing rolling trials in the Metals Processing facility of the Air Force Research Laboratory's Materials and Manufacturing Directorate. Zehao Li is acknowledged for his assistance in APT experiments. The authors would also like to thank Michael Gronley from the machine shop at LBNL for his help and assistance. **Funding:** The research was principally supported by the U.S. Department of Energy, Office of Science, Basic Energy Sciences, Materials Sciences and Engineering Division, through the Damage-Tolerance in Structural Materials program (KC-13) at the Lawrence Berkeley National Laboratory (LBNL) under contract DE-AC02-CH11231. Microscopy facilities were provided by the National Center for Electron Microscopy, in the Molecular Foundry at LBNL, which is supported by the Office of Science, Office of Basic Energy Sciences of the U.S. Department of Energy under contract DE-AC02-05CH11231. Atomistic simulations made use of resources of the National Energy Research Scientific Computing Center (NERSC), which is also supported by the Office of Basic Energy Sciences of the U.S. Department of Energy under the same contract number. C.H.B., E.J.L. and D.A. acknowledge the use of the processing facilities at UC Irvine which is partially supported by the National Science Foundation Materials Research Science and Engineering Center program through the UC Irvine Center for Complex Active Materials (DMR-2011967). E.J.L. also acknowledges the Governor's University Research Initiative and the Chancellor's Research Initiative at Texas A&M University for support. D.H.C. acknowledges support of an NSF Graduate Fellowship from the National Science Foundation (Grant No. DGE 2146752). P.K. acknowledges the support from the Nanyang Technological University Startup Grant (Grant No. 26159-00001). A.D. acknowledges the support from the U.S. Department of Energy, Office of Science, Basic Energy Sciences program, Materials Sciences and Engineering Division as a part of the Early Career Research program FWP 76052 for the atom probe tomography analysis conducted at PNNL. **Author contributions:** R.O.R., D.H.C., and P.K. conceived the project, C.H.B. processed the alloys supervised by E.J.L. and D.A., mechanical testing was carried out by D.H.C., M.O., and P.K. supervised by R.O.R. and D.L., transmission electron microscopy (STEM, TEM, and 4D-STEM) was performed by X.C. and D.H.C supervised by A.M.M. W.W. and P.B. performed the atomistic simulations, supervised by S.R. and M.A. D.H.C. wrote the first draft of the manuscript, with all authors participating in the revision of the final paper. A.D. arranged the APT analysis. R.O.R. and P.K. supervised the overall project. **Competing interests:** The authors declare no competing interests, financial or otherwise. **Data and materials availability:** All data that support the findings of this study are reported in the main paper and Supplementary Materials.

EXTENDED DATA FIGURE LEGENDS

Extended Figure 1. Microstructure of $Nb_{45}Ta_{25}Ti_{15}Hf_{15}$ and $Nb_{70}Ti_{30}$. (a) IPF maps and **(b)** band contrast (BC) maps of $Nb_{45}Ta_{25}Ti_{15}Hf_{15}$ showing a microstructure consisting of equiaxed grains ~259 µm in diameter. Some grains are not fully recrystallized because they contain sub-grain boundaries of partially recrystallized kink bands formed during rolling. **(c)** IPF maps and **(d)** BC maps of $Nb_{70}Ti_{30}$ showing the heterogeneous microstructure consisting of smaller, fully recrystallized grains ~51 µm in diameter and larger grains containing partially recrystallized kink bands and rotational twins. **(E)** and **(F)** show EDX maps indicating homogeneous composition.

Extended Figure 2. Mechanical Properties of $Nb_{70}Ti_{30}$. (a) R-curves showing that the fracture toughness from 298 K to 20 K gradually decreases. **(b)** Representative stress-strain curves across the same set of temperatures show a gradual reduction in ductility as well as twinning-induced stress drops at temperatures at and below 77 K. **(c)** Instantaneous strain-hardening curves demonstrate similar strain-hardening behavior as $Nb_{45}Ta_{25}Ti_{15}Hf_{15}$: hardening becomes less sustained at progressively decreasing temperatures as a result of twin activation at yielding.

Extended Figure 3. Fracture morphology and EBSD maps of $Nb_{70}Ti_{30}$. The effect of stress-state is illustrated by the differences in fracture morphology observed between C(T) and uniaxial tensile samples. Whereas in C(T) specimens in **(a)** fracture occurs by crystallographic microvoids on grain boundaries, in tensile specimens in **(b)** it occurs by microvoid coalescence. This is further exemplified by IPF and BC EBSD maps that show profuse {112}<111> twinning in **(c)** compact-tension and **(d)** tensile specimens, but with significantly more deformation by dislocation glide in tension in **(d)**. The intergranular fracture can also be noted in **(c),** where the crack travels along the high-angle grain boundary between two large grains.

Extended Figure 4. Tensile yielding twins with interfacial ω at 20 and 77 K in $Nb_{45}Ta_{25}Ti_{15}Hf_{15}$. (a) Orientation maps of twins at 20 K taken from a 4D-STEM scan. **(b)** Corresponding virtual dark-field reconstructions of the same twins in **(a)** with the virtual dark-field detector highlighted in red. **(c)** TEM micrograph of twins encased by ω. The corresponding diffraction pattern of the $[\bar{1}13]$ *bcc* zone axis (ZA) shows significantly higher intensities from peaks shared by twins and ω (circled in blue) than those solely from ω. **(d)** Another instance of twinning at 20 K. Dislocations can be seen impinging on the twin boundaries. At 77 K, orientation maps reconstructed from 4D-STEM micrographs show two instances of twinning in **(e)** and **(g)** with corresponding virtual dark-field images reconstructed in **(f)** and **(h).**

Extended Fig. 5. Sequence of tensile deformation mechanisms in $Nb_{45}Ta_{25}Ti_{15}Hf_{15}$. (a) Shows twinning at the onset of yielding, which progressively gives way to deformation by dislocation glide in **(d)**. **(b)-(c)** A simple Schmidt factor analysis of the microstructure shows that the twins can orient each lamella into directions with varying critical resolved shear stress for both $a_0/2${110}<111> and $a_0/2${112}<111> slip systems. While in a typical TWIP material, this would generate strain hardening, here dislocation glide is difficult and plastic deformation has already begun, so only dislocations with the lowest barriers to glide (those with the highest critical resolved shear stresses) will move, causing strain to localize in the material. Severe deformation in the region that necked and directly adjacent to the fracture surface can be seen in **(e)**. Note that the kink band-like features in **(d)** are part of the original microstructure.

Extended Figure 6. Crack-tip kink deformation mechanisms in $Nb_{45}Ta_{25}Ti_{15}Hf_{15}$. (a) Overview EBSD map of crack-tip deformation structure with kink bands boxed in **(b)**, **(c)**, and **(d)**. Pole figure maps of the kink bands in the selected areas in **(a) (**that contain kink bands) show that all have <110> poles, indicating that they are formed by $a_0/2$<111>{112} edge dislocations. The bands have variable rotation: those in **(b)** 8–15°, those in **(c)** 5–17°, and those in **(d)** 10–22°. These kink bands can also have gradient boundaries, indicating imperfect edge dislocation alignment, and can be micrometer-scale in size, as shown in **(b)**. **(e)-(f)** show TKD micrographs of twins impinging on fractured grain boundaries at the fracture surface. The twin in **(e)** has an

unusual morphology characterized by branching.

Extended Figure 7. HRR Stress fields ahead of a crack in $Nb_{45}Ta_{25}Ti_{15}Hf_{15}$ and $Nb_{70}Ti_{30}$. (a) A plot of the HRR field at 20 K as a function of distance from the crack tip normalized by the crack-tip opening displacement, δ. These values are compared with twinning growth stresses and screw and edge glide stresses. As can be seen, the applied stress at the critical stress intensity exceeds the twinning stress across a critical microstructural length-scale - a single grain size. The same occurs at 77 K in **(b)**, demonstrating that the crack-initiation toughness is insensitive to temperature. However, at 77 K, screw glide stresses are lower, which causes this mechanism to activate during crack growth when the stress intensity increases. **(c)**-**(d)** show the local equivalent plastic strain as a function of distance from the crack tip. In $Nb_{70}Ti_{30}$, fracture is strain-controlled because both twinning stresses and dislocation glide stresses are lower than the fracture stress across a critical microstructural length-scale.

Extended Figure 8. Crack-tip twinning, omega, and dislocations in $Nb_{70}Ti_{30}$. (a) HAADF-STEM overview micrograph of a twin-grain boundary intersection site ahead of a crack tip. **(b)** Virtual dark-field 4D-STEM scans reconstructed from the circled diffraction disk indicated in **(b)** inset show that the deformation at the grain boundary is produced by twinning; a phase map in **(c)** reconstructed from a virtual detector (highlighted in red) placed over an ω diffraction disk that shows the hexagonal phase on twin boundaries. Unlike in $Nb_{45}Ta_{25}Ti_{15}Hf_{15}$, the dislocation density is significantly higher, indicating that chemical complexity can substantially increase barriers to dislocation glide and restrict crack-tip plasticity.

Extended Figure 9. Grain-boundary chemistry of $Nb_{45}Ta_{25}Ti_{15}Hf_{15}$. (a) Atom probe tomography (APT) of a grain boundary in this alloy shows an enrichment of group IV elements in a ~4 nm range around the grain boundary. An alloy composition plot in **(b)** shows that the Ti and Hf concentrations are ~42 at.%, compared with their background of 30 at.%. Additional EDS maps of a region where twins and omega impact a grain boundary (from Figure 3) are shown in element maps in **(c)- (f)** and in an HAADF-STEM overview image in **(g)**. A line profile composition shown in **(g)** is plotted in **(h)** and shows a ~4 nm range with slight enrichment of group IV elements and a depletion of group V elements.

Extended Table 1. Summary of mechanical properties of $Nb_{45}Ta_{25}Ti_{15}Hf_{15}$ and $Nb_{70}Ti_{30}$.
