# Supplementary material for: Competing mechanisms toughen bcc binary and medium-entropy alloys at 20 K
Source: Nature. 2026 Oct 14;658(8137):923–9. doi: 10.1038/s41586-026-11065-7 (PMC13686945; doi:10.1038/s41586-026-11065-7)
Supplement: Supplementary file 2 [file 41586_2026_11065_MOESM1_ESM.docx]

Supplementary Information for

**Competing mechanisms ductilize *bcc* binary and medium-entropy alloys at 20 K**

David H. Cook, Xiaoyu Chen *et al.*

Corresponding authors: Dong (Lilly) Liu, dong.liu@eng.ox.ac.uk, Punit Kumar, kpunit@lbl.gov; Robert O. Ritchie, roritchie@lbl.gov

**Supplementary Figure 1. Nb_45_Ta_25_Ti_15_Hf_15_ C(T) specimen fracture surfaces. (a)** Fracture surface at 77 K. The red line indicates the end of the pre-crack; the arrow indicates the direction of crack propagation. The fracture surface is predominantly a mixture of cleavage and microvoid coalescence; a few instances of intergranular fracture are also observed. **(b)** Fracture surfaces at 20 K show a fully intergranular fracture with ductile features, including striations from twinning and microvoids.

**Supplementary Figure 2. Nb_70_Ti_30_ room temperature C(T) specimen fractography and crack-tip deformation mechanisms. (a)** Fracture surfaces show microvoid coalescence which indicates a fully ductile failure. **(b)** IPF and BC maps of crack-tip deformation mechanisms at the mid-thickness show that deformation is controlled by conventional dislocation glide.

**Supplementary Figure 3. Indexed diffraction patterns from Fig. 3.** **(a)** An indexed diffraction pattern from Fig. 2(a) inset shows a reflection across the $[\bar{1}13]$ *bcc* zone axis (ZA) consistent with conventional {112}<111> twinning. **(b)** An indexed diffraction pattern from Fig. 2(b) inset illustrates extra peaks on the $[\bar{1}13]$ *bcc* ZA that corresponds to the $[1\bar{2}13]$ ω ZA. Note: the highlighted red diffraction peaks correspond to the location of the virtual dark-field detector.

**Supplementary Figure 4. *g.b*. analysis of tensile dislocations in Nb_45_Ta_25_Ti_15_Hf_15_.** STEM micrographs of dislocations in Fig. 2 are shown with their corresponding Burgers vectors in **(a)** and **(e)**. Corresponding *g* vectors for **(a)** are shown in **(b)**–**(d)**, whereas corresponding *g* vectors for **(e)** are shown in **(f)**–**(h)**. Most dislocations in **(a)** disappear when $g=[3\bar{1}2]$ indicating that they possess the $[11\bar{1}]$ Burgers vector whereas those in **(e)** disappear when $g=[\bar{1}2\bar{1}]$ which corresponds to a $[111]$ Burgers vector.

**Supplementary Figure 5. Indexed diffraction patterns from Fig. 4.** **(a)** An indexed diffraction pattern from Fig. 4(c) inset illustrates extra peaks on the [011] *bcc* ZA correspond to the $[\bar{1}\bar{1}20]$ ω ZA. **(b)** An additional diffraction pattern from Fig. 4(b) inset shows a reflection across the [011] *bcc* ZA consistent with conventional {112}<111> twinning.

**Supplementary Figure 6.** $\boldsymbol{g\cdot b}$**analysis of crack-tip dislocations in Fig. 4.** STEM micrographs of dislocations in Fig. 4 are shown with their corresponding Burgers vector of ${a_{0}}/2$[111] in **(a)** and **(e)**. Corresponding g vectors for **(a)** are shown in virtual dark-field images in **(b)**–**(d)**, whereas corresponding g vectors for **(e)** are shown in **(f)**–**(h)**. All dislocations disappear in **(a)** and **(e)** when $g=\left[ 1\bar{2}1 \right],$ indicating that they possess the $[111]$ Burgers vector. Since they are edge dislocations, they cannot fully disappear because of the additional non-orthogonal lattice displacement that they produce. An additional imaging condition in **(j)-(l)** confirms they have the $\left[ 111 \right]$Burgers vector and identifies the long straight dislocations above them as being mixed with either $\left[ 11\bar{1} \right]$or $\left[ 111 \right]$ Burgers vectors. Note: the dislocation character analysis in Fig. 4 assumes that the dislocations whose full lengths are cut-off in these micrographs retain the same Burgers vector along their length. There is also a small counterclockwise rotation between **(a)-(h)** and **(i)-(l)**.
